# Proteins that interact with calgranulin B in the human colon cancer cell line HCT-116

**DOI:** 10.18632/oncotarget.14301

**Published:** 2016-12-27

**Authors:** Jae Kyung Myung, Seung-Gu Yeo, Kyung Hee Kim, Kwang-Soo Baek, Daye Shin, Jong Heon Kim, Jae Youl Cho, Byong Chul Yoo

**Affiliations:** ^1^ Department of System Cancer Science, Graduate School of Cancer Science and Policy, National Cancer Center, Goyang, Korea; ^2^ Department of Radiation Oncology, Soonchunhyang University College of Medicine, Cheonan, Korea; ^3^ Colorectal Cancer Branch, Research Institute, National Cancer Center, Goyang, Korea; ^4^ Omics Core, Research Institute, National Cancer Center, Goyang, Korea; ^5^ Department of Genetic Engineering, Sungkyunkwan University, Suwon, Korea; ^6^ Cancer Cell and Molecular Biology Branch, Research Institute, National Cancer Center, Goyang, Korea

**Keywords:** calgranulin B, S100A9, colon cancer, HCT-116, anti-tumor effect

## Abstract

Calgranulin B is released from immune cells and can be internalized into colon cancer cells to prevent proliferation. The present study aimed to identify proteins that interact with calgranulin B to suppress the proliferation of colon cancer cells, and to obtain information on the underlying anti-tumor mechanism(s) of calgranulin B. Calgranulin B expression was induced in colon cancer cell line HCT-116 by infection with calgranulin B-FLAG expressing lentivirus, and it led to a significant suppression of cell proliferation. Proteins that interacted with calgranulin B were obtained by immunoprecipitation using whole homogenate of lentivirus-infected HCT-116 cells which expressing calgranulin B-FLAG, and identified using liquid chromatography-mass spectrometry/mass spectrometry analysis. A total of 454 proteins were identified that potentially interact with calgranulin B, and most identified proteins were associated with RNA processing, post-transcriptional modifications and the EIF2 signaling pathway. Direct interaction of calgranulin B with flotillin-1, dynein intermediate chain 1, and CD59 glycoprotein has been confirmed, and the molecules N-myc proto-oncogene protein, rapamycin-insensitive companion of mTOR, and myc proto-oncogene protein were shown to regulate calgranulin B-interacting proteins. Our results provide new insight and useful information to explain the possible mechanism(s) underlying the role of calgranulin B as an anti-tumor effector in colon cancer cells.

## INTRODUCTION

S100 proteins are a group of 23 different proteins that are characterized by high homology, tissue-specific expression, low-molecular weight, and two calcium-binding EF-hands [1]. Calprotectin [heterodimer of calgranulin A (S100A8) and B (S100A9)] was discovered as an immunogenic protein expressed by neutrophils with potent anti-microbial properties [1–3]. Elevated calgranulin A and B protein levels are a feature of numerous pathological conditions associated with inflammation, including rheumatoid arthritis, systemic lupus erythematosus, multiple sclerosis, cystic fibrosis, giant cell arteritis, psoriasis, and chronic inflammatory bowel diseases [1, 3, 4]. The association between inflammation and cancer pathogenesis has long been known, and it was assumed that infiltrating leukocytes represent an attempt by the host to destroy cancer cells [5, 6].

Recent pre-clinical and clinical data have suggested that alterations in the expression and/or function of S100 proteins may represent an important step in cancer development [1]. Up-regulation of calgranulin B has been reported in numerous cancer types including breast, lung, liver, uterine, cervical, ovarian, gastric, esophageal, pancreatic, and colorectal cancers [7, 8]. Furthermore, changed expression of calgranulin B was related to poor tumor differentiation in carcinomas of glandular cell origin, such as those of the breast, lung, and thyroid gland [8–11].

In our previous reports, the level of calgranulin B was significantly higher in stools of colorectal cancer patients compared to controls [12]. A combined analysis of two fecal markers, calgranulin B and the fecal occult blood test, showed greater sensitivity and specificity for colorectal cancer than the fecal occult blood test alone [13]. Recently, we found that calgranulin B released from immune cells such as neutrophils, can be internalized specifically into colon cancer cells [14]. Calgranulin B internalization induced apoptosis signaling and reduced cell proliferation, possibly through binding to and inhibiting aurora A kinase [14].

The present study aimed to identify proteins that interact with calgranulin B to suppress the proliferation of colon cancer cells, and to identify the molecular mechanisms underlying the anti-tumor effects of calgranulin B.

## RESULTS

### Suppressed cell proliferation after calgranulin B expression via lentivirus infection

**Figure 1:**
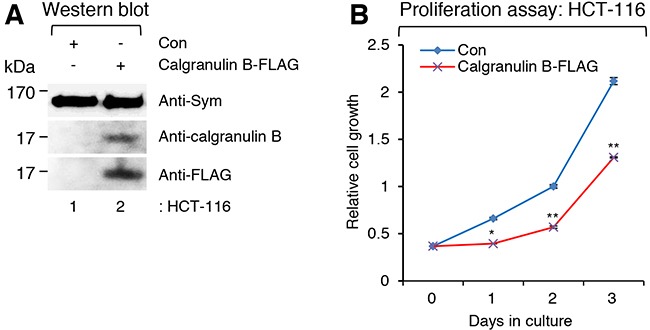
Suppressed colon cancer cell proliferation caused by expression of calgranulin B after lentivirus infection **A**. Western blot (WB) analysis showing the expression of calgranulin B-FLAG in the colon cancer cell line HCT-116 after infection of pLenti6-calgranulin B-FLAG. As a positive loading control, symplekin (Sym) was detected in both pLenti6-calgranulin B-FLAG- and pLenti6-Con-infected HCT-116 cells. Expression of calgranulin B-FLAG after infection of pLenti6-calgranulin B-FLAG was confirmed with either the anti-calgranulin B or the anti-FLAG (DYKDDDDK) antibody. **B**. Suppressed HCT-116 cell proliferation after infection of calgranulin-expressed lentivirus. The proliferation of HCT-116 cells infected with pLenti6-calgranulin B-FLAG was significantly reduced compared to that of pLenti6-Con-infected HCT-116 cells. Data represent the mean values of at least three independent experiments performed in triplicate. Error bars in the graph represent ± SD. *p<0.05 and **p<0.01.

To evaluate the effect of calgranulin B expression on colon cancer cell proliferation, calgranulin B expression was artificially induced in the colon cancer cell line HCT-116 using pLenti6-calgranulin B-FLAG. Expression of calgranulin B-FLAG after infection of pLenti6-calgranulin B-FLAG was confirmed with either the anti-calgranulin B or anti-FLAG (recognizes DYKDDDDK epitope) antibody, and the positive loading control symplekin was detected in both pLenti6-calgranulin B-FLAG- and pLenti6-Con-infected HCT-116 cells (Figure 1A). As shown in Figure 1B, calgranulin B expression after lentiviral infection of pLenti6-calgranulin B-FLAG significantly suppressed HCT-116 cell proliferation.

### Identification of calgranulin-interacting proteins

**Figure 2:**
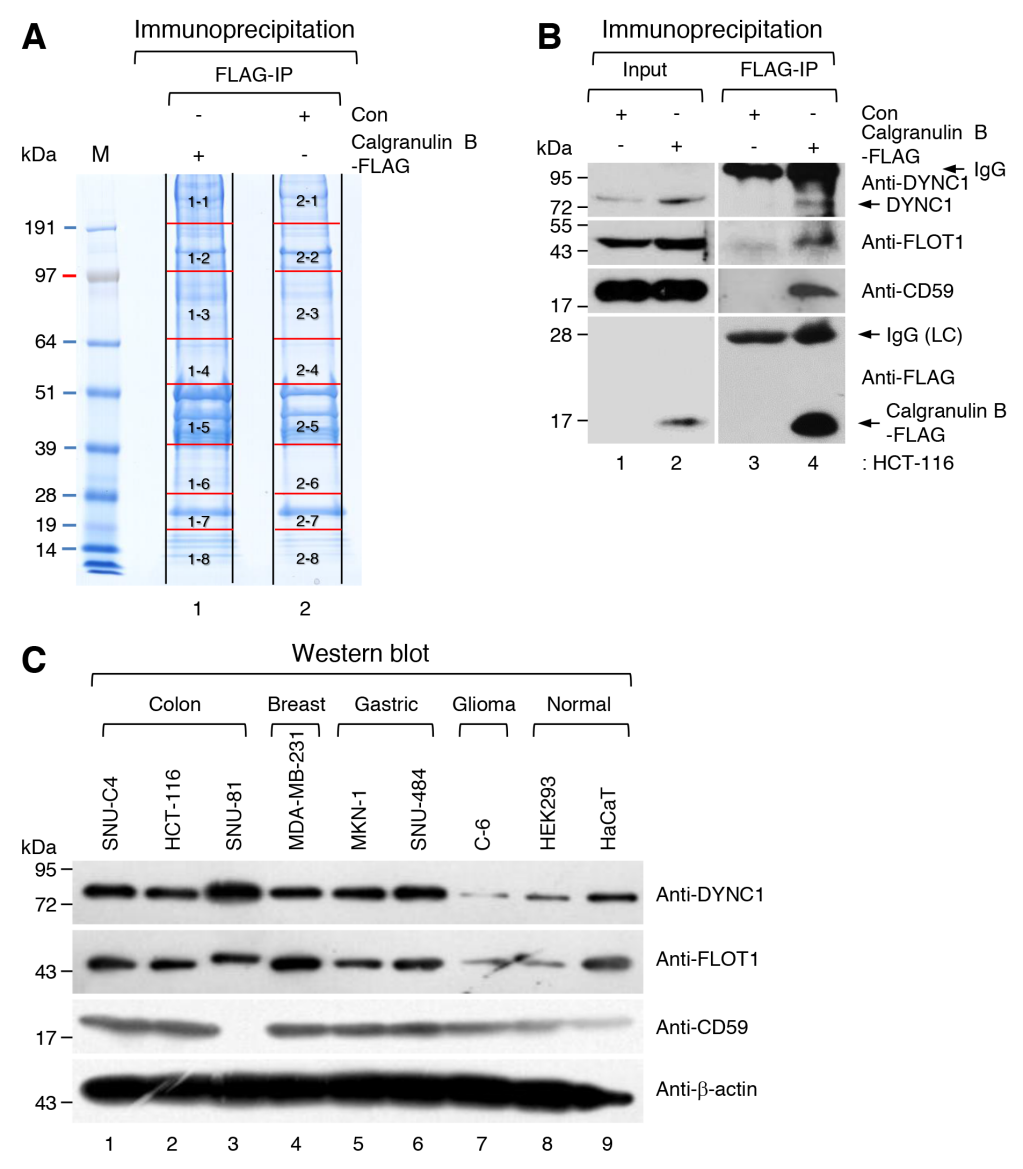
Identification of candidate calgranulin B-interacting proteins **A**. Sodium dodecyl sulfate-polyacrylamide gel electrophoresis (SDS-PAGE) of immunoprecipitates. Whole homogenates of pLenti6-calgranulin B-FLAG-infected and pLenti6-Con-infected HCT-116 cells and anti-FLAG M2 affinity gel were used for immunoprecipitation (IP). The SDS-PAGE gel was numbered and sliced as shown, and all proteins in the slice were identified using liquid chromatography-mass spectrometry/mass spectrometry analysis (LC-MS/MS) as described in the MATERIALS AND METHODS. All candidate calgranulin B-interacting proteins are listed in Supplementary Table 1. **B**. Whole homogenates of pLenti6-calgranulin B-FLAG-infected and pLenti6-Con-infected HCT-116 cells and anti-FLAG M2 affinity gel were used for IP. WB performed with dynein intermediate chain 1 (DYNC1), flotillin-1 (FLOT1), CD59, and FLAG-tag specific antibodies. IgG; immunoglobulin, LC; light chain. **C**. WB analysis of FLOT1, DYNC1, and CD59 in various cancer cell lines and normal cell lines. FLOT1, DYNC1, and CD59 were identified as calgranulin B-interacting proteins (Supplementary Table 1), and the expression of these proteins was relatively higher in cancer cell lines compared to normal cell lines.

To identify candidate proteins that interact with calgranulin B to suppress cell proliferation, whole homogenates of pLenti6-calgranulin B-FLAG- and pLenti6-Con-infected HCT-116 cells were incubated with anti-FLAG M2 affinity gel and immunoprecipitated. All proteins in two different immunoprecipitates were separated using sodium dodecyl sulfate-polyacrylamide gel electrophoresis (SDS-PAGE). The SDS-PAGE gel was sliced as shown in Figure 2A, and all proteins in the slice were identified using liquid chromatography-mass spectrometry/mass spectrometry (LC-MS/MS) analysis.

Calgranulin B-interacting proteins were selected by removing overlapping proteins in the immunoprecipitate of pLenti6-Con-infected HCT-116 cells from proteins in the immunoprecipitate of pLenti6-calgranulin B-FLAG-infected HCT-116 cells. A total of 454 proteins were identified as candidates, which are listed in red in Supplementary Table 1. The candidate proteins included caveolae-associated integral membrane protein, flotillin-1 (FLOT1); transport cargo-associated protein, dynein intermediate chain 1 (DYNC1); and CD59 glycoprotein (CD59), which may give a clue specific internalization of calgranulin B into colon cancer cells.

The interactions calgranulin B with identified proteins (FLOT1, DYNC1, and CD59) which may cooperate on the internalization, were re-confirmed with calgranulin B-FLAG immunoprecipitation coupled western blotting with specific antibodies of those molecules. As shown in Figure 2B, endogenous FLOT1, DYNC1, and CD59 were efficiently precipitated with calgranulin B-FLAG. However, those proteins did not show any differential expression in colon cancer cell lines compared other types of cancer cell lines (Figure 2C).

We also performed proliferation assay after knockdown of these identified molecules with specific siRNAs whether the identified calgranulin B-interacting proteins are related to anti-tumor effect. Expression of two molecules (CD59 and FLOT1) except DYNC1 was interfered by siRNA transfection (Supplementary Figure 1). However, knockdown of those molecules did not give any benefit for the proliferation of calgranulin B-FLAG expressing HCT-116 cells.

### GO analysis, and top diseases and disorders information

**Figure 3:**
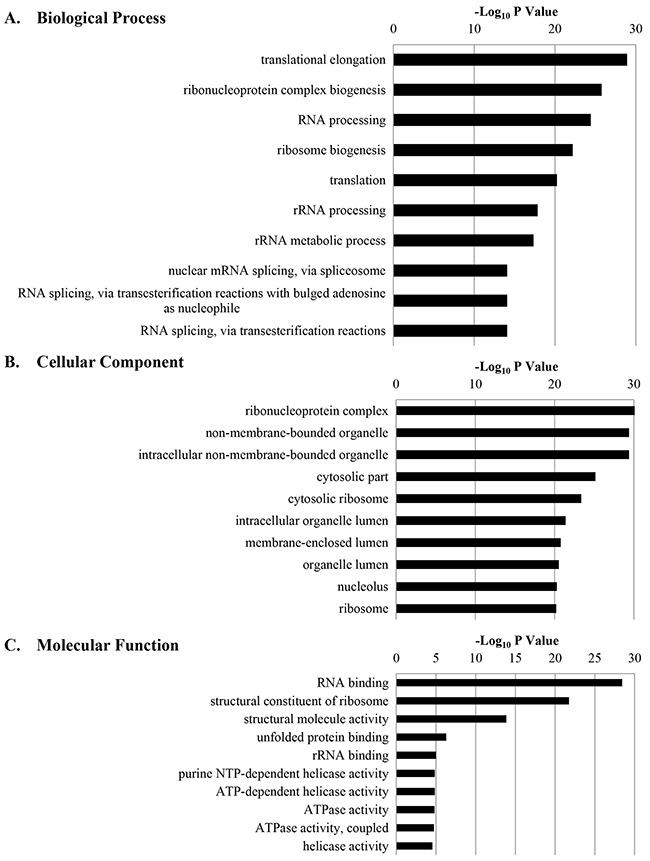
The 10 most significantly regulated gene ontology (GO) categories for calgranulin B-interacting proteins according to DAVID GO enrichment analysis All calgranulin B-interacting proteins were categorized according to their biological process **A**. cellular component **B**. and molecular function **C**. GO terms, and ten most significant terms are shown.

All GO terms associated with the biological processes, cellular components, and molecular functions of calgranulin B-interacting proteins were generated using DAVID, and the 10 most significantly regulated GO terms are shown in Figure 3. Information concerning all molecules involved in each GO term is shown in Supplementary Table 2.

GO analysis revealed 33 biological processes that calgranulin B-interacting molecules were involved in; the top five were translational elongation, ribonucleoprotein complex biogenesis, RNA processing, ribosome biogenesis, and translation.

The cellular component of ontology describes the location, levels of subcellular structure, and macromolecular complexes of proteins (www.geneontology.org). The identified calgranulin B-interacting molecules were localized mostly in ribonucleoprotein complexes, non-membrane-bound organelles, intracellular non-membrane-bound organelles, the cytosol, and cytosolic ribosomes.

DAVID analysis also revealed that RNA-binding, structural constituents of ribosome, structural molecule activity, unfolded protein binding, and ribosomal RNA-binding were the most relevant molecular functions of calgranulin B-interacting molecules.

Information concerning the top diseases and disorders that calgranulin B-interacting molecules from a colorectal cancer cell line were involved in was obtained from QIAGEN's IPA (QIAGEN, Redwood City, CA, USA; www.qiagen.com/ingenuity). Most identified calgranulin B-interacting molecules were found in cancer, followed by organismal injury and abnormalities, tumor morphology, infectious disease, and cardiovascular disease (Table 1).

**Table 1:** Information concerning the diseases and disorders identified using ingenuity pathway analysis (IPA) for calgranulin B-interacting proteins

Name	*p*-value	# Molecules
Cancer	9.54E-03 – 2.40E-19	378
Organismal Injury and Abnormalities	9.54E-03 – 2.40E-19	384
Tumor Morphology	7.06E-03 – 2.40E-19	54
Infectious Diseases	7.03E-03 – 2.48E-14	107
Cardiovascular Disease	7.05E-03 – 2.56E-08	29

### Canonical pathway analysis

**Figure 4:**
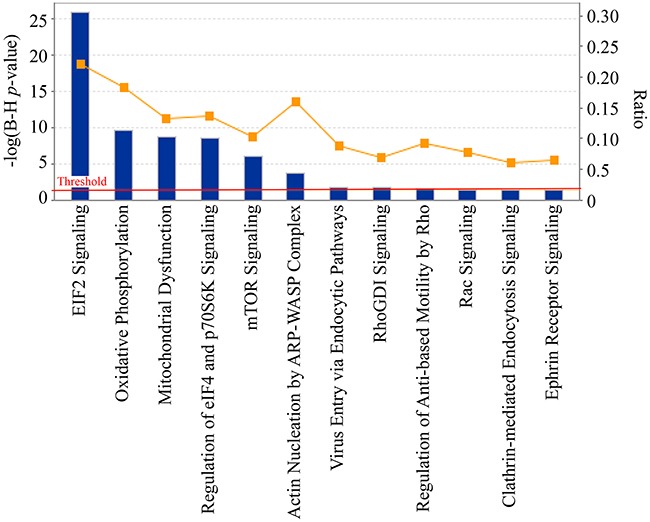
Significant canonical pathways of calgranulin B-interacting proteins identified by ingenuity pathway analysis (IPA) The most significant canonical pathways of the calgranulin B-interacting molecules [-log(B-H *p*-value) > 1.3 and threshold value = 0.05] are shown. The left y-axis corresponds to the negative logarithm of the *p*-value after Benjamini-Hochberf (B–H) multiple testing correction [-log(B-H *p*-value)]. The right y-axis corresponds to the orange curve graph representing the ratio between the total number of calgranulin B-interacting molecules and the total number of molecules in a given pathway that met the cutoff criteria.

IPA was applied to the identified calgranulin B-interacting proteins. A total of 431 proteins (23 proteins were excluded due to overlap or a missed gene symbol) were mapped to IPA and proteins not mapped to the IPA database were excluded in pathway analyses; 165 significant canonical pathways were identified (B–H adjusted *p*-value < 0.01; see Supplementary Table 3). The five signaling pathways most associated with calgranulin B interacting molecule enrichment were: the EIF2 signaling pathway (ratio = 2.22E-01, -log (B–H *p*-value) = 2.59E01), oxidative phosphorylation (ratio = 1.83E-01, -log (B-H *p*-value) = 9.59E00), mitochondrial dysfunction (ratio = 1.33E-01, -log (B-H *p*-value) = 8.68E00), regulation of eIF4 and p70S6K signaling (ratio = 1.36E-01, -log (B-H *p*-value) = 8.53E00), and mTOR signaling (ratio = 1.03E-01, -log (B-H *p*-value) = 5.97E00) (Figure 4).

### Biological relationships of calgranulin B-interacting molecules measured by network analysis

**Figure 5:**
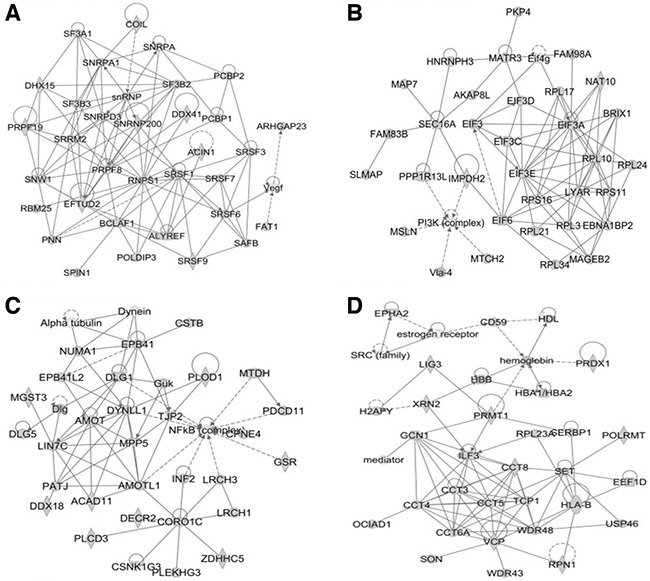
The top four networks of calgranulin B-interacting proteins identified by IPA (score > 45) The four networks most significantly associated with calgranulin B-interacting proteins, determined using IPA, were as follows: RNA post-transcriptional modification, infectious diseases, organismal injury and abnormalities (**A**. score = 54); cancer, cell death and survival, organismal injury and abnormalities (**B**. score = 49); cardiovascular system development and function, cell death and survival, cell morphology (**C**. score = 46); and cellular assembly and organization, cell-to-cell signaling and interaction, reproductive system development and function (**D**. score = 46).

In this study, all identified calgranulin B-interacting molecules and hypothetical interacting genes stored in the knowledge base of the IPA software were used to generate a set of networks with a maximum network size of 35 genes/protein. Networks were displayed graphically as genes/gene products (“nodes”) and displayed the biological relationships between the nodes (“edges”). All edges were obtained from canonical information stored in the IPKB. The networks of the genes were generated using IPA based on their connectivity and each was ranked. The top-scoring biological network obtained using IPA analysis represented a cluster of highly significant proteins. Ten networks were identified with scores ranging from 35 to 54, of which the top four were associated with the following: 1) RNA post-transcriptional modification, infectious diseases, and organismal injury and abnormalities (score = 54) (Figure 5A); 2) cancer, cell death and survival, and organismal injury and abnormalities (score = 49) (Figure 5B); 3) cardiovascular system development and function, cell death and survival, and cell morphology (score = 46) (Figure 5C); and 4) cellular assembly and organization, cell-to-cell signaling and interaction, and reproductive system development and function (score = 46) (Figure 5D). The network information of calgranulin B-interacting molecules and the corresponding top disease and function information are shown in Supplementary Table 4.

### Identification of upstream regulators

Upstream regulator analysis is used to identify potential upstream regulators of proteins, including transcription factors, as well as any gene or small molecule observed experimentally to affect gene expression by analyzing linkages to genes through coordinated expression. IPA identified 285 potential upstream regulators (Supplementary Table 5). The top upstream activated regulator was predicted to be the transcription regulator N-myc proto-oncogene protein (MYCN) (*p*-value of overlap = 6.63E-24), whose target molecules among the calgranulin B-interacting proteins are cytoskeleton-associated protein 4 (CKAP4), eukaryotic elongation factors (EEFs), high mobility group AT-hook1 (HMGA1), the integrin family (ITGA3, ITGB1), galectins (LGALS1), prohibitin (PHB), retinoblastoma binding protein 4 (RBBP4), L ribosomal proteins (RPLs), S ribosomal proteins (RPS), and calcium-binding protein A10 (S100A10). Rapamycin-insensitive companion of mTOR (RICTOR) and myc proto-oncogene protein (MYC) were identified as additional activated upstream regulators (*p*-value of overlap = 4.09E-23 and 1.53-21, respectively) and their activation was predicted to regulate 39 and 72 calgranulin B-interacting proteins, respectively.

Calgranulin B was the target of multiple molecular functions, including the following: transcription regulators such as hepatocyte nuclear factor 4 alpha (HNF4A), proto-oncogene c-Fos (FOS), *trans*-acting T-cell-specific transcription factor GATA-3 (GATA3), and myocardin-like protein 1 (MKL1); kinases including cyclin-dependent kinase inhibitor 1B (CDKN1B) and TEK receptor tyrosine kinase (TEK); peptidases including disintegrin and metalloproteinase with thrombospondin motifs 1 (ADAMTS1), kallikrein 5 (KLK5) and urokinase-type plasminogen activator (PLAU); cytokines including interleukin-4 (IL4) and interleukin 17C (IL17C); growth factors including hepatocyte growth factor (HGF), brain-derived neurotrophic factor (BDNF) and inhibin beta A chain (INHBA); the ligand-dependent nuclear receptor ESR1; and barrier-to-autointegration factor (BANF1) and zinc finger protein 184 (ZNF184).

### Protein-protein interaction network analysis of calgranulin B-interacting molecules

**Figure 6:**
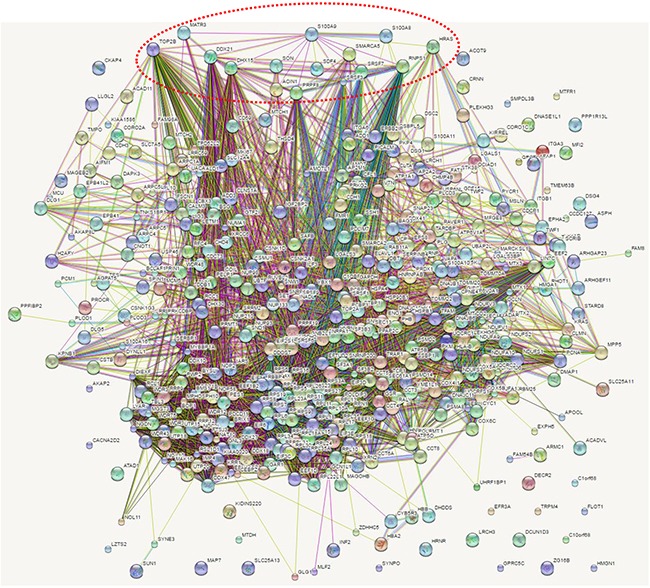
Network analysis of calgranulin B-interacting molecules Molecular interactions in networks were determined via STRING analysis and direct interactions with calgranulin B are represented by a red dotted circle.

To determine a more comprehensive view of the molecular network of calgranulin B-interacting molecules, direct and indirect molecular interactions were analyzed using STRING, as shown in Figure 6. Molecular interactions in the network were connected with lines and direct interactions of calgranulin B with other proteins were marked in red circles.

Calgranulin B (S100A9) was shown to interact directly with S100A8 (score 0.999 from co-expression, experimental, database, text mining data), as reported previously [15–17], and was also shown to interact with 45-kDa calcium-binding protein (SDF4, score 0.472 from text mining data), GTPase HRas (HRAS, score 0.480 from text mining data), apoptotic chromatin condensation inducer 1 (ACIN1, score 0.575 from experimental data), pre-mRNA-splicing factor ATP-dependent RNA helicase DHX15 (DHX15, score 0.583 from experimental data), pre-mRNA-processing-splicing factor 8 (PRPF8, score from experimental data), matrin-3 (MATR3, score 0.644 from experimental data), RNA-binding protein with serine-rich domain 1 (RNPS1, score 0.689 from experimental and text mining data), nucleolar RNA helicase 2 (DDX21, score 0.574 from experimental and text mining data), SWI/SNF-related matrix-associated actin-dependent regulator of chromatin subfamily A member 5 (SMARCA5, score 0.561 from experimental data), Serine/arginine-rich splicing factor 7 (SRSF7, score 0.580 from experimental data), Serine/arginine-rich splicing factor 3 (SRSF3, score 0.595 from experimental and text mining data), protein SON (SON, score 0.580 from experimental data), and DNA topoisomerase 2-beta (TOP2B, score 0.577 from experimental and text mining data).

## DISCUSSION

Calprotectin, a heterodimer of calgranulin A (S100A8) and calgranulin B (S100A9), is involved in various inflammatory and neoplastic disorders [1, 7]. However, calprotectin seems to be a Janus-faced molecule in the context of cancer [3]. While calprotectin expression in cancer cells is associated with tumor development, cancer invasion, and metastasis, it is also a powerful apoptotic agent produced by immune cells that may play a pivotal role as a cancer-selective agent [3]. Compared with the pro-tumor effects of calprotectin, the effects of calgranulin B alone have not been well studied. Our previous study revealed that colon cancer cell lines were not able to express calgranulin B because of CpG methylation in the promoter region of the calgranulin B gene, and internalization of extracellular calgranulin B into colon cancer cells suppressed cell proliferation and induced apoptosis [14]. In line with our previous results, infection of pLenti6-calgranulin B-FLAG induced the expression of calgranulin B in the colon cancer cell line HCT-116 (Figure 1A) and significantly suppressed cell proliferation (Figure 1B). Therefore, it is clear that calgranulin B is an important molecule that can induce anti-tumor effects in colon cancer cells.

To determine the molecular mechanism(s) underlying the cell proliferation effects of calgranulin B in colon cancer cells, an interactome approach was taken using calgranulin B immunoprecipitate, and 454 proteins were identified as calgranulin B-interacting candidate proteins (Figure 2A, Supplementary Table 1). The majority of the identified calgranulin B-interacting candidate proteins were involved in cancer (Table 1). For example, flotillin-1 (FLOT1), DYNC1, and CD59 were overexpressed in colon, breast, and gastric cancer cell lines compared to normal cell lines (Figure 2C). Like caveolin-1, flotillin-1 is a major structural protein associated with lipid rafts in mammalian cells [18]. Unfortunately, in our previous study, we were unable to fully determine the internalization pathway of extracellular calgranulin B into colon cancer cells, but established that this pathway is reliant on the type of colon cancer cell [14]. For example, internalization of extracellular calgranulin B into HCT-116 was not affected by the endocytosis inhibitors CPZ (clathrin-mediated endocytosis), MßCD (caveolae/lipid raft-mediated endocytosis), or Cyto D (macropinocycosis) [14]. Therefore, while flotillin-1 may interact with calgranulin B, it does not appear to be a key player in the internalization of only calgranulin B into colon cancer cells. Cytoplasmic dynein plays a role in the regulation of mitotic checkpoints by moving critical checkpoint components off kinetochores; therefore, human cells depleted of DYNC1 have delayed metaphase with increased interkinetochore distances [19]. Our previous study showed that calgranulin B interacts with aurora A kinase, leading to its inhibition [14]. Aurora A kinase is required for centrosome maturation and centrosomal anomalies have been demonstrated in tumor formation and progression [20]. Calgranulin B interaction with aurora A kinase and DYNC1 increases our understanding of how internalized calgranulin B may affect tumor cell division. Calgranulin B showed a positive correlation with stromal inflammatory cells surrounding colon cancer cells [14], and up-regulated CD59 has been linked to differentiation and TNM staging of colon cancer [21]. Therefore, calgranulin B, CD59, and their interactions may be useful for molecular staging diagnoses and colon cancer therapies.

The eIF2 signaling pathway was the most relevant signaling pathway in which calgranulin B-interacting proteins were involved (Figure 4, Supplementary Table 3). The eukaryotic initiation factor eIF2 is a key component of the ternary complex, the role of which is to deliver initiator tRNA into the ribosome [22]. A variety of stimuli, both physiological and pathophysiological, activate eIF2 kinases that phosphorylate the α subunit of eIF2, preventing it from forming the ternary complex and thus attenuating cellular protein synthesis [22]. Paradoxically, in cancer cells, the phosphorylation of eIF2α is associated with activation of survival pathways [22]. Presently, little is known regarding the link between calgranulin B and the eIF2 signaling pathway for cancer cell survival, and further studies are needed to define this link. Interestingly, other signaling pathways other than eIF2 signaling support the role of aurora A kinase identified in the previous study [14]. Oxidative phosphorylation, mitochondrial dysfunction and mTOR signaling found in this study are known to be involved in aurora kinase inhibition. Aurora A kinase is a potential oncogene that activates mTOR/Akt pathway in the process of cell transformation [23]. mTOR was shown to be relocated to mitochondria and enhances oxidative phosphorylation and reduces glycolysis in HCT-116 [24]. Accumulating evidence suggests that mitochondrial dysfunction induces apoptosis, which kill cancer cells by aurora kinase inhibition [25] and Sun and co-workers also revealed that aurora kinase inhibition led to induction of PUMA, a BH3-only Bcl-2 family protein which mediates the apoptosis initiation in colon cancer cells via mitochondrial pathway [26]. Aurora A kinase still remains one of the most important pathways which regulate proteins interacting with calgranulin B and further functional studies may elucidate more molecular mechanism in colon cancer.

Many calgranulin B-interacting proteins were involved in translation elongation and were localized in the ribonucleoprotein complex (Figure 3, Supplementary Table 2). Calgranulin B-interacting proteins seem to be associated with RNA processing, as they were found to have the molecular function of binding RNA, and the most relevant network analysis revealed RNA post-transcriptional modification functions (Figure 5, Supplementary Table 4). Calgranulin B was shown to interact directly with S100A8, ACIN1, DHX15, PRPF8, MATR3, DDX21, and SON (Figure 6). All proteins that directly interacted with calgranulin B were revealed to be involved in RNA processing (Figures 3 and 6). For example, ACIN1, an RNA-binding protein originally identified for its role in apoptosis, plays a role in splicing regulation as well as in other cellular pathways, including cell cycle progression [27]. DHX15, PRPF8, SRSF10, and SON play critical roles in modulating pre-mRNA splicing [28–31]. In addition, MATR3 has a role in mRNA stabilization [32], and DDX21 coordinates transcription and ribosomal RNA processing [33]. The overall results obtained from bioinformatic analysis demonstrate that the first target of calgranulin B to suppress colon cancer cell proliferation may be RNA processing.

MYCN, MYC, and RICTOR were determined to be upstream regulators of calgranulin B-interacting proteins (Supplementary Table 5). The MYC oncogene has long been established as a central driver of many types of human cancers, including colorectal cancer [34]. A recent study showed that aurora A kinase and the targeting protein for Xklp2 (TPX2) are novel co-regulators of the MYC pathway, suggesting that targeting of the aurora A kinase/TPX2 axis could be a therapeutic approach for MYC-driven cancers [34]. In line with this report, we showed previously that calgranulin B can inhibit aurora A kinase [14]. The mTOR pathway integrates nutrient- and growth factor-derived signals to regulate growth, i.e., the process whereby cells accumulate mass and increase in size [35]. mTOR is a large protein kinase and is the target of rapamycin, an immunosuppressant that also blocks vessel restenosis and has potential anticancer applications [35]. The RICTOR-mTOR complex modulates the phosphorylation of protein kinase C alpha and the actin cytoskeleton [35], but the molecular underpinnings of RICTOR remain poorly understood.

In conclusion, the results of our present study provide new insights to explain the possible molecular mechanism(s) underlying the anti-tumor effects of calgranulin B in colon cancer cells.

## MATERIALS AND METHODS

### Cell lines

Human cancer cell lines (colon cancer cell lines: SNU-81, SNU-C4, and HCT-116; breast cancer cell line: MDA-MB-231; gastric cancer cell lines: MKN-1 and SNU-484; glioma cell line: C-6) and normal cell lines (HEK293 and HaCaT) were obtained from the Korean Cell Line Bank (KCLB) (Seoul, Korea). 293FT cells were purchased from Life Technologies (Carlsbad, CA, USA).

### Plasmids

For the generation of pcDNA3-clagranulin B-FLAG, polymerase chain reaction (PCR) was performed with pcDNA3.1(+)-calgranulin B (B.C.Y., personal communication) as a template with the following oligomers: sense, 5′-CGGGATCCGCCACCATGACTTGCAAAATGTCGCAG-3′ and antisense, 5′-GCTCTAGACCGGGGGTGCCCTCCCCGAG-3′. The amplified DNA fragment was subcloned into BamHI-XbaI treated pcDNA-cFLAG (J.H.K., personal communication). pcDNA3-calgranulin B-FLAG was used as a PCR template for the construction of the final pLenti6-calgranulin B-FLAG construct with the following oligomers: sense, 5′-AGT GTGGTGGAATTCGCCACCATGACTTGCAAAATGTC G-3′ and antisense, 5′-CCCTCTAGACTCGAGGGTACCGACTCGAGTTAGG-3′. The amplified DNA fragment was inserted into the EcoRI-XhoI treated pLenti6-Con (J.H.K., personal communication) using the InFusion reaction (Takara, Shiga, Japan). All oligomers were purchased from Macrogen (Seoul, Korea) and all constructs were verified by DNA sequencing (Cosmo Genetech, Seoul, Korea).

### Lentivirus production and infection

293FT cells (2.5 × 10^6^; Life Technologies) were plated on 100 mm culture dishes 24 h before transfection. The lentiviral construct (4.5 μg; pLenti6-Con and pLenti6-calgranulin B-FLAG), 3 μg of psPAX2 (Addgene, Cambridge, MA, USA; #12260), and 1.5 μg of pMD2.G (Addgene; #12259) were co-transfected into 293FT cells using 27 μL of METAFECTENE® PRO (Biontex, Munich, Germany). The Opti-MEM® medium (Life Technologies) containing transfectant was changed to a medium without antibiotics 5 h after transfection. The medium containing the lentivirus was harvested 48-72 h after transfection and was used directly for each infection. HCT-116 cells were infected with these lentiviruses in the presence of 10 mg/mL of polybrene (Sigma-Aldrich, St. Louis, MO, USA) for 9-15 h and further selection was performed with blasticidin S treatment (5 μg/mL; InvivoGen, San Diego, CA, USA) for 3-4 days.

### Antibodies and western blotting

The anti-calgranulin B (Santa Cruz Biotechnology, Dallas, TX, USA), anti-symplekin (BD Transduction Laboratories, San Jose, CA, USA), anti-FLAG (anti-DYKDDDDK; BioLegend, San Diego, CA, USA), anti-flotillin-1 (Abcam, Cambridge, UK), anti-dynein intermediate chain 1 (DYNC1) (Abcam), anti-CD59 (Abcam), and anti-β-actin (Abcam) antibodies were used for western blot (WB) analyses. Horseradish peroxidase-conjugated anti-rabbit (Vector Laboratories, Burlingame, CA, USA), anti-mouse (Vector Laboratories), and anti-rat immunoglobulin (Santa Cruz Biotechnology) secondary antibodies were used.

### Proliferation assay and statistical analysis of data

Proliferation assays were performed using the CyQUANT® NF cell proliferation assay kit (Life Technologies) according to the manufacturer's protocol. Proliferation assay data are presented as the mean ± standard deviation (SD) determined from minimum three independent experiments. Differences were assessed by the two-tailed Student's t-test using Excel software (Microsoft). *p≤0.05* was considered as statistically significant.

### Immunoprecipitation

100 mm of lentivirus infected HCT-116 cells were washed with 1× DPBS (Welgene, Daegu, Korea), pelleted, and crushed in immunoprecipitation (IP) buffer [150 mM NaCl, 25 mM HEPES-KOH (pH 7.5), 10% (v/v) glycerol, 1 mM MgCl_2_, 2 mM sodium orthovanadate, 2 mM β-glycerophosphate, 1 mM phenylmethylsulphonylfluoride (PMSF), 1 mM dithiothreitol (DTT), 2 mM ethylenediaminetetraacetic acid (EDTA), 0.5% Triton X-100, 1 × protease inhibitor cocktail (Roche)]. After brief homogenization and sonication, lysates were centrifuged at 16,000 × g for 5 min to remove insoluble materials and then incubated with anti-FLAG M2 affinity gel (Sigma-Aldrich) for 2 h at 4°C. The collected beads were then washed four to six times and boiled in SDS gel-loading buffer for WB analysis.

### Sodium dodecyl sulfate-polyacrylamide gel electrophoresis (SDS-PAGE) and in-gel tryptic digestion

The immunoprecipitates were run on an SDS-PAGE gel (NuPAGE® Novex 4–12% Bis-Tris gel; Invitrogen, Carlsbad, CA, USA) followed by staining with Colloidal Blue (Invitrogen). The SDS-PAGE gel was sliced into eight pieces for in-gel tryptic digestion using an in-gel tryptic digestion kit (Thermo Fisher Scientific, Rockford, IL, USA), according to the manufacturer's instructions. Briefly, the excised gels were destained, reduced using Tris [2-carboxyethyl] phosphine (TCEP) and alkylated using idoacetamide (IAA). The alkylated gel pieces were dehydrated in 100% acetonitrile (ACN) and digested with mass spectrometry (MS) grade trypsin in 25 mM NH_4_CO_3_ for 12 h at 30°C. The digested peptides were evaporated using a vacuum concentrator and cleaned using C18 spin columns (Thermo Fisher Scientific) for MS analysis.

### Liquid chromatography-mass spectrometry/mass spectrometry (LC-MS/MS) analysis and database search

The tryptic-digested peptides were analyzed using the Q Exactive^TM^ hybrid quadrupole-orbitrap mass spectrometer (Thermo Fisher Scientific) coupled with an Ultimate 3000 RSLCnano system (Thermo Fisher Scientific). The tryptic peptides were loaded onto a trap column (100 μm × 2 cm) packed with Acclaim PepMap100 C18 resin, from which the loaded peptides were eluted with a linear gradient of solvent B from 5–30% (0.1% formic acid in ACN) for 120 min at a flow rate of 300 nL/min. The eluted peptides separated by the analytical column (75 μm × 15 cm) were sprayed into a nano-electrospray ionization (ESI) source with an electrospray voltage of 2.4 kV. The Q Exactive Orbitrap mass analyzer was operated using a top 10 data-dependent method. Full MS scans were acquired over a m/z range of 300-2,000 with a mass resolution of 70,000 (at m/z 200). The automatic gain control (AGC) target value was 1.00E+06. The 10 most intense peaks with a charge state ≥ 2 were fragmented in the higher-energy collisional dissociation (HCD) collision cell with a normalized collision energy of 25%, and tandem mass spectra were acquired in the Orbitrap mass analyzer with a mass resolution of 17,500 at m/z 200.

Database searching of all raw data files was performed using Proteome Discoverer 1.4 software (Thermo Fisher Scientific). MASCOT 2.3.2 and SEQUEST were used for database searching against the Uniprot database. Database searching against the corresponding reversed database was also performed to evaluate the false discovery rate (FDR) of peptide identification. The database searching parameters included up to two missed cleavages for full tryptic digestion, a precursor ion mass tolerance of 10 ppm, a fragment ion mass tolerance of 0.02 Da, fixed modification for carbamidomethyl cysteine and variable modifications for methionine oxidation, and N/Q deamination. We obtained an FDR of less than 1% on the peptide level and filtered with high peptide confidence.

### Gene ontology analysis

Computational analysis was applied to all identified molecules that showed a unique interaction with calgranulin B compared with the control. Gene ontology (GO) information concerning biological processes, cellular components, and molecular functions was identified using DAVID ( http://david.abcc.ncifcrf.gov) [36, 37] and all significantly enriched (p<0.05) GO terms were described.

### Canonical pathway analysis

QIAGEN's Ingenuity® Pathway Analysis (IPA®, QIAGEN Redwood City, www.qiagen.com/ingenuity), which determines interactions and pathways of identified gene products from literature-based information, was used to identify the canonical pathways of the identified molecules. The significance of these pathways was determined using Fisher's exact test with a cut-off set at 0.05. The *p*-values from Fisher's exact test were adjusted for multiple testing with the Benjamini-Hochberf (B-H) multiple testing correction method. A negative log of these *p*-values [-log(*p*-value)] greater than 1.3, and a threshold value of 0.05, were set as the cutoffs for identifying canonical pathways.

### Protein network analysis and identification of upstream regulators

IPA was used to map the connection of all identified molecules, as well as their functions and involvement in diseases. IPA scans the set of input genes to identify networks using the Ingenuity Pathways Knowledge Base (IPKB) for interactions between identified molecules. In this study, all identified calgranulin B-interacting molecules and hypothetical interacting genes stored in IPKB were used to generate a set of networks. Networks were generated by IPA based on their connectivity, and each was ranked using a score and displayed graphically according to the genes/gene products and the biological relationships between the nodes. Upstream regulator analysis was also performed using IPA analysis (*p*-value <0.05) with all identified calgranulin B-interacting molecules.

### Protein-protein interaction by STRING analysis

All direct and indirect interactions between molecules that interacted with calgranulin B were analyzed using STRING, which is a database of known and predicted protein-protein interactions. The direct and indirect associations from computational prediction, and interactions from other databases for interaction analysis ( https://www.string-db.org) were included [38, 39]. Network analysis was set at a medium confidence (STRING score = 0.4). STRING score is calculated from the combination of all predictions with the range from 0 to 1 and classified into four categories: highest confidence (0.900), high confidence (0.700), medium confidence (0.400) and low confidence (0.150). Eight different colored lines were used to represent the types of evidence for associations, as follows: green, neighborhood evidence; red, gene fusion; blue, co-occurrence; black, co-expression; purple, experimental; light blue, database; yellow, text mining.; sky blue, protein homology.

## SUPPLEMENTARY MATERIALS FIGURES AND TABLES












