# Supplementary material for: Proteins that interact with calgranulin B in the human colon cancer cell line HCT-116
Source: Oncotarget. 2016 Dec 27;8(4):6819–32. doi: 10.18632/oncotarget.14301 (PMC5351672; doi:10.18632/oncotarget.14301)
Supplement: Supplementary file 1 [file oncotarget-08-6819-s001.pdf]

Proteins that interact with calgranulin B in the human colon cancer cell line HCT-116

SUPPLEMENTARY MATERIALS AND METHODS

Small interfering RNAs and transient transfection

Small interfering RNAs (siRNAs) were synthesized by Bioneer (Korea). siRNAs for human CD59 (siCD59; 5′-CAGUUCAGGUAUGUGUGUA-3′), FLOT1 (siFLOT1; 5′-GAGCAUGUCCAUUGACAGU-3′), DYNC1 (siDYNC1; 5′-CACAACAAGCCGCUCUACU-3′), and as a negative control, scrambled siRNA (siCon; 5′-GUUCAGCGUGUCCGGCGAG-3′) were used. Cells (2 × 10[6]) were plated on 100 mm culture dish 24h before transfection. Five hundred picomoles of siRNA and 25 μl of Lipofectamine™ 2000 (Life Technologies) were used for each transient transfection. After 48h, WB and proliferation assays were performed.

SUPPLEMENTARY FIGURE AND TABLES

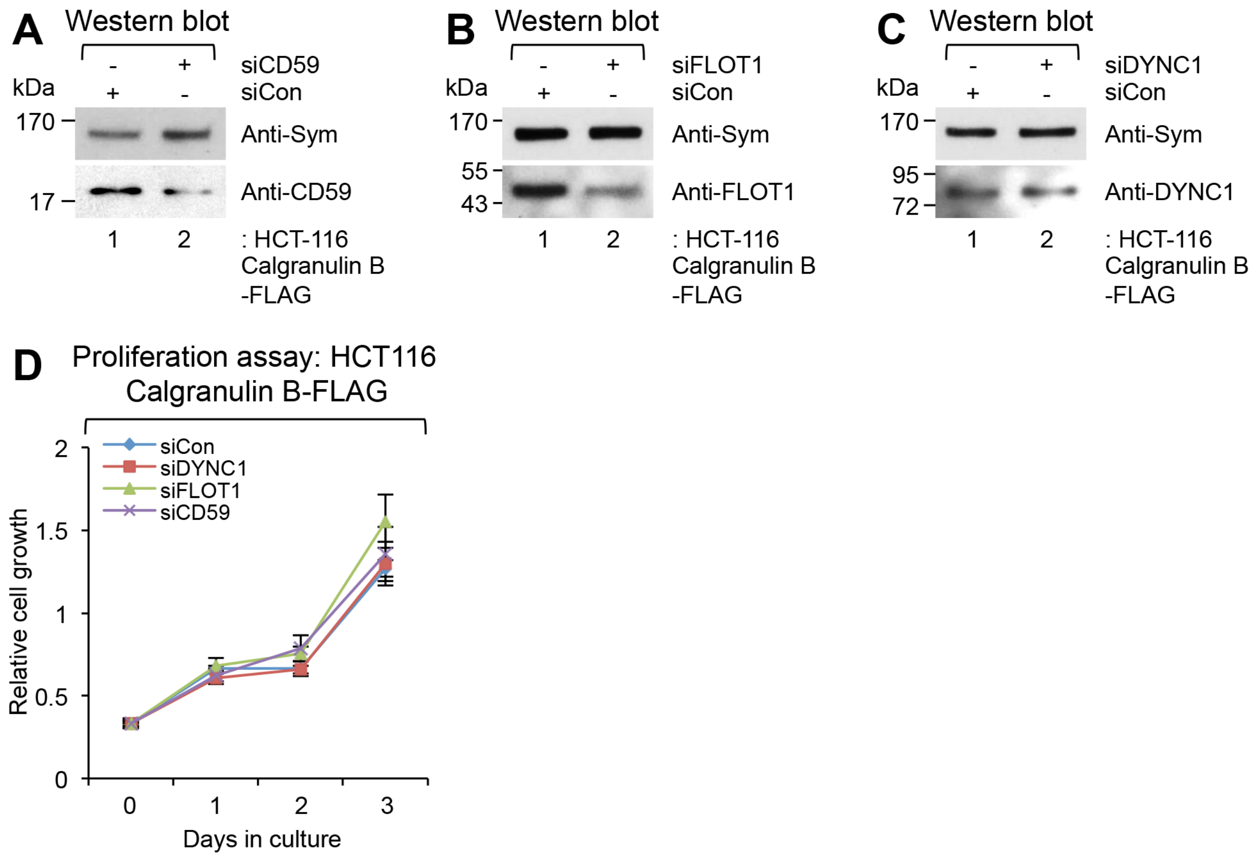

Supplementary Figure S1: Depletion of calgranulin B-interacting proteins do not affect proliferation of the colon cancer cell line HCT-116 which stably expressing calgranulin B. siRNAs for calgranulin B-interacting proteins CD59, FLOT1, DYNC1, and scrambled siRNA (siCon) were transfected into the colon cancer cell line HCT-116 which stably expressing calgranulin B-FLAG. WB **A-C.** and proliferation assay **D.** were performed. However, as shown in S1C panel anti-DYNC1, siRNA of DYNC1 did not show effective interfering in this cell. Data represent the mean values of at least three independent experiments performed in triplicate. Error bars in the graph represent ± SD.

Supplementary Table S1: Identification of proteins that interact with calgranulin B. Immunoprecipitates of calgranulin B were separated using sodium dodecyl sulfate-polyacrylamide gel electrophoresis (SDS-PAGE) as shown in Figure 2A and analyzed using liquid chromatography-mass spectrometry/mass spectrometry (LC-MS/MS) analysis as described in the MATERIALS AND METHODS. Candidate calgranulin B-interacting proteins are indicated in red and were used in bioinformatics analysis.

See Supplementary File 1

Supplementary Table S2: The list of gene ontology (GO) terms for biological processes, cellular components, and molecular functions of calgranulin B-interacting and associated proteins.

See Supplementary File 2

Supplementary Table S3: All identified canonical pathways of calgranulin B-interacting proteins determined using IPA.

See Supplementary File 3

Supplementary Table S4: Mechanistic networks for calgranulin B-interacting proteins including the molecules, top diseases, and function information in each network.

See Supplementary File 4

Supplementary Table S5: Upstream regulators and target molecules identified as calgranulin B-interacting proteins.

See Supplementary File 5
