# Supplementary material for: Proteins that interact with calgranulin B in the human colon cancer cell line HCT-116
Source: Oncotarget. 2016 Dec 27;8(4):6819–32. doi: 10.18632/oncotarget.14301 (PMC5351672; doi:10.18632/oncotarget.14301)
Supplement: Supplementary file 3 [file oncotarget-08-6819-s003.docx]

**Supplementary Table 2.** The list of gene ontology (GO) terms for biological processes, cellular components, and molecular functions of calgranulin B-interacting and associated proteins.

| **GO Term** | **GO ID** | **Count** | **%** | **PValue** | **Fold Enrichment** | **Bonferroni** | **Benjamini** | **FDR** | **Genes** |
| --- | --- | --- | --- | --- | --- | --- | --- | --- | --- |
| **BIOLOGICAL PROCESS** | | | | | | | | | |
| translational elongation | GO:0006414 | 36 | 8.13 | 1.24E-29 | 13.00 | 1.70E-26 | 1.70E-26 | 2.04E-26 | *RPL18, RPL17, RPL14, RPL13, RPL15, RPL35, RPS27, RPL30, RPL31, RPS3A, RPL6, RPL34, RPL3, RPL10, RPL5, RPL11, RPL10A, RPS23, RPL35A, RPL26, RPS9, EEF2, RPL23A, RPL24, RPS4X, RPS5, RPS8, RPS7, RPS18, RPS19, RPL18A, RPS16, RPL21, EEF1G, RPS11, EEF1D* |
| ribonucleoprotein complex  biogenesis | GO:0022613 | 42 | 9.48 | 1.74E-26 | 8.51 | 2.37E-23 | 1.19E-23 | 2.85E-23 | *EIF6, RPL14, GAR1, UTP18, SNRPD3, UTP15, BOP1, BMS1, SFRS6, EBNA1BP2, EIF3A, WDR36, RRP1B, SFRS9, BRIX1, WDR12, RPL5, RPL11, IMP4, FTSJ3, CLNS1A, KRR1, RPL35A, TBL3, PDCD11, BYSL, MPHOSPH10, SFRS13A, RPL26, RPL24, RRP9, SFRS1, SF3A1, RPS7, RCL1, NOP2, RPS19, RPS16, SNRNP200, NOP58, WDR3, PES1* |
| RNA processing | GO:0006396 | 67 | 15.12 | 3.67E-25 | 4.47 | 5.01E-22 | 1.67E-22 | 6.02E-22 | *RALY, RPL14, GAR1, UTP18, SNRPD3, UTP15, YBX1, PNN, SFRS6, SFRS7, WDR36, RRP1B, PCBP1, SRRM2, TARDBP, PCBP2, SFRS9, MAGOHB, RPL11, IMP4, FTSJ3, CLNS1A, KHDRBS1, KRR1, RPL35A, SNRPA1, EFTUD2, PTBP1, RRP9, SFRS1, SFRS3, RSL1D1, RPS19, NOP2, RPS16, SNRNP200, SNRPA, THOC4, SNRNP40, XRN2, BAT1, TRA2B, BOP1, SF3B3, PRPF19, PRPF8, HNRNPD, DHX15, WDR12, RPL5, RPL10A, DDX41, RBM25, PDCD11, TBL3, MPHOSPH10, SFRS13A, RPL26, SNW1, RNPS1, SF3A1, RPS7, HNRNPH3, NOP58, WDR3, PES1, ADAR* |
| ribosome biogenesis | GO:0042254 | 33 | 7.45 | 6.42E-23 | 9.86 | 8.77E-20 | 2.19E-20 | 1.05E-19 | *EIF6, RPL14, UTP18, GAR1, UTP15, BOP1, BMS1, EBNA1BP2, WDR36, RRP1B, BRIX1, WDR12, RPL5, RPL11, IMP4, FTSJ3, KRR1, RPL35A, TBL3, PDCD11, BYSL, RPL26, MPHOSPH10, RPL24, RRP9, RPS7, RCL1, NOP2, RPS19, RPS16, WDR3, NOP58, PES1* |
| translation | GO:0006412 | 48 | 10.84 | 5.66E-21 | 5.29 | 7.73E-18 | 1.55E-18 | 9.29E-18 | *EIF6, RPL18, RPL17, RPL14, RPL13, RPL15, RPL35, RPL22L1, RPL13AP3, EIF3C, IARS, EIF3D, MRPL10, RPL30, RPS27, EIF3A, RPL31, RPL6, RPS3A, EIF3E, RPL34, RPL3, RPL10, RPL5, RPL11, RPL10A, RPS23, RPL35A, RRBP1, RPL26, RPS9, EEF2, RPL23A, RPL24, RPS4X, RPS5, RPS8, RPS7, RSL1D1, RPS18, RPS19, RPL18A, RPS16, RPL21, EIF2S1, EEF1G, RPS11, EEF1D* |
| rRNA processing | GO:0006364 | 26 | 5.87 | 1.45E-18 | 10.30 | 1.98E-15 | 3.30E-16 | 2.38E-15 | *RPL14, UTP18, GAR1, UTP15, BOP1, WDR36, RRP1B, WDR12, RPL11, RPL5, IMP4, FTSJ3, RPL35A, KRR1, PDCD11, TBL3, RPL26, MPHOSPH10, RRP9, RPS7, RPS19, NOP2, RPS16, WDR3, NOP58, PES1* |
| rRNA metabolic process | GO:0016072 | 26 | 5.87 | 4.46E-18 | 9.88 | 6.10E-15 | 8.71E-16 | 7.33E-15 | *RPL14, UTP18, GAR1, UTP15, BOP1, WDR36, RRP1B, WDR12, RPL11, RPL5, IMP4, FTSJ3, RPL35A, KRR1, PDCD11, TBL3, RPL26, MPHOSPH10, RRP9, RPS7, RPS19, NOP2, RPS16, WDR3, NOP58, PES1* |
| nuclear mRNA splicing, via  spliceosome | GO:0000398 | 28 | 6.32 | 8.29E-15 | 6.67 | 1.14E-11 | 1.42E-12 | 1.37E-11 | *SNRPD3, TRA2B, SF3B3, YBX1, SFRS6, SFRS7, PRPF8, PCBP1, PCBP2, SFRS9, HNRNPD, RBM25, CLNS1A, SNRPA1, EFTUD2, PTBP1, SFRS13A, RNPS1, SNW1, SFRS1, SF3A1, SFRS3, HNRNPH3, SNRNP200, SNRPA, SNRNP40, THOC4, BAT1* |
| RNA splicing, via  transesterification reactions  with bulged adenosine as  nucleophile | GO:0000377 | 28 | 6.32 | 8.29E-15 | 6.67 | 1.14E-11 | 1.42E-12 | 1.37E-11 | *SNRPD3, TRA2B, SF3B3, YBX1, SFRS6, SFRS7, PRPF8, PCBP1, PCBP2, SFRS9, HNRNPD, RBM25, CLNS1A, SNRPA1, EFTUD2, PTBP1, SFRS13A, RNPS1, SNW1, SFRS1, SF3A1, SFRS3, HNRNPH3, SNRNP200, SNRPA, SNRNP40, THOC4, BAT1* |
| RNA splicing, via  transesterification reactions | GO:0000375 | 28 | 6.32 | 8.29E-15 | 6.67 | 1.14E-11 | 1.42E-12 | 1.37E-11 | *SNRPD3, TRA2B, SF3B3, YBX1, SFRS6, SFRS7, PRPF8, PCBP1, PCBP2, SFRS9, HNRNPD, RBM25, CLNS1A, SNRPA1, EFTUD2, PTBP1, SFRS13A, RNPS1, SNW1, SFRS1, SF3A1, SFRS3, HNRNPH3, SNRNP200, SNRPA, SNRNP40, THOC4, BAT1* |
| RNA splicing | GO:0008380 | 37 | 8.35 | 1.22E-14 | 4.75 | 1.67E-11 | 1.85E-12 | 2.01E-11 | *RALY, TRA2B, SNRPD3, YBX1, SF3B3, PNN, PRPF19, SFRS6, SFRS7, TARDBP, PRPF8, SRRM2, PCBP1, PCBP2, SFRS9, DHX15, HNRNPD, MAGOHB, DDX41, RBM25, CLNS1A, SNRPA1, EFTUD2, PTBP1, MPHOSPH10, SFRS13A, RNPS1, SNW1, SFRS1, SF3A1, SFRS3, HNRNPH3, SNRNP200, SNRPA, SNRNP40, THOC4, BAT1* |
| mRNA metabolic process | GO:0016071 | 42 | 9.48 | 1.73E-14 | 4.14 | 2.37E-11 | 2.37E-12 | 2.85E-11 | *RALY, TRA2B, SNRPD3, YBX1, SF3B3, PNN, PRPF19, SFRS6, SFRS7, EIF3E, PCBP1, TARDBP, PRPF8, SRRM2, PCBP2, SFRS9, DHX15, HNRNPD, MAGOHB, DDX41, RBM25, KHDRBS1, CLNS1A, SNRPA1, EFTUD2, PTBP1, SFRS13A, ELAVL1, RNPS1, SNW1, SFRS1, SF3A1, SFRS3, HNRNPH3, SERBP1, SNRNP200, SNRPA, SNRNP40, THOC4, XRN2, BAT1, ADAR* |
| mRNA processing | GO:0006397 | 39 | 8.80 | 2.02E-14 | 4.43 | 2.76E-11 | 2.51E-12 | 3.32E-11 | *RALY, TRA2B, SNRPD3, YBX1, SF3B3, PNN, PRPF19, SFRS6, SFRS7, TARDBP, PRPF8, SRRM2, PCBP1, PCBP2, SFRS9, DHX15, HNRNPD, MAGOHB, DDX41, RBM25, KHDRBS1, CLNS1A, SNRPA1, EFTUD2, PTBP1, SFRS13A, RNPS1, SNW1, SFRS1, SF3A1, SFRS3, HNRNPH3, SNRNP200, SNRPA, SNRNP40, THOC4, XRN2, BAT1, ADAR* |
| ncRNA processing | GO:0034470 | 26 | 5.87 | 4.96E-11 | 5.07 | 6.78E-08 | 5.65E-09 | 8.15E-08 | *RPL14, UTP18, GAR1, UTP15, BOP1, WDR36, RRP1B, WDR12, RPL11, RPL5, IMP4, FTSJ3, RPL35A, KRR1, PDCD11, TBL3, RPL26, MPHOSPH10, RRP9, RPS7, RPS19, NOP2, RPS16, WDR3, NOP58, PES1* |
| ncRNA metabolic process | GO:0034660 | 27 | 6.09 | 8.60E-10 | 4.28 | 1.18E-06 | 9.05E-08 | 1.41E-06 | *RPL14, UTP18, GAR1, UTP15, BOP1, IARS, WDR36, RRP1B, WDR12, RPL5, RPL11, IMP4, FTSJ3, RPL35A, KRR1, PDCD11, TBL3, RPL26, MPHOSPH10, RRP9, RPS7, NOP2, RPS19, RPS16, WDR3, NOP58, PES1* |
| ribosomal large subunit  biogenesis | GO:0042273 | 8 | 1.81 | 1.21E-09 | 29.17 | 1.65E-06 | 1.18E-07 | 1.99E-06 | *RPL35A, RPL14, RPL26, WDR12, RPL5, RPL11, RPL24, PES1* |
| actin cytoskeleton organization | GO:0030036 | 22 | 4.97 | 1.04E-06 | 3.55 | 0.001424 | 9.50E-05 | 0.001713 | *INF2, HRAS, SSH1, PDLIM7, EPB41, CALD1, FSCN1, S100A9, ARPC4, ARPC5, ITGB1, ARHGEF11, EPB41L2, ARPC1A, KRAS, EPS8, DYNLL1, INPP5K, CFL1, AMOT, ADD1, DLG1* |
| ribonucleoprotein complex  assembly | GO:0022618 | 12 | 2.71 | 2.43E-06 | 6.34 | 0.003316 | 2.08E-04 | 0.003991 | *EIF6, SFRS6, CLNS1A, EIF3A, SNRPD3, SNRNP200, SFRS9, SFRS13A, RPL24, SFRS1, BMS1, SF3A1* |
| oxidative phosphorylation | GO:0006119 | 14 | 3.16 | 2.55E-06 | 5.21 | 0.003482 | 2.05E-04 | 0.004192 | *UQCRC2, UQCRC1, NDUFA9, ATP5B, NDUFA10, NDUFV3, ATP6V1A, COX2, NDUFV1, ATP6V0A1, ATP5O, NDUFS3, NDUFS2, NDUFS1* |
| actin filament-based process | GO:0030029 | 22 | 4.97 | 2.93E-06 | 3.33 | 0.004003 | 2.23E-04 | 0.004821 | *INF2, HRAS, SSH1, PDLIM7, EPB41, CALD1, FSCN1, S100A9, ARPC4, ARPC5, ITGB1, ARHGEF11, EPB41L2, ARPC1A, KRAS, EPS8, DYNLL1, INPP5K, CFL1, AMOT, ADD1, DLG1* |
| generation of precursor  metabolites and energy | GO:0006091 | 25 | 5.64 | 5.53E-06 | 2.91 | 0.007536 | 3.98E-04 | 0.00909 | *UQCRC2, UQCRC1, NDUFA9, ATP5B, CYC1, COX4I1, HK1, NDUFA10, UQCRFS1, NDUFA12, COX6C, NDUFV3, ACADVL, ATP6V1A, SLC25A13, COX2, NDUFV1, PKM2, ATP6V0A1, ATP5O, NDUFS3, GAPDH, NDUFS2, NDUFS1, ENO1* |
| electron transport chain | GO:0022900 | 14 | 3.16 | 1.39E-05 | 4.48 | 0.018785 | 9.48E-04 | 0.022788 | *UQCRC2, UQCRC1, NDUFA9, CYC1, UQCRFS1, NDUFA10, NDUFA12, NDUFV3, SLC25A13, COX2, NDUFV1, NDUFS3, NDUFS2, NDUFS1* |
| cellular macromolecular  complex subunit organization | GO:0034621 | 26 | 5.87 | 1.68E-05 | 2.66 | 0.02273 | 0.001094 | 0.027629 | *EIF6, NUP98, SNRPD3, ARPC4, BMS1, SFRS6, TFAM, EIF3A, SET, PICALM, SFRS9, H2AFY, HSPA4, KPNB1, CLNS1A, NUP133, TCP1, SFRS13A, RPL24, SFRS1, SF3A1, HMGA1, SNRNP200, SMARCA5, TOMM22, XRN2* |
| protein folding | GO:0006457 | 17 | 3.84 | 2.85E-05 | 3.50 | 0.038221 | 0.00177 | 0.046824 | *TCP1, DNAJC10, CCT6A, CCT3, TRAP1, CCT5, HSP90B1, CCT4, SLMAP, BAG3, CCT8, HSPE1, RUVBL2, DNAJB1, RANBP2, UGGT1, DNAJB6* |
| respiratory electron transport  chain | GO:0022904 | 10 | 2.26 | 5.67E-05 | 5.70 | 0.074549 | 0.003363 | 0.093066 | *NDUFV3, SLC25A13, UQCRC1, COX2, NDUFA9, NDUFV1, NDUFS3, NDUFA10, NDUFS2, NDUFS1* |
| cellular macromolecular  complex assembly | GO:0034622 | 23 | 5.19 | 6.45E-05 | 2.64 | 0.084399 | 0.003667 | 0.105913 | *CLNS1A, EIF6, NUP98, TCP1, SNRPD3, SFRS13A, RPL24, ARPC4, SFRS1, SF3A1, BMS1, SFRS6, TFAM, EIF3A, PICALM, SET, SFRS9, SNRNP200, SMARCA5, H2AFY, HSPA4, TOMM22, KPNB1* |
| cytoskeleton organization | GO:0007010 | 28 | 6.32 | 6.70E-05 | 2.34 | 0.087504 | 0.003656 | 0.109991 | *HRAS, SSH1, PDLIM7, CALD1, S100A9, ARPC4, ARPC5, RCC1, ITGB1, KRAS, INPP5K, DYNLL1, DLG1, INF2, PLD2, EPB41, FSCN1, PCM1, ARHGEF11, EPB41L2, ARPC1A, ERBB2IP, EPS8, CFL1, AMOT, MAP7, DNAJB6, ADD1* |
| ATP synthesis coupled electron transport | GO:0042773 | 9 | 2.03 | 1.31E-04 | 5.86 | 0.163857 | 0.006859 | 0.214839 | *NDUFV3, UQCRC1, COX2, NDUFA9, NDUFV1, NDUFS3, NDUFA10, NDUFS2, NDUFS1* |
| mitochondrial ATP synthesis  coupled electron transport | GO:0042775 | 9 | 2.03 | 1.31E-04 | 5.86 | 0.163857 | 0.006859 | 0.214839 | *NDUFV3, UQCRC1, COX2, NDUFA9, NDUFV1, NDUFS3, NDUFA10, NDUFS2, NDUFS1* |
| cellular respiration | GO:0045333 | 11 | 2.48 | 3.15E-04 | 4.14 | 0.349721 | 0.015813 | 0.515868 | *UQCRC2, NDUFV3, SLC25A13, UQCRC1, COX2, NDUFA9, NDUFV1, NDUFS3, NDUFA10, NDUFS2, NDUFS1* |
| mRNA splice site selection | GO:0006376 | 5 | 1.13 | 4.43E-04 | 13.02 | 0.454582 | 0.021417 | 0.725892 | *SFRS6, SFRS9, SFRS13A, SFRS1, SF3A1* |
| mRNA transport | GO:0051028 | 10 | 2.26 | 6.08E-04 | 4.19 | 0.564627 | 0.028267 | 0.99439 | *NUP133, NUP98, NUP210, FMR1, SFRS13A, NUP93, MAGOHB, THOC4, RANBP2, BAT1* |
| mitochondrial electron  transport, NADH to ubiquinone | GO:0006120 | 7 | 1.58 | 9.17E-04 | 6.08 | 0.714674 | 0.040942 | 1.495914 | *NDUFV3, NDUFA9, NDUFV1, NDUFS3, NDUFA10, NDUFS2, NDUFS1* |
| **CELLULAR COMPONENT** | | | | | | | | | |
| ribonucleoprotein complex | GO:0030529 | 87 | 19.64 | 4.95E-41 | 5.62 | 1.98E-38 | 1.98E-38 | 6.91E-38 | *RALY, RPL18, RPL17, RPL14, RPL13, UTP18, GAR1, SNRPD3, RPL15, RPL22L1, YBX1, PNN, WDR36, RRP1B, RPS3A, PCBP1, SRRM2, SND1, PCBP2, RPL10, RPL11, IMP4, SNRPA1, RPL35A, KRR1, EFTUD2, PTBP1, FMR1, EEF2, RRP9, SFRS1, RPS4X, RSL1D1, RPS18, RPS19, RPS16, EIF2S1, SNRNP200, SNRPA, THOC4, SNRNP40, RPS11, BAT1, RPL35, BOP1, GCN1L1, RPL13AP3, SF3B3, PRPF19, MRPL10, RPL30, RPS27, RPL31, RPL6, RPL34, PRPF8, RPL3, HNRNPD, DHX15, WDR12, RPL5, RPL10A, DDX41, RBM25, RPS23, HNRNPAB, TBL3, RRBP1, RPL26, MPHOSPH10, RPS9, SNW1, RPL24, HBA2, RPL23A, ILF3, RPS5, SF3A1, RPS8, RPS7, HNRNPH3, RPL18A, RPL21, NOP58, WDR3, UTP20, PES1* |
| non-membrane-bounded  organelle | GO:0043228 | 176 | 39.73 | 4.07E-30 | 2.26 | 1.63E-27 | 8.13E-28 | 5.67E-27 | *EIF6, RPL18, HMGN1, RPL17, HRNR, RPL14, PDLIM7, VAPA, RPL13, SNRPD3, XRCC6, S100A9, RPL15, CBX3, RPL22L1, CCT3, PNN, WDR75, EBNA1BP2, CSNK2A1, MAK16, TARDBP, LRRC59, RPL10, H2AFY, DDX21, RPL11, ACIN1, DHX30, IMP4, GNL3, CLNS1A, NUP133, KRR1, RPL35A, TWF1, TWF2, FAM82A2, BYSL, FMR1, PTBP1, RRP9, PCM1, RSL1D1, ARPC1A, RCL1, RPS18, RPS19, RFC4, NOP2, RPS16, SMARCA5, CSTB, HSPB1, RPS11, MYBBP1A, ADD3, XRN2, ADD1, SSH1, IGF2BP2, ARPC4, BOP1, ARPC5, RCC1, RPL13AP3, COIL, RPS27, DDX47, PROCR, RPL6, EIF3E, BRIX1, HNRNPD, RPL3, RPL5, NAT10, RPL10A, RPS23, TBL3, EPB41, MKI67, FSCN1, AKAP8L, MPHOSPH10, RPS9, SNW1, RPL23A, HBA2, RPS5, RPS8, RPS7, CORO1C, TNKS1BP1, POLRMT, CCT5, UACA, RPL18A, EPS8, VCP, PCNA, WDR3, SEC13, MAP7, DNAJB1, TMPO, UTP20, ACAD11, ADAR, LZTS2, SPIN1, GAR1, UTP18, ATP5B, LYAR, UTP15, DMAP1, DYNLL1, RRP1B, RPS3A, SLMAP, TOP2B, TOP2A, FTSJ3, DLG1, RBBP4, SSBP1, FLOT1, RPS4X, HMGA1, NOC2L, ARHGEF11, EPB41L2, ACADVL, ARPC5L, CFL1, NOL10, NOL11, WDR43, NUP98, MTDH, CALD1, RPL35, CDH1, GCN1L1, BMS1, MRPL10, TFAM, KIAA0020, RPL30, NUMA1, RPL31, SAFB, PLEKHG6, RPL34, WDR12, AFAP1, CHD4, SYNPO, PLEC, PDCD11, TCP1, RRP12, RRBP1, RPL26, ILF3, RPL24, SF3A1, PWP2, DBT, UBTF, RPL21, AMOT, NOP58, CALM3, PES1* |
| intracellular  non-membrane-bounded  organelle | GO:0043232 | 176 | 39.73 | 4.07E-30 | 2.26 | 1.63E-27 | 8.13E-28 | 5.67E-27 | *EIF6, RPL18, HMGN1, RPL17, HRNR, RPL14, PDLIM7, VAPA, RPL13, SNRPD3, XRCC6, S100A9, RPL15, CBX3, RPL22L1, CCT3, PNN, WDR75, EBNA1BP2, CSNK2A1, MAK16, TARDBP, LRRC59, RPL10, H2AFY, DDX21, RPL11, ACIN1, DHX30, IMP4, GNL3, CLNS1A, NUP133, KRR1, RPL35A, TWF1, TWF2, FAM82A2, BYSL, FMR1, PTBP1, RRP9, PCM1, RSL1D1, ARPC1A, RCL1, RPS18, RPS19, RFC4, NOP2, RPS16, SMARCA5, CSTB, HSPB1, RPS11, MYBBP1A, ADD3, XRN2, ADD1, SSH1, IGF2BP2, ARPC4, BOP1, ARPC5, RCC1, RPL13AP3, COIL, RPS27, DDX47, PROCR, RPL6, EIF3E, BRIX1, HNRNPD, RPL3, RPL5, NAT10, RPL10A, RPS23, TBL3, EPB41, MKI67, FSCN1, AKAP8L, MPHOSPH10, RPS9, SNW1, RPL23A, HBA2, RPS5, RPS8, RPS7, CORO1C, TNKS1BP1, POLRMT, CCT5, UACA, RPL18A, EPS8, VCP, PCNA, WDR3, SEC13, MAP7, DNAJB1, TMPO, UTP20, ACAD11, ADAR, LZTS2, SPIN1, GAR1, UTP18, ATP5B, LYAR, UTP15, DMAP1, DYNLL1, RRP1B, RPS3A, SLMAP, TOP2B, TOP2A, FTSJ3, DLG1, RBBP4, SSBP1, FLOT1, RPS4X, HMGA1, NOC2L, ARHGEF11, EPB41L2, ACADVL, ARPC5L, CFL1, NOL10, NOL11, WDR43, NUP98, MTDH, CALD1, RPL35, CDH1, GCN1L1, BMS1, MRPL10, TFAM, KIAA0020, RPL30, NUMA1, RPL31, SAFB, PLEKHG6, RPL34, WDR12, AFAP1, CHD4, SYNPO, PLEC, PDCD11, TCP1, RRP12, RRBP1, RPL26, ILF3, RPL24, SF3A1, PWP2, DBT, UBTF, RPL21, AMOT, NOP58, CALM3, PES1* |
| cytosolic part | GO:0044445 | 40 | 9.03 | 7.13E-26 | 8.76 | 2.85E-23 | 9.51E-24 | 9.95E-23 | *RPL18, CYB5R3, RPL14, RPL35, GIPC1, CCT3, RPL30, RPS27, RPL31, RPL6, RPS3A, SLMAP, RPL34, RPL3, RPL10, RPL5, RPL11, HBB, RPS23, ENO1, TCP1, RPL26, RPS9, HBA2, RPL23A, RPL24, CCT6A, RPS4X, RPS5, RPS8, RPS7, RPS18, CCT5, CCT4, UACA, RPS19, RPL18A, RPS16, RPL21, RPS11* |
| cytosolic ribosome | GO:0022626 | 30 | 6.77 | 4.32E-24 | 12.33 | 1.73E-21 | 4.32E-22 | 6.03E-21 | *RPL18, RPL14, RPL13, RPL35, RPS27, RPL30, RPL31, RPS3A, RPL6, RPL34, RPL3, RPL10, RPL5, RPL11, RPS23, RPL26, RPS9, RPL24, RPL23A, HBA2, RPS4X, RPS5, RPS8, RPS7, RPS18, RPS19, RPS16, RPL18A, RPL21, RPS11* |
| intracellular organelle lumen | GO:0070013 | 127 | 28.67 | 3.97E-22 | 2.38 | 1.59E-19 | 3.17E-20 | 5.53E-19 | *EIF6, STK38, SNRPD3, S100A9, XRCC6, PNN, EBNA1BP2, WDR75, CSNK2A1, MAK16, SRRM2, TARDBP, LRRC59, RPL11, DDX21, ACIN1, DHX30, IMP4, GNL3, KRR1, BYSL, FMR1, PTBP1, LIG3, RRP9, SFRS1, RSL1D1, RCL1, RPS19, RFC4, NOP2, SMARCA5, CSTB, MYBBP1A, SMARCA2, UGGT1, XRN2, BAT1, BOP1, COIL, CORO2A, DDX47, EIF3E, BRIX1, RPL3, NAT10, RPL5, HSPE1, SDF4, TBL3, MKI67, PHB, AKAP8L, MPHOSPH10, RPS9, SNW1, RNPS1, RPS7, POLRMT, CCT5, VCP, PCNA, WDR3, DNAJB1, UTP20, ADAR, UTP18, GAR1, ATP5B, LYAR, DNAJC10, UTP15, DMAP1, RRP1B, RPS3A, TOP2B, KPNB1, TOP2A, FTSJ3, RBBP4, SSBP1, AIFM1, DDB1, NDUFA10, HMGA1, MCM5, ARHGEF11, NOC2L, ACADVL, C1QBP, CFL1, NOL10, NOL11, THOC4, RUVBL2, WDR43, MATR3, NUP98, MTDH, RPL35, CDC73, BMS1, PRPF19, TFAM, MRPL10, KIAA0020, NUMA1, SET, PRPF8, SAFB, WDR12, RBM25, CHD4, PDCD11, RRP12, NDUFA9, SFRS13A, ILF3, SF3A1, PWP2, DBT, HSP90B1, SON, UBTF, CALM3, NOP58, PES1* |
| membrane-enclosed lumen | GO:0031974 | 129 | 29.12 | 1.72E-21 | 2.31 | 6.86E-19 | 1.14E-19 | 2.39E-18 | *EIF6, STK38, SNRPD3, S100A9, XRCC6, PNN, EBNA1BP2, WDR75, CSNK2A1, MAK16, SRRM2, TARDBP, CPOX, LRRC59, RPL11, DDX21, ACIN1, DHX30, IMP4, GNL3, KRR1, BYSL, FMR1, PTBP1, LIG3, RRP9, SFRS1, RSL1D1, RCL1, RPS19, RFC4, NOP2, SMARCA5, CSTB, MYBBP1A, SMARCA2, UGGT1, XRN2, BAT1, BOP1, COIL, CORO2A, DDX47, EIF3E, BRIX1, RPL3, NAT10, RPL5, HSPE1, SDF4, TBL3, MKI67, PHB, AKAP8L, MPHOSPH10, RPS9, SNW1, RNPS1, RPS7, POLRMT, CCT5, VCP, PCNA, WDR3, DNAJB1, UTP20, ADAR, UTP18, GAR1, ATP5B, LYAR, DNAJC10, UTP15, DMAP1, RRP1B, RPS3A, TOP2B, KPNB1, TOP2A, NDUFS1, FTSJ3, RBBP4, SSBP1, AIFM1, DDB1, NDUFA10, HMGA1, MCM5, ARHGEF11, NOC2L, ACADVL, C1QBP, CFL1, NOL10, NOL11, THOC4, RUVBL2, WDR43, MATR3, NUP98, MTDH, RPL35, CDC73, BMS1, PRPF19, TFAM, MRPL10, KIAA0020, NUMA1, SET, PRPF8, SAFB, WDR12, RBM25, CHD4, PDCD11, RRP12, NDUFA9, SFRS13A, ILF3, SF3A1, PWP2, DBT, HSP90B1, SON, UBTF, CALM3, NOP58, PES1* |
| organelle lumen | GO:0043233 | 127 | 28.67 | 2.95E-21 | 2.32 | 1.18E-18 | 1.68E-19 | 4.11E-18 | *EIF6, STK38, SNRPD3, S100A9, XRCC6, PNN, EBNA1BP2, WDR75, CSNK2A1, MAK16, SRRM2, TARDBP, LRRC59, RPL11, DDX21, ACIN1, DHX30, IMP4, GNL3, KRR1, BYSL, FMR1, PTBP1, LIG3, RRP9, SFRS1, RSL1D1, RCL1, RPS19, RFC4, NOP2, SMARCA5, CSTB, MYBBP1A, SMARCA2, UGGT1, XRN2, BAT1, BOP1, COIL, CORO2A, DDX47, EIF3E, BRIX1, RPL3, NAT10, RPL5, HSPE1, SDF4, TBL3, MKI67, PHB, AKAP8L, MPHOSPH10, RPS9, SNW1, RNPS1, RPS7, POLRMT, CCT5, VCP, PCNA, WDR3, DNAJB1, UTP20, ADAR, UTP18, GAR1, ATP5B, LYAR, DNAJC10, UTP15, DMAP1, RRP1B, RPS3A, TOP2B, KPNB1, TOP2A, FTSJ3, RBBP4, SSBP1, AIFM1, DDB1, NDUFA10, HMGA1, MCM5, ARHGEF11, NOC2L, ACADVL, C1QBP, CFL1, NOL10, NOL11, THOC4, RUVBL2, WDR43, MATR3, NUP98, MTDH, RPL35, CDC73, BMS1, PRPF19, TFAM, MRPL10, KIAA0020, NUMA1, SET, PRPF8, SAFB, WDR12, RBM25, CHD4, PDCD11, RRP12, NDUFA9, SFRS13A, ILF3, SF3A1, PWP2, DBT, HSP90B1, SON, UBTF, CALM3, NOP58, PES1* |
| nucleolus | GO:0005730 | 73 | 16.48 | 5.24E-21 | 3.48 | 2.09E-18 | 2.62E-19 | 7.30E-18 | *EIF6, UTP18, GAR1, LYAR, SNRPD3, S100A9, UTP15, PNN, WDR75, EBNA1BP2, MAK16, RRP1B, RPS3A, TARDBP, DDX21, RPL11, ACIN1, TOP2B, TOP2A, IMP4, FTSJ3, GNL3, KRR1, BYSL, PTBP1, FMR1, RRP9, NOC2L, ARHGEF11, RSL1D1, RCL1, RPS19, NOP2, CSTB, NOL10, SMARCA5, NOL11, MYBBP1A, WDR43, XRN2, MTDH, RPL35, BOP1, COIL, BMS1, KIAA0020, DDX47, SAFB, BRIX1, RPL3, WDR12, RPL5, NAT10, PDCD11, TBL3, RRP12, MKI67, MPHOSPH10, RPS9, SNW1, ILF3, SF3A1, PWP2, RPS7, CCT5, UBTF, VCP, NOP58, WDR3, DNAJB1, UTP20, PES1, ADAR* |
| ribosome | GO:0005840 | 41 | 9.26 | 6.16E-21 | 6.35 | 2.46E-18 | 2.74E-19 | 8.59E-18 | *RPL18, RPL17, RPL14, RPL13, RPL15, RPL35, RPL22L1, RPL13AP3, GCN1L1, MRPL10, RPL30, RPS27, RPL31, RPL6, RPS3A, RPL34, RPL3, RPL10, RPL5, RPL11, RPL10A, RPS23, RPL35A, RRBP1, FMR1, RPL26, RPS9, RPL23A, HBA2, RPL24, RPS4X, RPS5, RPS8, RPS7, RSL1D1, RPS18, RPS19, RPL18A, RPS16, RPL21, RPS11* |
| nuclear lumen | GO:0031981 | 109 | 24.60 | 2.57E-20 | 2.50 | 1.03E-17 | 1.03E-18 | 3.58E-17 | *EIF6, STK38, SNRPD3, S100A9, XRCC6, PNN, EBNA1BP2, WDR75, CSNK2A1, MAK16, TARDBP, SRRM2, RPL11, DDX21, ACIN1, IMP4, GNL3, KRR1, BYSL, PTBP1, FMR1, LIG3, RRP9, SFRS1, RSL1D1, RCL1, RPS19, NOP2, RFC4, CSTB, SMARCA5, MYBBP1A, SMARCA2, XRN2, BAT1, BOP1, COIL, CORO2A, DDX47, EIF3E, BRIX1, RPL3, NAT10, RPL5, TBL3, MKI67, PHB, MPHOSPH10, AKAP8L, RPS9, SNW1, RNPS1, RPS7, CCT5, VCP, PCNA, WDR3, DNAJB1, UTP20, ADAR, UTP18, GAR1, LYAR, UTP15, DMAP1, RRP1B, RPS3A, TOP2B, TOP2A, KPNB1, FTSJ3, RBBP4, DDB1, HMGA1, MCM5, ARHGEF11, NOC2L, CFL1, NOL10, NOL11, THOC4, RUVBL2, WDR43, MATR3, NUP98, MTDH, RPL35, CDC73, BMS1, PRPF19, KIAA0020, NUMA1, SET, PRPF8, SAFB, WDR12, RBM25, CHD4, PDCD11, RRP12, SFRS13A, ILF3, SF3A1, PWP2, SON, UBTF, CALM3, NOP58, PES1* |
| somal subunit | GO:0033279 | 32 | 7.22 | 5.66E-20 | 8.32 | 2.26E-17 | 2.06E-18 | 7.89E-17 | *RPL18, RPL17, RPL14, RPL35, RPL13AP3, MRPL10, RPS27, RPL30, RPL31, RPS3A, RPL6, RPL34, RPL3, RPL10, RPL5, RPL11, RPS23, RPL26, RPS9, RPL23A, HBA2, RPL24, RPS4X, RPS5, RPS8, RPS7, RPS18, RPS19, RPS16, RPL18A, RPL21, RPS11* |
| cytosol | GO:0005829 | 96 | 21.67 | 2.63E-16 | 2.40 | 8.88E-14 | 7.44E-15 | 3.11E-13 | *CYB5R3, RPL18, RPL17, OCLN, RPL14, RPL13, CHMP4B, SNRPD3, RPL15, CCT3, SLC7A5, GSR, AGPAT5, RRP1B, DYNLL1, RPS3A, BAG3, SLMAP, PSMD2, RPL10, RPL11, ACIN1, RANBP2, TOP2B, KPNB1, IMPDH2, DLG1, CLNS1A, RPL35A, PPAP2C, CCT6A, KIDINS220, PCM1, RPS4X, HMGA1, ARHGEF11, ATP6V1A, RPS18, RPS19, RPS16, EIF2S1, PKM2, EEF1G, RPS11, EEF1D, ADD1, RPL35, HK1, GIPC1, IARS, EIF3C, EIF3D, EIF3A, RPS27, RPL30, NUMA1, SET, INPP5K, RPL31, RPL6, EIF3E, RPL34, RPL3, HNRNPD, RPL5, RPL10A, HBB, RPS23, PLEC, ENO1, AP2M1, OSBPL5, PDCD11, TCP1, RPL26, RPS9, RPL24, HBA2, RPL23A, RPS5, CAPRIN1, RPS8, RPS7, PSMD14, HSP90B1, CCT5, AP2A2, UACA, CCT4, CSNK1D, VCP, RPL18A, RPL21, CCT8, DSG1, CALM3* |
| cytosolic large ribosomal  subunit | GO:0022625 | 16 | 3.61 | 8.87E-14 | 14.02 | 3.55E-11 | 2.73E-12 | 1.24E-10 | *RPL18, RPL14, RPL35, RPL26, RPL24, RPL23A, RPL30, RPL18A, RPL6, RPL31, RPL34, RPL21, RPL3, RPL10, RPL5, RPL11* |
| large ribosomal subunit | GO:0015934 | 19 | 4.29 | 6.24E-13 | 9.44 | 2.49E-10 | 1.78E-11 | 8.70E-10 | *RPL18, RPL17, RPL14, RPL35, RPL26, RPL23A, RPL24, RPL13AP3, MRPL10, RPL30, RPL18A, RPL31, RPL6, RPL34, RPL21, RPL3, RPL10, RPL5, RPL11* |
| organelle envelope | GO:0031967 | 54 | 12.19 | 3.72E-12 | 2.90 | 1.49E-09 | 9.91E-11 | 5.18E-09 | *UQCRC2, EIF6, CYB5R3, UQCRC1, ATP5B, CYC1, CBX3, UQCRFS1, COX5A, COX5B, CPOX, DNAJC11, ATP5O, RANBP2, NDUFS3, KPNB1, NDUFS2, NDUFS1, NUP133, AIFM1, COX4I1, NDUFA10, NDUFA12, COX6C, ACADVL, LETM1, TOMM20, TOMM22, MATR3, APOOL, NUP98, MTDH, MTX3, MTX2, MTX1, HK1, NUP93, MTCH2, NUP210, MTCH1, NDUFA9, PHB, SUN1, NDUFV3, SLC25A11, SLC25A13, UACA, TOMM70A, COX2, NDUFV1, PCNA, RHOT1, SEC13, TMPO* |
| envelope | GO:0031975 | 54 | 12.19 | 4.23E-12 | 2.89 | 1.69E-09 | 1.06E-10 | 5.90E-09 | *UQCRC2, EIF6, CYB5R3, UQCRC1, ATP5B, CYC1, CBX3, UQCRFS1, COX5A, COX5B, CPOX, DNAJC11, ATP5O, RANBP2, NDUFS3, KPNB1, NDUFS2, NDUFS1, NUP133, AIFM1, COX4I1, NDUFA10, NDUFA12, COX6C, ACADVL, LETM1, TOMM20, TOMM22, MATR3, APOOL, NUP98, MTDH, MTX3, MTX2, MTX1, HK1, NUP93, MTCH2, NUP210, MTCH1, NDUFA9, PHB, SUN1, NDUFV3, SLC25A11, SLC25A13, UACA, TOMM70A, COX2, NDUFV1, PCNA, RHOT1, SEC13, TMPO* |
| organelle inner membrane | GO:0019866 | 36 | 8.13 | 6.56E-11 | 3.64 | 2.63E-08 | 1.54E-09 | 9.16E-08 | *UQCRC2, EIF6, CYB5R3, APOOL, UQCRC1, ATP5B, CYC1, MTX1, CBX3, UQCRFS1, COX5A, COX5B, MTCH2, DNAJC11, MTCH1, ATP5O, NDUFS3, NDUFS2, NDUFS1, NDUFA9, PHB, COX4I1, SUN1, NDUFA10, NDUFA12, COX6C, NDUFV3, ACADVL, SLC25A11, LETM1, SLC25A13, COX2, NDUFV1, PCNA, TOMM22, MATR3* |
| mitochondrial part | GO:0044429 | 48 | 10.84 | 9.95E-10 | 2.69 | 3.98E-07 | 2.21E-08 | 1.39E-06 | *UQCRC2, CYB5R3, APOOL, UQCRC1, MTX3, ATP5B, MTX2, CYC1, MTX1, HK1, UQCRFS1, COX5A, COX5B, TFAM, MRPL10, MTCH2, CPOX, DNAJC11, LRRC59, MTCH1, ATP5O, HSPE1, NDUFS3, DHX30, NDUFS2, NDUFS1, SSBP1, AIFM1, NDUFA9, PHB, COX4I1, NDUFA10, NDUFA12, COX6C, NDUFV3, ACADVL, DBT, POLRMT, SLC25A11, LETM1, SLC25A13, C1QBP, TOMM70A, COX2, NDUFV1, RHOT1, TOMM20, TOMM22* |
| mitochondrial envelope | GO:0005740 | 39 | 8.80 | 1.03E-09 | 3.10 | 4.13E-07 | 2.18E-08 | 1.44E-06 | *UQCRC2, CYB5R3, APOOL, UQCRC1, MTX3, ATP5B, MTX2, CYC1, MTX1, HK1, UQCRFS1, COX5A, COX5B, MTCH2, CPOX, DNAJC11, MTCH1, ATP5O, NDUFS3, NDUFS2, NDUFS1, AIFM1, NDUFA9, PHB, COX4I1, NDUFA10, NDUFA12, COX6C, NDUFV3, ACADVL, SLC25A11, LETM1, SLC25A13, TOMM70A, COX2, NDUFV1, RHOT1, TOMM20, TOMM22* |
| cytosolic small ribosomal  subunit | GO:0022627 | 13 | 2.93 | 1.16E-09 | 10.82 | 4.62E-07 | 2.31E-08 | 1.61E-06 | *RPS18, RPS27, RPS19, RPS16, RPS3A, RPS9, HBA2, RPS11, RPS4X, RPS5, RPS8, RPS23, RPS7* |
| mitochondrial membrane | GO:0031966 | 37 | 8.35 | 2.39E-09 | 3.13 | 9.56E-07 | 4.55E-08 | 3.33E-06 | *UQCRC2, CYB5R3, APOOL, UQCRC1, MTX3, ATP5B, MTX2, CYC1, MTX1, HK1, UQCRFS1, COX5A, COX5B, MTCH2, DNAJC11, MTCH1, ATP5O, NDUFS3, NDUFS2, NDUFS1, NDUFA9, PHB, COX4I1, NDUFA10, NDUFA12, COX6C, NDUFV3, ACADVL, SLC25A11, LETM1, SLC25A13, TOMM70A, COX2, NDUFV1, RHOT1, TOMM20, TOMM22* |
| mitochondrial inner membrane | GO:0005743 | 31 | 7.00 | 1.10E-08 | 3.37 | 4.41E-06 | 2.00E-07 | 1.54E-05 | *UQCRC2, CYB5R3, APOOL, UQCRC1, ATP5B, MTX1, CYC1, UQCRFS1, COX5A, COX5B, MTCH2, DNAJC11, MTCH1, ATP5O, NDUFS3, NDUFS2, NDUFS1, NDUFA9, PHB, COX4I1, NDUFA10, NDUFA12, COX6C, ACADVL, NDUFV3, SLC25A11, LETM1, SLC25A13, COX2, NDUFV1, TOMM22* |
| spliceosome | GO:0005681 | 19 | 4.29 | 7.69E-08 | 4.79 | 3.07E-05 | 1.34E-06 | 1.07E-04 | *RALY, SNRPA1, EFTUD2, SNRPD3, SNW1, SFRS1, SF3A1, SF3B3, PNN, PRPF19, SRRM2, PRPF8, SNRNP200, DHX15, SNRPA, THOC4, SNRNP40, DDX41, BAT1* |
| small ribosomal subunit | GO:0015935 | 13 | 2.93 | 2.96E-07 | 6.87 | 1.18E-04 | 4.94E-06 | 4.13E-04 | *RPS18, RPS27, RPS19, RPS16, RPS3A, RPS9, HBA2, RPS11, RPS4X, RPS5, RPS8, RPS23, RPS7* |
| preribosome | GO:0030684 | 7 | 1.58 | 9.99E-07 | 17.92 | 4.00E-04 | 1.60E-05 | 0.001394 | *TBL3, WDR36, RRP1B, WDR12, WDR3, BOP1, PES1* |
| mitochondrion | GO:0005739 | 62 | 14.00 | 1.13E-06 | 1.90 | 4.50E-04 | 1.73E-05 | 0.001569 | *UQCRC2, CYB5R3, UQCRC1, ATP5B, CYC1, UQCRFS1, COX5A, COX5B, PRDX1, MTFR1, ACOT9, GSR, AGPAT5, DNAJC11, CPOX, LRRC59, ATP5O, NDUFS3, DHX30, NDUFS2, NDUFS1, BSG, SSBP1, FAM82A2, AIFM1, COX4I1, NDUFA10, NDUFA12, COX6C, ACADVL, TRAP1, ATP6V1A, PYCR1, LETM1, C1QBP, TOMM20, TOMM22, YME1L1, APOOL, GPRC5C, MTX3, MTX2, MTX1, HK1, MRPL10, TFAM, MTCH2, MTCH1, HSPE1, AP2M1, NDUFA9, PHB, ILF3, NDUFV3, DBT, POLRMT, SLC25A11, SLC25A13, TOMM70A, COX2, NDUFV1, RHOT1* |
| small nucleolar  ribonucleoprotein complex | GO:0005732 | 8 | 1.81 | 1.14E-06 | 13.31 | 4.54E-04 | 1.68E-05 | 0.001585 | *KRR1, GAR1, UTP18, MPHOSPH10, NOP58, SNRNP40, RRP9, UTP20* |
| respiratory chain | GO:0070469 | 13 | 2.93 | 2.10E-06 | 5.77 | 8.38E-04 | 3.00E-05 | 0.002925 | *UQCRC2, NDUFV3, UQCRC1, COX2, NDUFA9, NDUFV1, CYC1, NDUFS3, NDUFA10, UQCRFS1, NDUFS2, NDUFS1, NDUFA12* |
| cell-cell junction | GO:0005911 | 20 | 4.51 | 4.29E-06 | 3.50 | 0.001715 | 5.92E-05 | 0.005986 | *INADL, MTDH, OCLN, VAPA, LIN7C, CDH1, CDH3, AMOTL1, SCRIB, PNN, DSG4, ITGA6, DSG1, PKP4, AMOT, DLG5, TJP2, ADD3, SYNPO, DLG1* |
| mitochondrial membrane part | GO:0044455 | 16 | 3.61 | 4.83E-06 | 4.26 | 0.001931 | 6.44E-05 | 0.006741 | *UQCRC2, UQCRC1, NDUFA9, ATP5B, UQCRFS1, NDUFA10, NDUFV3, TOMM70A, NDUFV1, TOMM20, RHOT1, ATP5O, TOMM22, NDUFS3, NDUFS2, NDUFS1* |
| organelle membrane | GO:0031090 | 59 | 13.32 | 1.32E-05 | 1.79 | 0.005278 | 1.71E-04 | 0.018451 | *UQCRC2, EIF6, CYB5R3, CLTA, UQCRC1, VAPA, ATP5B, CYC1, CBX3, UQCRFS1, COX5A, COX5B, DNAJC11, RPN1, ATP5O, ASPH, NDUFS3, DDOST, NDUFS2, NDUFS1, DLG1, BSG, COX4I1, ERLIN1, NDUFA10, NDUFA12, COX6C, ACADVL, LETM1, TOMM20, TOMM22, MATR3, GLG1, APOOL, MTDH, MTX3, MTX2, MTX1, HK1, GIPC1, MTCH2, MTCH1, SEC61A1, AP2M1, NDUFA9, RRBP1, PHB, SUN1, NDUFV3, SLC25A11, HSP90B1, AP2A2, SLC25A13, TOMM70A, COX2, NDUFV1, RHOT1, PCNA, TMPO* |
| chaperonin-containing  T-complex | GO:0005832 | 5 | 1.13 | 2.59E-05 | 23.78 | 0.01029 | 3.23E-04 | 0.03606 | *CCT5, TCP1, CCT4, CCT3, CCT6A* |
| nucleoid | GO:0009295 | 8 | 1.81 | 2.90E-05 | 8.59 | 0.011524 | 3.51E-04 | 0.040411 | *ACADVL, TFAM, DBT, POLRMT, SSBP1, ATP5B, LRRC59, DHX30* |
| mitochondrial nucleoid | GO:0042645 | 8 | 1.81 | 2.90E-05 | 8.59 | 0.011524 | 3.51E-04 | 0.040411 | *ACADVL, TFAM, DBT, POLRMT, SSBP1, ATP5B, LRRC59, DHX30* |
| apical junction complex | GO:0043296 | 13 | 2.93 | 3.89E-05 | 4.37 | 0.015425 | 4.57E-04 | 0.054192 | *INADL, OCLN, MTDH, VAPA, LIN7C, AMOTL1, PNN, DSG4, DSG1, PKP4, AMOT, TJP2, SYNPO* |
| apicolateral plasma membrane | GO:0016327 | 13 | 2.93 | 5.24E-05 | 4.24 | 0.020741 | 5.99E-04 | 0.073059 | *INADL, OCLN, MTDH, VAPA, LIN7C, AMOTL1, PNN, DSG4, DSG1, PKP4, AMOT, TJP2, SYNPO* |
| small nuclear ribonucleoprotein  complex | GO:0030532 | 7 | 1.58 | 5.92E-05 | 9.71 | 0.023419 | 6.58E-04 | 0.082599 | *SNRPA1, PRPF8, SNRPD3, SNRNP200, SNRNP40, RRP9, SF3B3* |
| mitochondrial respiratory chain | GO:0005746 | 10 | 2.26 | 1.14E-04 | 5.20 | 0.044437 | 0.001228 | 0.158375 | *UQCRC2, NDUFV3, UQCRC1, NDUFA9, NDUFV1, NDUFS3, NDUFA10, UQCRFS1, NDUFS2, NDUFS1* |
| nucleoplasm | GO:0005654 | 47 | 10.61 | 1.59E-04 | 1.77 | 0.061585 | 0.001671 | 0.2214 | *NUP98, STK38, GAR1, SNRPD3, XRCC6, CDC73, BOP1, DMAP1, COIL, PNN, PRPF19, CORO2A, NUMA1, CSNK2A1, SET, EIF3E, PRPF8, SRRM2, WDR12, TOP2B, KPNB1, TOP2A, CHD4, RBM25, GNL3, RBBP4, DDB1, PHB, PTBP1, FMR1, LIG3, SFRS13A, RNPS1, SFRS1, HMGA1, MCM5, SON, RFC4, UBTF, SMARCA5, PCNA, CALM3, RUVBL2, THOC4, PES1, SMARCA2, BAT1* |
| anchoring junction | GO:0070161 | 15 | 3.39 | 6.72E-04 | 2.90 | 0.235642 | 0.006867 | 0.93265 | *CDH1, CDH3, ITGB1, MLF2, SCRIB, PNN, DSG4, ITGA6, DSG1, PKP4, STARD8, DLG5, AFAP1, TJP2, TES* |
| **MOLECULAR FUNCTION** | | | | | | | | | |
| RNA binding | GO:0003723 | 82 | 18.51 | 3.59E-29 | 4.18 | 1.75E-26 | 1.75E-26 | 5.15E-26 | *RALY, RPL18, RPL14, RPL13, GAR1, RPL15, YBX1, SFRS6, SFRS7, DDX18, RPS3A, PCBP1, RAVER1, SRRM2, TARDBP, PCBP2, SFRS9, MAGOHB, DDX21, RPL11, DDX10, DHX30, KHDRBS1, RPL35A, KRR1, SNRPA1, PTBP1, FMR1, RRP9, SFRS1, RPS4X, SFRS3, RSL1D1, RPS18, RPS19, NOP2, RPS16, EIF2S1, SERBP1, SNRPA, THOC4, RPS11, MATR3, BAT1, TRA2B, RPL35, IGF2BP2, KIAA0020, DDX47, RPL30, RPL31, RPL6, RPL34, PRPF8, SAFB, RPL3, HNRNPD, RPL5, RPL10A, DDX41, RBM25, HNRNPAB, PDCD11, SFRS13A, RPL26, ELAVL1, RPS9, RPL24, RNPS1, ILF3, RPL23A, RPS5, SF3A1, RPS7, HNRNPH3, HSP90B1, SON, RPL18A, POLDIP3, RPL21, NOP58, ADAR* |
| structural constituent of  ribosome | GO:0003735 | 37 | 8.35 | 1.79E-22 | 8.05 | 8.76E-20 | 4.38E-20 | 2.58E-19 | *RPL18, RPL17, RPL14, RPL13, RPL15, RPL35, RPL22L1, RPL13AP3, MRPL10, RPL30, RPS27, RPL31, RPL6, RPS3A, RPL34, RPL3, RPL10, RPL5, RPL11, RPL10A, RPS23, RPL35A, RPL26, RPS9, RPL23A, RPL24, RPS4X, RPS5, RPS8, RPS7, RSL1D1, RPS18, RPS19, RPL18A, RPS16, RPL21, RPS11* |
| structural molecule activity | GO:0005198 | 56 | 12.64 | 1.43E-14 | 3.23 | 6.99E-12 | 2.33E-12 | 2.06E-11 | *RPL18, RPL17, CLTA, OCLN, RPL14, VAPA, RPL13, RPL15, RPL22L1, PNN, RPS3A, RPL10, RPL11, ASPH, DLG5, RPL35A, RPS4X, RSL1D1, EPB41L2, RPS18, RPS19, RPS16, SPRR3, RPS11, ADD3, MATR3, ADD1, NUP98, RPL35, ARPC5, RPL13AP3, MRPL10, RPL30, RPS27, EIF3A, NUMA1, RPL31, RPL6, RPL34, RPL3, RPL5, RPL10A, RPS23, PLEC, EPB41, RPL26, RPS9, RPL23A, RPL24, RPS5, RPS8, RPS7, RPL18A, ERBB2IP, RPL21, MAP7* |
| unfolded protein binding | GO:0051082 | 16 | 3.61 | 5.14E-07 | 5.09 | 2.51E-04 | 6.28E-05 | 7.38E-04 | *TCP1, DNAJC10, SFRS13A, CCT6A, CCT3, TRAP1, CCT5, HSP90B1, CCT4, SLMAP, CCT8, TOMM20, HSPE1, RUVBL2, DNAJB1, UGGT1* |
| rRNA binding | GO:0019843 | 8 | 1.81 | 9.82E-06 | 10.09 | 0.00478 | 9.58E-04 | 0.014091 | *RPS18, GAR1, RPS9, RPL5, RPL11, RPL23A, RPS11, RPS4X* |
| purine NTP-dependent helicase activity | GO:0070035 | 13 | 2.93 | 1.38E-05 | 4.85 | 0.006729 | 0.001125 | 0.019854 | *XRCC6, DDX27, DDX47, DDX18, SNRNP200, DHX15, DDX21, RUVBL2, DDX10, DHX30, DDX41, CHD4, BAT1* |
| ATP-dependent helicase  activity | GO:0008026 | 13 | 2.93 | 1.38E-05 | 4.85 | 0.006729 | 0.001125 | 0.019854 | *XRCC6, DDX27, DDX47, DDX18, SNRNP200, DHX15, DDX21, RUVBL2, DDX10, DHX30, DDX41, CHD4, BAT1* |
| ATPase activity | GO:0016887 | 25 | 5.64 | 1.55E-05 | 2.74 | 0.007519 | 0.001078 | 0.022193 | *RBBP4, ATP5B, XRCC6, ATP1A1, DDX27, ATP6V1A, DDX47, RFC4, DDX18, VCP, CCT8, SNRNP200, DHX15, SMARCA5, RUVBL2, DDX21, ATP5O, DDX10, ACIN1, DDX41, DHX30, TOP2A, SMARCA2, CHD4, BAT1* |
| ATPase activity, coupled | GO:0042623 | 22 | 4.97 | 1.83E-05 | 2.96 | 0.008888 | 0.001115 | 0.026251 | *RBBP4, ATP5B, XRCC6, ATP1A1, DDX27, ATP6V1A, DDX47, DDX18, RFC4, CCT8, SNRNP200, DHX15, DDX21, ATP5O, RUVBL2, DDX10, DHX30, DDX41, SMARCA2, TOP2A, CHD4, BAT1* |
| helicase activity | GO:0004386 | 15 | 3.39 | 2.83E-05 | 3.92 | 0.013707 | 0.001532 | 0.040579 | *XRCC6, DDX27, DDX47, DDX18, SNRNP200, DHX15, SMARCA5, DDX21, RUVBL2, DDX10, DHX30, DDX41, SMARCA2, CHD4, BAT1* |
| nucleotide binding | GO:0000166 | 91 | 20.54 | 5.99E-05 | 1.48 | 0.028822 | 0.00292 | 0.08597 | *RALY, HRAS, STK38, ATP5B, XRCC6, PRKG2, CCT3, SFRS6, DDX27, GSR, SFRS7, DDX18, CSNK2A1, RAVER1, TARDBP, SFRS9, DDX21, ACIN1, DDX10, TOP2B, DHX30, NDUFS2, TOP2A, GNL3, TRPM4, TWF2, AIFM1, EFTUD2, PTBP1, LIG3, EEF2, CCT6A, SFRS1, NDUFA10, DAPK3, SFRS3, MCM5, ACADVL, TRAP1, ATP6V1A, RFC4, PKM2, SNRNP200, SMARCA5, SNRPA, YME1L1, THOC4, RUVBL2, CSNK1G3, SMARCA2, MATR3, BAT1, TRA2B, HK1, IGF2BP2, BMS1, IARS, DDX47, KRAS, SAFB, HNRNPD, DHX15, RAB11A, HSPA4, HSPE1, NAT10, DDX41, GAPDH, RBM25, CHD4, HNRNPAB, TCP1, MKI67, SFRS13A, ELAVL1, RNPS1, ATP1A1, RPL23A, ATAD1, EPHA2, HNRNPH3, HSP90B1, CCT5, CCT4, CSNK1D, VCP, POLDIP3, NDUFV1, CCT8, RHOT1, ACAD11* |
| mRNA binding | GO:0003729 | 10 | 2.26 | 7.07E-05 | 5.54 | 0.033913 | 0.003132 | 0.101411 | *SERBP1, FMR1, RPL35, HNRNPD, ELAVL1, RNPS1, IGF2BP2, RPS5, RBM25, HNRNPAB* |
| actin binding | GO:0003779 | 23 | 5.19 | 8.97E-05 | 2.58 | 0.042821 | 0.00364 | 0.128622 | *INF2, TWF1, TWF2, SSH1, EPB41, CLMN, CALD1, FSCN1, GIPC1, ARPC4, ARPC5, EPB41L2, C14ORF49, ARPC1A, CORO1C, CORO2A, ARPC5L, CFL1, AFAP1, ADD3, ADD1, SYNPO, PLEC* |
| monovalent inorganic cation  transmembrane transporter  activity | GO:0015077 | 12 | 2.71 | 1.23E-04 | 4.22 | 0.0581 | 0.004594 | 0.175872 | *ATP6V1A, UQCRC1, COX2, ATP5B, COX4I1, ATP6V0A1, ATP5O, ATP1A1, UQCRFS1, COX5A, COX5B, COX6C* |
| NADH dehydrogenase activity | GO:0003954 | 8 | 1.81 | 1.46E-04 | 6.80 | 0.068774 | 0.005077 | 0.209324 | *NDUFV3, NDUFA9, NDUFV1, NDUFS3, NDUFA10, NDUFS2, NDUFS1, NDUFA12* |
| NADH dehydrogenase  (quinone) activity | GO:0050136 | 8 | 1.81 | 1.46E-04 | 6.80 | 0.068774 | 0.005077 | 0.209324 | *NDUFV3, NDUFA9, NDUFV1, NDUFS3, NDUFA10, NDUFS2, NDUFS1, NDUFA12* |
| NADH dehydrogenase  (ubiquinone) activity | GO:0008137 | 8 | 1.81 | 1.46E-04 | 6.80 | 0.068774 | 0.005077 | 0.209324 | *NDUFV3, NDUFA9, NDUFV1, NDUFS3, NDUFA10, NDUFS2, NDUFS1, NDUFA12* |
| hydrogen ion transmembrane  transporter activity | GO:0015078 | 11 | 2.48 | 1.65E-04 | 4.47 | 0.077299 | 0.005349 | 0.236309 | *ATP6V1A, UQCRC1, COX2, ATP5B, COX4I1, ATP6V0A1, ATP5O, UQCRFS1, COX5A, COX5B, COX6C* |
| cytoskeletal protein binding | GO:0008092 | 29 | 6.55 | 2.88E-04 | 2.10 | 0.131262 | 0.008756 | 0.41296 | *SSH1, CALD1, ARPC4, GIPC1, ARPC5, C14ORF49, CORO2A, NUMA1, TARDBP, AFAP1, PLEC, DLG1, SYNPO, INF2, TWF1, TWF2, EPB41, CLMN, FSCN1, EPB41L2, CORO1C, ARPC1A, TNKS1BP1, ARPC5L, CFL1, CALM3, PRNP, ADD3, ADD1* |
| oxidoreductase activity, acting  on NADH or NADPH  quinone or similar compound  as acceptor | GO:0016655 | 8 | 1.81 | 3.38E-04 | 5.97 | 0.152206 | 0.009666 | 0.484406 | *NDUFV3, NDUFA9, NDUFV1, NDUFS3, NDUFA10, NDUFS2, NDUFS1, NDUFA12* |
| RNA splicing factor activity,  transesterification mechanism | GO:0031202 | 6 | 1.35 | 4.06E-04 | 9.14 | 0.179796 | 0.010951 | 0.581182 | *PRPF8, TRA2B, MPHOSPH10, SFRS13A, SNRNP40, SF3B3* |
| oxidoreductase activity, acting  on NADH or NADPH | GO:0016651 | 10 | 2.26 | 4.18E-04 | 4.41 | 0.184678 | 0.010688 | 0.598635 | *NDUFV3, CYB5R3, GSR, NDUFA9, NDUFV1, NDUFS3, NDUFA10, NDUFS2, NDUFS1, NDUFA12* |
