# Supplementary material for: Proteins that interact with calgranulin B in the human colon cancer cell line HCT-116
Source: Oncotarget. 2016 Dec 27;8(4):6819–32. doi: 10.18632/oncotarget.14301 (PMC5351672; doi:10.18632/oncotarget.14301)
Supplement: Supplementary file 4 [file oncotarget-08-6819-s004.docx]

**Supplementary Table 3.** All identified canonical pathways of calgranulin B-interacting proteins determined using IPA.

| **Ingenuity Canonical Pathways** | **-log**  **(p-value)** | **Ratio** | **-log(B-H**  **p-value)** | **Molecules** |
| --- | --- | --- | --- | --- |
| EIF2 Signaling | 2.84E01 | 2.22E-01 | 2.59E01 | *EIF2S1, RPS27, RPL35A, RPS5, RPL26, RPL6, RPS19, RPL5, EIF3E, RPS11, RPL3, RPL22L1, RPL31, RPS8, RPS3A, RPL14, RPL18A, RPL30, KRAS, RPL17, RPL11, RPS18, EIF3A, RPL21, RPL35, RPS7, RPL13, RPL23A, RPS16, EIF3D, RPL24, RPS9, RPL18, RPS23, RPL34, RPS4X, EIF3C, RPL15, HRAS, RPL10, RPL10A* |
| Oxidative Phosphorylation | 1.18E01 | 1.83E-01 | 9.59E00 | *NDUFA10, UQCRC1, COX4I1, COX5A, NDUFA12, NDUFV3, NDUFV1, COX5B, COX6C, NDUFS1, CYC1, UQCRC2, NDUFS2, ATP5O, NDUFS3, NDUFA9, MT-CO2, UQCRFS1, ATP5B* |
| Mitochondrial Dysfunction | 1.07E01 | 1.33E-01 | 8.68E00 | *NDUFA10, UQCRC1, COX4I1, COX5A, NDUFA12, NDUFV3, NDUFV1, COX5B, COX6C, NDUFS1, CYC1, CYB5R3, UQCRC2, NDUFS2, ATP5O, NDUFS3, GSR, NDUFA9, AIFM1, MT-CO2, UQCRFS1, ATP5B* |
| Regulation of eIF4 and p70S6K Signaling | 1.04E01 | 1.36E-01 | 8.53E00 | *EIF2S1, KRAS, RPS27, RPS18, ITGA3, EIF3A, RPS7, RPS5, RPS19, RPS16, EIF3D, EIF3E, RPS9, ITGB1, RPS23, RPS11, RPS8, RPS4X, EIF3C, RPS3A, HRAS* |
| mTOR Signaling | 7.78E00 | 1.03E-01 | 5.97E00 | *KRAS, RPS27, RPS18, EIF3A, RPS7, RPS5, RPS19, RPS16, EIF3D, EIF3E, RPS9, PLD2, RPS23, RPS11, RHOT1, RPS8, RPS4X, EIF3C, RPS3A, HRAS* |
| Actin Nucleation by ARP-WASP Complex | 5.42E00 | 1.61E-01 | 3.68E00 | *ARPC5, KRAS, ITGA3, ITGB1, ARPC4, RHOT1, ARPC5L, HRAS, ARPC1A* |
| Virus Entry via Endocytic Pathways | 3.33E00 | 8.82E-02 | 1.68E00 | *KRAS, CLTA, ITGA3, ITGB1, ITGA6, AP2A2, HLA-B, AP2M1, HRAS* |
| RhoGDI Signaling | 3.29E00 | 6.98E-02 | 1.68E00 | *ARPC5, ARHGEF11, CFL1, ITGA3, RACK1, ITGB1, ARPC4, RHOT1, CDH1, CDH3, ARPC5L, ARPC1A* |
| Regulation of Actin-based Motility by Rho | 3.13E00 | 9.2E-02 | 1.57E00 | *ARPC5, CFL1, ITGA3, ITGB1, ARPC4, RHOT1, ARPC5L, ARPC1A* |
| Rac Signaling | 2.92E00 | 7.76E-02 | 1.41E00 | *ARPC5, KRAS, CFL1, ITGA3, ITGB1, ARPC4, ARPC5L, HRAS, ARPC1A* |
| Clathrin-mediated Endocytosis Signaling | 2.8E00 | 6.12E-02 | 1.33E00 | *ARPC5, RAB11A, S100A8, PICALM, CLTA, ITGB1, ARPC4, AP2A2, AP2M1, ARPC5L, CSNK2A1, ARPC1A* |
| Ephrin Receptor Signaling | 2.76E00 | 6.4E-02 | 1.32E00 | *ARPC5, KRAS, CFL1, ITGA3, RACK1, ITGB1, ARPC4, ARPC5L, EPHA2, HRAS, ARPC1A* |
| HIPPO signaling | 2.53E00 | 8.24E-02 | 1.13E00 | *SCRIB, PATJ, CSNK1D, AMOT, DLG1, TJP2, DLG5* |
| RAN Signaling | 2.3E00 | 1.88E-01 | 9.35E-01 | *RCC1, RANBP2, KPNB1* |
| fMLP Signaling in Neutrophils | 2.25E00 | 6.67E-02 | 9.35E-01 | *ARPC5, KRAS, CALM1 (includes others), RACK1, ARPC4, ARPC5L, HRAS, ARPC1A* |
| Sertoli Cell-Sertoli Cell Junction Signaling | 2.25E00 | 5.78E-02 | 9.35E-01 | *KRAS, EPB41, OCLN, DLG1, ITGA3, ITGB1, PRKG2, TJP2, CDH1, HRAS* |
| Cdc42 Signaling | 2.07E00 | 6.2E-02 | 7.99E-01 | *ARPC5, CFL1, ITGA3, ITGB1, ARPC4, HLA-B, ARPC5L, ARPC1A* |
| 1D-myo-inositol Hexakisphosphate  Biosynthesis II (Mammalian) | 2.02E00 | 1.5E-01 | 7.99E-01 | *CALM1 (includes others), INPP5K, SEC16A* |
| D-myo-inositol (1, 3, 4) - trisphosphate  Biosynthesis | 2.02E00 | 1.5E-01 | 7.99E-01 | *CALM1 (includes others), INPP5K, SEC16A* |
| Signaling by Rho Family GTPases | 2.01E00 | 4.88E-02 | 7.99E-01 | *ARPC5, ARHGEF11, CFL1, ITGA3, RACK1, ITGB1, ARPC4, RHOT1, CDH1, CDH3, ARPC5L, ARPC1A* |
| NRF2-mediated Oxidative Stress Response | 1.97E00 | 5.26E-02 | 7.79E-01 | *KRAS, DNAJC10, DNAJB1, DNAJC11, GSR, DNAJB6, VCP, PRDX1, HRAS, MGST3* |
| Aldosterone Signaling in Epithelial Cells | 1.92E00 | 5.45E-02 | 7.44E-01 | *HSPB1, KRAS, HSPA4, DNAJC10, DNAJB1, DNAJC11, HSP90B1, DNAJB6, PLCD3* |
| Neuregulin Signaling | 1.9E00 | 6.98E-02 | 7.44E-01 | *KRAS, ERBIN, ITGA3, ITGB1, HSP90B1, HRAS* |
| Glycolysis I | 1.8E00 | 1.25E-01 | 6.78E-01 | *PKM, GAPDH, ENO1* |
| Remodeling of Epithelial Adherens Junctions | 1.8E00 | 7.58E-02 | 6.78E-01 | *ARPC5, ARPC4, CDH1, ARPC5L, ARPC1A* |
| Agrin Interactions at Neuromuscular Junction | 1.77E00 | 7.46E-02 | 6.75E-01 | *KRAS, ITGA3, ITGB1, ITGA6, HRAS* |
| Superpathway of D-myo-inositol (1, 4, 5)-  trisphosphate Metabolism | 1.75E00 | 1.2E-01 | 6.75E-01 | *CALM1 (includes others), INPP5K, SEC16A* |
| Fcγ Receptor-mediated Phagocytosis in  Macrophages and Monocytes | 1.74E00 | 6.45E-02 | 6.75E-01 | *ARPC5, RAB11A, ARPC4, PLD2, ARPC5L, ARPC1A* |
| PI3K/AKT Signaling | 1.7E00 | 5.74E-02 | 6.6E-01 | *KRAS, GDF15, ITGA3, ITGB1, HSP90B1, INPP5K, HRAS* |
| Glioma Invasiveness Signaling | 1.69E00 | 7.14E-02 | 6.6E-01 | *KRAS, VTN, RHOT1, HRAS, PLG* |
| Caveolar-mediated Endocytosis Signaling | 1.67E00 | 7.04E-02 | 6.6E-01 | *FLOT1, ITGA3, ITGB1, ITGA6, HLA-B* |
| Integrin Signaling | 1.67E00 | 4.72E-02 | 6.6E-01 | *ARPC5, KRAS, ITGA3, ITGB1, ARPC4, ITGA6, RHOT1, ARPC5L, HRAS, ARPC1A* |
| Systemic Lupus Erythematosus Signaling | 1.61E00 | 4.61E-02 | 6.12E-01 | *SNRNP200, KRAS, PRPF8, SNRPA1, SNRPA, EFTUD2, SNRPD3, HLA-B, HRAS, PRPF19* |
| Actin Cytoskeleton Signaling | 1.55E00 | 4.5E-02 | 5.87E-01 | *SSH1, ARPC5, KRAS, CFL1, ITGA3, ITGB1, ARPC4, ARPC5L, HRAS, ARPC1A* |
| Protein Ubiquitination Pathway | 1.55E00 | 4.33E-02 | 5.87E-01 | *HSPB1, USP46, HSPA4, DNAJC10, DNAJB1, DNAJC11, HSP90B1, PSMD14, HLA-B, DNAJB6, PSMD2* |
| BER pathway | 1.55E00 | 1.67E-01 | 5.87E-01 | *LIG3, PCNA* |
| Role of IL-17A in Psoriasis | 1.48E00 | 1.54E-01 | 5.33E-01 | *S100A8, S100A9* |
| Regulation of Cellular Mechanics by Calpain  Protease | 1.46E00 | 7.27E-02 | 5.27E-01 | *KRAS, ITGA3, ITGB1, HRAS* |
| Germ Cell-Sertoli Cell Junction Signaling | 1.44E00 | 4.73E-02 | 5.12E-01 | *KRAS, CFL1, ITGA3, ITGB1, ITGA6, RHOT1, CDH1, HRAS* |
| DNA Double-Strand Break Repair by Non-  Homologous End Joining | 1.42E00 | 1.43E-01 | 5.06E-01 | *LIG3, XRCC6* |
| Epithelial Adherens Junction Signaling | 1.38E00 | 4.9E-02 | 4.74E-01 | *ARPC5, KRAS, ARPC4, CDH1, ARPC5L, HRAS, ARPC1A* |
| NF-κB Activation by Viruses | 1.36E00 | 5.81E-02 | 4.72E-01 | *KRAS, ITGA3, ITGB1, ITGA6, HRAS* |
| Dolichol and Dolichyl PhosphateBiosynthesis | 1.35E00 | 5E-01 | 4.72E-01 | *DHDDS* |
| Mismatch Repair in Eukaryotes | 1.31E00 | 1.25E-01 | 4.4E-01 | *RFC4, PCNA* |
| Role of Tissue Factor in Cancer | 1.29E00 | 5.04E-02 | 4.39E-01 | *KRAS, CFL1, ITGA3, ITGB1, ITGA6, HRAS* |
| PTEN Signaling | 1.29E00 | 5.04E-02 | 4.39E-01 | *KRAS, ITGA3, ITGB1, INPP5K, HRAS, CSNK2A1* |
| RhoA Signaling | 1.28E00 | 5E-02 | 4.34E-01 | *ARPC5, ARHGEF11, CFL1, ARPC4, ARPC5L, ARPC1A* |
| Thyroid Cancer Signaling | 1.25E00 | 7.69E-02 | 4.16E-01 | *KRAS, CDH1, HRAS* |
| Glutathione Redox Reactions I | 1.22E00 | 1.11E-01 | 4.03E-01 | *GSR, MGST3* |
| D-myo-inositol (1, 4, 5) - trisphosphate  Degradation | 1.22E00 | 1.11E-01 | 4.03E-01 | *INPP5K, SEC16A* |
| Diphthamide Biosynthesis | 1.18E00 | 3.33E-01 | 3.98E-01 | *EEF2* |
| NADH Repair | 1.18E00 | 3.33E-01 | 3.98E-01 | *GAPDH* |
| Oxidized GTP and dGTP Detoxification | 1.18E00 | 3.33E-01 | 3.98E-01 | *RUVBL2* |
| Chemokine Signaling | 1.18E00 | 5.88E-02 | 3.98E-01 | *KRAS, CFL1, CALM1 (includes others), HRAS* |
| Triacylglycerol Biosynthesis | 1.17E00 | 7.14E-02 | 3.97E-01 | *AGPAT5, DBT, PLPP2* |
| CDK5 Signaling | 1.16E00 | 5.1E-02 | 3.94E-01 | *KRAS, ITGA3, ITGB1, ITGA6, HRAS* |
| DNA Methylation and Transcriptional  Repression Signaling | 1.14E00 | 1E-01 | 3.8E-01 | *CHD4, RBBP4* |
| PAK Signaling | 1.13E00 | 5E-02 | 3.8E-01 | *KRAS, CFL1, ITGA3, ITGB1, HRAS* |
| Phospholipase C Signaling | 1.12E00 | 3.9E-02 | 3.74E-01 | *ARHGEF11, KRAS, CALM1 (includes others), ITGA3, RACK1, ITGB1, PLD2, RHOT1, HRAS* |
| Endoplasmic Reticulum Stress Pathway | 1.1E00 | 9.52E-02 | 3.68E-01 | *EIF2S1, HSP90B1* |
| Tight Junction Signaling | 1.1E00 | 4.22E-02 | 3.68E-01 | *PATJ, EPB41, OCLN, MPP5, TJP2, VAPA, SAFB* |
| Polyamine Regulation in Colon Cancer | 1.07E00 | 9.09E-02 | 3.67E-01 | *KRAS, PSMA8* |
| Branched-chain α-keto acid Dehydrogenase  Complex | 1.06E00 | 2.5E-01 | 3.67E-01 | *DBT* |
| Heme Biosynthesis from Uroporphyrinogen-  III I | 1.06E00 | 2.5E-01 | 3.67E-01 | *CPOX* |
| Glutathione Redox Reactions II | 1.06E00 | 2.5E-01 | 3.67E-01 | *GSR* |
| Proline Biosynthesis I | 1.06E00 | 2.5E-01 | 3.67E-01 | *PYCR1* |
| Cancer Drug Resistance By Drug Efflux | 1.02E00 | 6.12E-02 | 3.26E-01 | *KRAS, YBX1, HRAS* |
| Lipid Antigen Presentation by CD1 | 1E00 | 8.33E-02 | 3.2E-01 | *AP2A2, AP2M1* |
| Paxillin Signaling | 9.97E-01 | 4.55E-02 | 3.2E-01 | *KRAS, ITGA3, ITGB1, ITGA6, HRAS* |
| Gluconeogenesis I | 9.71E-01 | 8E-02 | 3.08E-01 | *GAPDH, ENO1* |
| Trehalose Degradation II (Trehalase) | 9.71E-01 | 2E-01 | 3.08E-01 | *HK1* |
| Macropinocytosis Signaling | 9.67E-01 | 4.94E-02 | 3.08E-01 | *KRAS, USP6NL, ITGB1, HRAS* |
| Semaphorin Signaling in Neurons | 9.57E-01 | 5.77E-02 | 3.05E-01 | *CFL1, ITGB1, RHOT1* |
| Unfolded protein response | 9.39E-01 | 5.66E-02 | 3E-01 | *HSPA4, HSP90B1, VCP* |
| phagosome formation | 9.36E-01 | 4.35E-02 | 3E-01 | *VTN, ITGA3, ITGB1, RHOT1, PLCD3* |
| IL-4 Signaling | 9.24E-01 | 4.76E-02 | 3E-01 | *KRAS, HMGA1, INPP5K, HRAS* |
| Axonal Guidance Signaling | 9.18E-01 | 3.17E-02 | 3E-01 | *KRAS, ARHGEF11, RACK1, ITGA3, ARPC5L, EPHA2, PLCD3, ARPC5, CFL1, ITGB1, ARPC4, PSMD14, HRAS, ARPC1A* |
| α-Adrenergic Signaling | 9.1E-01 | 4.71E-02 | 3E-01 | *KRAS, CALM1 (includes others), RACK1, HRAS* |
| Melanoma Signaling | 9.03E-01 | 5.45E-02 | 3E-01 | *KRAS, CDH1, HRAS* |
| phagosome maturation | 9.02E-01 | 4.24E-02 | 3E-01 | *ATP6V0A1, HLA-B, ATP6V1A, PRDX1, DYNLL1* |
| Proline Biosynthesis II (from Arginine) | 8.96E-01 | 1.67E-01 | 3E-01 | *PYCR1* |
| Arginine Degradation VI (Arginase 2  Pathway) | 8.96E-01 | 1.67E-01 | 3E-01 | *PYCR1* |
| Acetyl-CoA Biosynthesis I (Pyruvate  Dehydrogenase Complex) | 8.96E-01 | 1.67E-01 | 3E-01 | *DBT* |
| Apoptosis Signaling | 8.71E-01 | 4.55E-02 | 2.79E-01 | *KRAS, AIFM1, HRAS, ACIN1* |
| PDGF Signaling | 8.45E-01 | 4.44E-02 | 2.59E-01 | *KRAS, INPP5K, HRAS, CSNK2A1* |
| CD28 Signaling in T Helper Cells | 8.37E-01 | 4.03E-02 | 2.56E-01 | *ARPC5, CALM1 (includes others), ARPC4, ARPC5L, ARPC1A* |
| Reelin Signaling in Neurons | 8.21E-01 | 4.35E-02 | 2.5E-01 | *ARHGEF11, ITGA3, ITGB1, ITGA6* |
| IL-8 Signaling | 8.21E-01 | 3.57E-02 | 2.5E-01 | *KRAS, RACK1, PLD2, CSTB, RHOT1, CDH1, HRAS* |
| CCR3 Signaling in Eosinophils | 8.17E-01 | 3.97E-02 | 2.5E-01 | *KRAS, CFL1, CALM1 (includes others), RACK1, HRAS* |
| Gap Junction Signaling | 7.89E-01 | 3.66E-02 | 2.32E-01 | *CSNK1D, KRAS, CSNK1G3, PRKG2, HRAS, PLCD3* |
| GDP-glucose Biosynthesis | 7.81E-01 | 1.25E-01 | 2.32E-01 | *HK1* |
| Huntington's Disease Signaling | 7.81E-01 | 3.36E-02 | 2.32E-01 | *HSPA4, CLTA, DNAJB1, RACK1, AP2A2, PSMA8, HRAS, ATP5B* |
| Cell Cycle: G1/S Checkpoint Regulation | 7.78E-01 | 4.76E-02 | 2.32E-01 | *GNL3, RPL5, RPL11* |
| Wnt/β-catenin Signaling | 7.73E-01 | 3.61E-02 | 2.32E-01 | *RUVBL2, CSNK1D, CSNK1G3, CDH1, CDH3, CSNK2A1* |
| Endometrial Cancer Signaling | 7.64E-01 | 4.69E-02 | 2.32E-01 | *KRAS, CDH1, HRAS* |
| IL-2 Signaling | 7.64E-01 | 4.69E-02 | 2.32E-01 | *KRAS, HRAS, CSNK2A1* |
| Oncostatin M Signaling | 7.55E-01 | 5.88E-02 | 2.32E-01 | *KRAS, HRAS* |
| CTLA4 Signaling in Cytotoxic T  Lymphocytes | 7.52E-01 | 4.08E-02 | 2.32E-01 | *CLTA, AP2A2, HLA-B, AP2M1* |
| FAK Signaling | 7.52E-01 | 4.08E-02 | 2.32E-01 | *KRAS,ITGA3,ITGB1,HRAS* |
| Stearate Biosynthesis I (Animals) | 7.35E-01 | 5.71E-02 | 2.32E-01 | *DBT,ACOT9* |
| Assembly of RNA Polymerase I Complex | 7.35E-01 | 1.11E-01 | 2.32E-01 | *UBTF* |
| Glucose and Glucose-1-phosphate Degradation | 7.35E-01 | 1.11E-01 | 2.32E-01 | *HK1* |
| Heme Biosynthesis II | 7.35E-01 | 1.11E-01 | 2.32E-01 | *CPOX* |
| VEGF Signaling | 7.31E-01 | 4E-02 | 2.32E-01 | *EIF2S1,KRAS,ELAVL1,HRAS* |
| Complement System | 7.16E-01 | 5.56E-02 | 2.22E-01 | *CD59,C1QBP* |
| Role of NFAT in Regulation of the Immune Response | 6.79E-01 | 3.37E-02 | 1.9E-01 | *CSNK1D,KRAS,CALM1 (includes others),RACK1,CSNK1G3,HRAS* |
| Superpathway of Inositol Phosphate Compounds | 6.76E-01 | 3.24E-02 | 1.9E-01 | *CALM1 (includes others),ATP1A1,INPP5K,SET,SEC16A,PPFIBP2,PLCD3* |
| Antiproliferative Role of Somatostatin Receptor 2 | 6.62E-01 | 4.17E-02 | 1.89E-01 | *KRAS,RACK1,HRAS* |
| Acyl-CoA Hydrolysis | 6.57E-01 | 9.09E-02 | 1.89E-01 | *ACOT9* |
| Purine Nucleotides De Novo Biosynthesis II | 6.57E-01 | 9.09E-02 | 1.89E-01 | *IMPDH2* |
| UDP-N-acetyl-D-galactosamine Biosynthesis II | 6.57E-01 | 9.09E-02 | 1.89E-01 | *HK1* |
| Ephrin B Signaling | 6.5E-01 | 4.11E-02 | 1.89E-01 | *CFL1,RACK1,HRAS* |
| GM-CSF Signaling | 6.5E-01 | 4.11E-02 | 1.89E-01 | *KRAS,RACK1,HRAS* |
| Neuroprotective Role of THOP1 in Alzheimer's Disease | 6.48E-01 | 5E-02 | 1.89E-01 | *HLA-B,PLG* |
| Androgen Signaling | 6.34E-01 | 3.64E-02 | 1.79E-01 | *HSPA4,CALM1 (includes others),DNAJB1,RACK1* |
| Assembly of RNA Polymerase III Complex | 6.24E-01 | 8.33E-02 | 1.76E-01 | *SF3A1* |
| GDNF Family Ligand-Receptor Interactions | 6.17E-01 | 3.95E-02 | 1.76E-01 | *KRAS,PDLIM7,HRAS* |
| Role of p14/p19ARF in Tumor Suppression | 6.17E-01 | 4.76E-02 | 1.76E-01 | *UBTF,SF3A1* |
| UVC-Induced MAPK Signaling | 6.17E-01 | 4.76E-02 | 1.76E-01 | *KRAS,HRAS* |
| iNOS Signaling | 6.02E-01 | 4.65E-02 | 1.68E-01 | *HMGA1,CALM1 (includes others)* |
| HGF Signaling | 5.99E-01 | 3.51E-02 | 1.68E-01 | *KRAS,ITGA3,ITGB1,HRAS* |
| Regulation of IL-2 Expression in Activated and Anergic T Lymphocytes | 5.96E-01 | 3.85E-02 | 1.68E-01 | *KRAS,CALM1 (includes others),HRAS* |
| Choline Biosynthesis III | 5.94E-01 | 7.69E-02 | 1.68E-01 | *PLD2* |
| Cardiac Hypertrophy Signaling | 5.79E-01 | 3.02E-02 | 1.58E-01 | *HSPB1,KRAS,CALM1 (includes others),RACK1,RHOT1,HRAS,PLCD3* |
| Synaptic Long Term Potentiation | 5.74E-01 | 3.42E-02 | 1.58E-01 | *KRAS,CALM1 (includes others),HRAS,PLCD3* |
| Sperm Motility | 5.66E-01 | 3.39E-02 | 1.58E-01 | *TWF1,CALM1 (includes others),PRKG2,PLCD3* |
| nNOS Signaling in Skeletal Muscle Cells | 5.66E-01 | 7.14E-02 | 1.58E-01 | *CALM1 (includes others)* |
| Urate Biosynthesis/Inosine 5'-phosphate Degradation | 5.66E-01 | 7.14E-02 | 1.58E-01 | *IMPDH2* |
| Dopamine-DARPP32 Feedback in cAMP Signaling | 5.54E-01 | 3.16E-02 | 1.48E-01 | *CSNK1D,CALM1 (includes others),CSNK1G3,PRKG2,PLCD3* |
| Telomere Extension by Telomerase | 5.41E-01 | 6.67E-02 | 1.44E-01 | *XRCC6* |
| Thrombin Signaling | 5.36E-01 | 3E-02 | 1.44E-01 | *ARHGEF11,KRAS,RACK1,RHOT1,HRAS,PLCD3* |
| Cell Cycle: G2/M DNA Damage Checkpoint Regulation | 5.23E-01 | 4.08E-02 | 1.44E-01 | *TOP2A,TOP2B* |
| PI3K Signaling in B Lymphocytes | 5.21E-01 | 3.23E-02 | 1.44E-01 | *KRAS,CALM1 (includes others),HRAS,PLCD3* |
| Bladder Cancer Signaling | 5.2E-01 | 3.49E-02 | 1.44E-01 | *KRAS,CDH1,HRAS* |
| Granzyme B Signaling | 5.17E-01 | 6.25E-02 | 1.44E-01 | *NUMA1* |
| Methionine Degradation I (to Homocysteine) | 5.17E-01 | 6.25E-02 | 1.44E-01 | *PRMT1* |
| CXCR4 Signaling | 5.15E-01 | 3.05E-02 | 1.44E-01 | *ARHGEF11,KRAS,RACK1,RHOT1,HRAS* |
| Amyloid Processing | 5.11E-01 | 4E-02 | 1.44E-01 | *CSNK1D,CSNK2A1* |
| Granulocyte Adhesion and Diapedesis | 5.09E-01 | 3.03E-02 | 1.44E-01 | *HSPB1,GLG1,ITGA3,ITGB1,ITGA6* |
| PPARα/RXRα Activation | 5.03E-01 | 3.01E-02 | 1.44E-01 | *KRAS,HSP90B1,AP2A2,HRAS,PLCD3* |
| G Beta Gamma Signaling | 5.02E-01 | 3.41E-02 | 1.44E-01 | *KRAS,RACK1,HRAS* |
| HER-2 Signaling in Breast Cancer | 5.02E-01 | 3.41E-02 | 1.44E-01 | *KRAS,ITGB1,HRAS* |
| IL-6 Signaling | 5E-01 | 3.15E-02 | 1.44E-01 | *HSPB1,KRAS,HRAS,CSNK2A1* |
| Granzyme A Signaling | 4.96E-01 | 5.88E-02 | 1.44E-01 | *SET* |
| γ-linolenate Biosynthesis II (Animals) | 4.96E-01 | 5.88E-02 | 1.44E-01 | *CYB5R3* |
| Estrogen Receptor Signaling | 4.93E-01 | 3.12E-02 | 1.44E-01 | *KRAS,PELP1,HNRNPD,HRAS* |
| Tec Kinase Signaling | 4.91E-01 | 2.98E-02 | 1.44E-01 | *ITGA3,RACK1,ITGB1,GTF2I,RHOT1* |
| PPAR Signaling | 4.86E-01 | 3.33E-02 | 1.44E-01 | *KRAS,HSP90B1,HRAS* |
| Gα12/13 Signaling | 4.79E-01 | 3.08E-02 | 1.44E-01 | *KRAS,CDH1,CDH3,HRAS* |
| p70S6K Signaling | 4.79E-01 | 3.08E-02 | 1.44E-01 | *KRAS,HRAS,EEF2,PLCD3* |
| FcγRIIB Signaling in B Lymphocytes | 4.78E-01 | 3.77E-02 | 1.44E-01 | *KRAS,HRAS* |
| Valine Degradation I | 4.75E-01 | 5.56E-02 | 1.44E-01 | *DBT* |
| Cysteine Biosynthesis III (mammalia) | 4.75E-01 | 5.56E-02 | 1.44E-01 | *PRMT1* |
| P2Y Purigenic Receptor Signaling Pathway | 4.73E-01 | 3.05E-02 | 1.44E-01 | *KRAS,RACK1,HRAS,PLCD3* |
| Prostate Cancer Signaling | 4.7E-01 | 3.26E-02 | 1.44E-01 | *KRAS,HSP90B1,HRAS* |
| Role of CHK Proteins in Cell Cycle Checkpoint Control | 4.57E-01 | 3.64E-02 | 1.39E-01 | *RFC4,PCNA* |
| GADD45 Signaling | 4.57E-01 | 5.26E-02 | 1.39E-01 | *PCNA* |
| Purine Nucleotides Degradation II (Aerobic) | 4.57E-01 | 5.26E-02 | 1.39E-01 | *IMPDH2* |
| Maturity Onset Diabetes of Young (MODY) Signaling | 4.39E-01 | 5E-02 | 1.24E-01 | *GAPDH* |
| Hereditary Breast Cancer Signaling | 4.23E-01 | 2.88E-02 | 1.19E-01 | *KRAS,SMARCA2,RFC4,HRAS* |
| 3-phosphoinositide Degradation | 4.23E-01 | 2.88E-02 | 1.19E-01 | *ATP1A1,INPP5K,SET,PPFIBP2* |
| Phospholipases | 4.18E-01 | 3.39E-02 | 1.19E-01 | *PLD2,PLCD3* |
| Synaptic Long Term Depression | 4.17E-01 | 2.86E-02 | 1.19E-01 | *KRAS,PRKG2,HRAS,PLCD3* |
| D-myo-inositol-5-phosphate Metabolism | 4.17E-01 | 2.86E-02 | 1.19E-01 | *ATP1A1,SET,PPFIBP2,PLCD3* |
| CREB Signaling in Neurons | 4.14E-01 | 2.75E-02 | 1.19E-01 | *KRAS,CALM1 (includes others),RACK1,HRAS,PLCD3* |
| B Cell Receptor Signaling | 4.14E-01 | 2.75E-02 | 1.19E-01 | *KRAS,CFL1,CALM1 (includes others),INPP5K,HRAS* |
| CNTF Signaling | 4.09E-01 | 3.33E-02 | 1.19E-01 | *KRAS,HRAS* |
| Ephrin A Signaling | 4.09E-01 | 3.33E-02 | 1.19E-01 | *CFL1,EPHA2* |
| Cholecystokinin/Gastrin-mediated Signaling | 4.05E-01 | 2.97E-02 | 1.19E-01 | *KRAS,RHOT1,HRAS* |
| UVA-Induced MAPK Signaling | 4.05E-01 | 2.97E-02 | 1.19E-01 | *KRAS,HRAS,PLCD3* |
| ERK5 Signaling | 3.84E-01 | 3.17E-02 | 1.13E-01 | *KRAS,HRAS* |
| Hypoxia Signaling in the Cardiovascular System | 3.84E-01 | 3.17E-02 | 1.13E-01 | *CSNK1D,HSP90B1* |
| Role of NFAT in Cardiac Hypertrophy | 3.81E-01 | 2.65E-02 | 1.13E-01 | *KRAS,CALM1 (includes others),RACK1,HRAS,PLCD3* |
| Tumoricidal Function of Hepatic Natural Killer Cells | 3.78E-01 | 4.17E-02 | 1.13E-01 | *AIFM1* |
| CDP-diacylglycerol Biosynthesis I | 3.78E-01 | 4.17E-02 | 1.13E-01 | *AGPAT5* |
| Glutathione-mediated Detoxification | 3.78E-01 | 4.17E-02 | 1.13E-01 | *MGST3* |
| IGF-1 Signaling | 3.73E-01 | 2.83E-02 | 1.13E-01 | *KRAS,HRAS,CSNK2A1* |
| Thrombopoietin Signaling | 3.68E-01 | 3.08E-02 | 1.13E-01 | *KRAS,HRAS* |
| Telomerase Signaling | 3.61E-01 | 2.78E-02 | 1.13E-01 | *KRAS,HSP90B1,HRAS* |
| Gαs Signaling | 3.61E-01 | 2.78E-02 | 1.13E-01 | *ADD1,RACK1,ADD3* |
| Nitric Oxide Signaling in the Cardiovascular System | 3.61E-01 | 2.78E-02 | 1.13E-01 | *CALM1 (includes others),HSP90B1,PRKG2* |
| T Cell Receptor Signaling | 3.61E-01 | 2.78E-02 | 1.13E-01 | *KRAS,CALM1 (includes others),HRAS* |
| GABA Receptor Signaling | 3.6E-01 | 3.03E-02 | 1.13E-01 | *AP2A2,AP2M1* |
| Glioma Signaling | 3.55E-01 | 2.75E-02 | 1.13E-01 | *KRAS,CALM1 (includes others),HRAS* |
| CCR5 Signaling in Macrophages | 3.53E-01 | 2.99E-02 | 1.13E-01 | *CALM1 (includes others),RACK1* |
| Phosphatidylglycerol Biosynthesis II (Non-plastidic) | 3.52E-01 | 3.85E-02 | 1.13E-01 | *AGPAT5* |
| ErbB2-ErbB3 Signaling | 3.45E-01 | 2.94E-02 | 1.13E-01 | *KRAS,HRAS* |
| EGF Signaling | 3.45E-01 | 2.94E-02 | 1.13E-01 | *HRAS,CSNK2A1* |
| p53 Signaling | 3.43E-01 | 2.7E-02 | 1.13E-01 | *GNL3,CSNK1D,PCNA* |
| ERK/MAPK Signaling | 3.41E-01 | 2.53E-02 | 1.13E-01 | *HSPB1,KRAS,ITGA3,ITGB1,HRAS* |
| IL-15 Production | 3.4E-01 | 3.7E-02 | 1.13E-01 | *TWF1* |
| Cell Cycle Control of Chromosomal Replication | 3.4E-01 | 3.7E-02 | 1.13E-01 | *MCM5* |
| D-myo-inositol (1,4,5)-Trisphosphate Biosynthesis | 3.4E-01 | 3.7E-02 | 1.13E-01 | *PLCD3* |
| Melatonin Signaling | 3.38E-01 | 2.9E-02 | 1.13E-01 | *CALM1 (includes others),PLCD3* |
| Role of JAK1 and JAK3 in γc Cytokine Signaling | 3.38E-01 | 2.9E-02 | 1.13E-01 | *KRAS,HRAS* |
| Myc Mediated Apoptosis Signaling | 3.31E-01 | 2.86E-02 | 1.13E-01 | *KRAS,HRAS* |
| Role of MAPK Signaling in the Pathogenesis of Influenza | 3.31E-01 | 2.86E-02 | 1.13E-01 | *KRAS,HRAS* |
| ErbB4 Signaling | 3.31E-01 | 2.86E-02 | 1.13E-01 | *KRAS,HRAS* |
| Glioblastoma Multiforme Signaling | 3.26E-01 | 2.53E-02 | 1.13E-01 | *KRAS,RHOT1,HRAS,PLCD3* |
| Gαq Signaling | 3.26E-01 | 2.53E-02 | 1.13E-01 | *CALM1 (includes others),RACK1,PLD2,RHOT1* |
| Breast Cancer Regulation by Stathmin1 | 3.25E-01 | 2.48E-02 | 1.13E-01 | *ARHGEF11,KRAS,CALM1 (includes others),RACK1,HRAS* |
| Role of Wnt/GSK-3β Signaling in the Pathogenesis of Influenza | 3.18E-01 | 2.78E-02 | 1.07E-01 | *CSNK1D,CSNK1G3* |
| STAT3 Pathway | 3.11E-01 | 2.74E-02 | 1.07E-01 | *KRAS,HRAS* |
| BMP signaling pathway | 3.05E-01 | 2.7E-02 | 1.07E-01 | *KRAS,HRAS* |
| Fc Epsilon RI Signaling | 3.02E-01 | 2.52E-02 | 1.07E-01 | *KRAS,INPP5K,HRAS* |
| Cytotoxic T Lymphocyte-mediated Apoptosis of Target Cells | 2.97E-01 | 3.23E-02 | 1.07E-01 | *HLA-B* |
| G Protein Signaling Mediated by Tubby | 2.97E-01 | 3.23E-02 | 1.07E-01 | *RACK1* |
| Superpathway of Methionine Degradation | 2.97E-01 | 3.23E-02 | 1.07E-01 | *PRMT1* |
| Natural Killer Cell Signaling | 2.97E-01 | 2.5E-02 | 1.07E-01 | *KRAS,INPP5K,HRAS* |
| Gαi Signaling | 2.97E-01 | 2.5E-02 | 1.07E-01 | *KRAS,RACK1,HRAS* |
| IL-15 Signaling | 2.93E-01 | 2.63E-02 | 1.07E-01 | *KRAS,HRAS* |
| Angiopoietin Signaling | 2.93E-01 | 2.63E-02 | 1.07E-01 | *KRAS,HRAS* |
| Neurotrophin/TRK Signaling | 2.93E-01 | 2.63E-02 | 1.07E-01 | *KRAS,HRAS* |
| D-myo-inositol (1,4,5,6)-Tetrakisphosphate Biosynthesis | 2.87E-01 | 2.46E-02 | 1.07E-01 | *ATP1A1,SET,PPFIBP2* |
| D-myo-inositol (3,4,5,6)-tetrakisphosphate Biosynthesis | 2.87E-01 | 2.46E-02 | 1.07E-01 | *ATP1A1,SET,PPFIBP2* |
| Non-Small Cell Lung Cancer Signaling | 2.87E-01 | 2.6E-02 | 1.07E-01 | *KRAS,HRAS* |
| Estrogen-Dependent Breast Cancer Signaling | 2.87E-01 | 2.6E-02 | 1.07E-01 | *KRAS,HRAS* |
| Role of BRCA1 in DNA Damage Response | 2.81E-01 | 2.56E-02 | 1.06E-01 | *SMARCA2,RFC4* |
| Circadian Rhythm Signaling | 2.79E-01 | 3.03E-02 | 1.06E-01 | *CSNK1D* |
| Calcium Signaling | 2.76E-01 | 2.35E-02 | 1.06E-01 | *LETM1,CALM1 (includes others),MCU,ASPH* |
| Erythropoietin Signaling | 2.75E-01 | 2.53E-02 | 1.06E-01 | *KRAS,HRAS* |
| Renal Cell Carcinoma Signaling | 2.75E-01 | 2.53E-02 | 1.06E-01 | *KRAS,HRAS* |
| Cellular Effects of Sildenafil (Viagra) | 2.74E-01 | 2.4E-02 | 1.06E-01 | *CALM1 (includes others),PRKG2,PLCD3* |
| Coagulation System | 2.62E-01 | 2.86E-02 | 1.02E-01 | *PLG* |
| Cell Cycle Regulation by BTG Family Proteins | 2.62E-01 | 2.86E-02 | 1.02E-01 | *PRMT1* |
| FLT3 Signaling in Hematopoietic Progenitor Cells | 2.59E-01 | 2.44E-02 | 1.02E-01 | *KRAS,HRAS* |
| Agranulocyte Adhesion and Diapedesis | 2.58E-01 | 2.29E-02 | 1.02E-01 | *GLG1,ITGA3,ITGB1,ITGA6* |
| 14-3-3-mediated Signaling | 2.57E-01 | 2.33E-02 | 1.02E-01 | *KRAS,HRAS,PLCD3* |
| IL-3 Signaling | 2.54E-01 | 2.41E-02 | 1.02E-01 | *KRAS,HRAS* |
| Prolactin Signaling | 2.54E-01 | 2.41E-02 | 1.02E-01 | *KRAS,HRAS* |
| JAK/Stat Signaling | 2.54E-01 | 2.41E-02 | 1.02E-01 | *KRAS,HRAS* |
| PEDF Signaling | 2.49E-01 | 2.38E-02 | 1.01E-01 | *KRAS,HRAS* |
| HMGB1 Signaling | 2.49E-01 | 2.29E-02 | 1.01E-01 | *KRAS,RHOT1,HRAS* |
| tRNA Splicing | 2.46E-01 | 2.7E-02 | 1.01E-01 | *SMPDL3B* |
| Antigen Presentation Pathway | 2.46E-01 | 2.7E-02 | 1.01E-01 | *HLA-B* |
| IL-17 Signaling | 2.44E-01 | 2.35E-02 | 1.01E-01 | *KRAS,HRAS* |
| LPS-stimulated MAPK Signaling | 2.39E-01 | 2.33E-02 | 9.95E-02 | *KRAS,HRAS* |
| tRNA Charging | 2.39E-01 | 2.63E-02 | 9.95E-02 | *IARS* |
| Cardiac β-adrenergic Signaling | 2.37E-01 | 2.24E-02 | 9.95E-02 | *SMPDL3B,AKAP2,RACK1* |
| TGF-β Signaling | 2.35E-01 | 2.3E-02 | 9.89E-02 | *KRAS,HRAS* |
| VEGF Family Ligand-Receptor Interactions | 2.3E-01 | 2.27E-02 | 9.65E-02 | *KRAS,HRAS* |
| Crosstalk between Dendritic Cells and Natural Killer Cells | 2.25E-01 | 2.25E-02 | 9.65E-02 | *HLA-B,FSCN1* |
| Role of PKR in Interferon Induction and Antiviral Response | 2.25E-01 | 2.5E-02 | 9.65E-02 | *EIF2S1* |
| Transcriptional Regulatory Network in Embryonic Stem Cells | 2.25E-01 | 2.5E-02 | 9.65E-02 | *SET* |
| Mechanisms of Viral Exit from Host Cells | 2.18E-01 | 2.44E-02 | 9.17E-02 | *CHMP4B* |
| Autoimmune Thyroid Disease Signaling | 2.12E-01 | 2.38E-02 | 8.71E-02 | *HLA-B* |
| Graft-versus-Host Disease Signaling | 2E-01 | 2.27E-02 | 7.69E-02 | *HLA-B* |
