# Supplementary material for: Proteins that interact with calgranulin B in the human colon cancer cell line HCT-116
Source: Oncotarget. 2016 Dec 27;8(4):6819–32. doi: 10.18632/oncotarget.14301 (PMC5351672; doi:10.18632/oncotarget.14301)
Supplement: Supplementary file 5 [file oncotarget-08-6819-s005.docx]

**Supplementary Table 4.** Mechanistic networks for calgranulin B-interacting proteins including the molecules, top diseases, and function information in each network.

| **Network** | **Molecules in Network** | **Score** | **Focus Molecules** | **Top diseases and Function** |
| --- | --- | --- | --- | --- |
| Network 1 | *ACIN1, ALYREF, ARHGAP23, BCLAF1, COIL, DDX41, DHX15, EFTUD2, FAT1, PCBP1, PCBP2, PNN, POLDIP3, PRPF19, PRPF8, RBM25, RNPS1, SAFB, SF3A1, SF3B2, SF3B3, snRNP, SNRNP200, SNRPA, SNRPA1, SNRPD3, SNW1, SPIN1, SRRM2, SRSF1, SRSF3, SRSF6, SRSF7, SRSF9, Vegf* | 54 | 33 | RNA Post-Transcriptional Modification, Infectious Diseases, Organismal Injury and Abnormalities |
| Network 2 | *AKAP8L, BRIX1, EBNA1BP2, EIF3, EIF3A, EIF3C, EIF3D, EIF3E, Eif4g, EIF6, FAM83B, FAM98A, HNRNPH3, IMPDH2, LYAR, MAGEB2, MAP7, MATR3, MSLN, MTCH2, NAT10, PI3K (complex), PKP4, PPP1R13L, RPL10, RPL17, RPL21, RPL24, RPL3, RPL34, RPS11, RPS16, SEC16A, SLMAP, Vla-4* | 49 | 31 | Cancer, Cell Death and Survival, Organismal Injury and Abnormalities |
| Network 3 | *ACAD11, Alpha tubulin, AMOT, AMOTL1, CORO1C, CPNE4, CSNK1G3, CSTB, DDX18, DECR2, Dlg, DLG1, DLG5, Dynein, DYNLL1, EPB41, EPB41L2, GSR, Guk, INF2, LIN7C, LRCH1, LRCH3, MGST3, MPP5, MTDH, NFkB (complex), NUMA1, PATJ, PDCD11, PLCD3, PLEKHG3, PLOD1, TJP2, ZDHHC5* | 46 | 30 | Cardiovascular System Development and Function, Cell Death and Survival, Cell Morphology |
| Network 4 | *CCT3, CCT4, CCT5, CCT6A, CCT8, CD59, EEF1D, EPHA2, estrogen receptor, GCN1, H2AFY, HBA1/HBA2, HBB, HDL, hemoglobin, HLA-B, ILF3, LIG3, mediator, OCIAD1, POLRMT, PRDX1, PRMT1, RPL23A, RPN1, SERBP1, SET, SON, SRC (family), TCP1, USP46, VCP, WDR43, WDR48, XRN2* | 46 | 30 | Cellular Assembly and Organization, Cell-To-Cell Signaling and Interaction, Reproductive System Development and Function |
| Network 5 | *AIFM1, Calcineurin protein(s), CK1, CLTA, COX4I1, COX5A, COX5B, CSNK1D, DBT, DHX30, DSC2, DSG1, EPS8, FTSJ3, GAPDH, GNL3, Hsp70, IGF2BP2, LOC102724159/PWP2, MT-CO2, NOP2, P38 MAPK, PPFIBP2, PRNP, QPCTL, RALY, RNase A, RPL6, RPL31, SLC25A13, SMU1, Sod, TARDBP, USP6NL, YBX1* | 44 | 29 | Cell Death and Survival, Cellular Compromise, Neurological Disease |
| Network 6 | *Actin,AGPAT5,Arp2/3,ARPC4,ARPC5,ARPC1A,ARPC5L,BAG3,BSG,CALM1 (includes others),CEBPZ, Cyclin B,Focal adhesion kinase,KRR1,MLF2,MRPL10,NGDN,NOL10,PDLIM7,PSMD2,PUM3,PYCR1, Ras homolog,RHOT1,RPL14,RPL15,RPL26,RPS8,RPS3A,SEC61A1,TCR,TPD52L2,TWF2,UBAP2L, UTP20* | 44 | 29 | RNA Post-Transcriptional Modification, Cardiovascular Disease, Developmental Disorder |
| Network 7 | *Alpha Actinin,Alpha catenin,Cadherin,Calcineurin A,CDC73,CDCP1,CDH3,CNOT1,ERK,GOT, IMP4, Importin beta, KIDINS220, KPNB1, LGALS3BP, MARCKSL1, MPHOSPH10, NOP58, NUP93, NUP98, NUP133,NUP210,RAB11A,RANBP2,RAVER1,RCC1,RPL18A,SCRIB,SEC13,TES,TNKS1BP1,TOMM20,TOMM22,TOMM70,UACA* | 41 | 28 | Cell Signaling, Post-Translational Modification, Protein Synthesis |
| Network 8 | *ACADVL,Akt,APOOL,C1QBP,COX6C,CYC1,Cytochrome bc1,cytochrome-c oxidase,Fascin,HK1,LETM1, MCU, Mitochondrial complex 1, NADH dehydrogenase, NDUFA9, NDUFA10, NDUFA12, NDUFS1, NDUFS2,NDUFS3,NDUFV1,NDUFV3,PRKG2,S100,S100A10,S100A11,S100A16,SPRR3,TFAM,TRPM4,UQCRC1,UQCRC2,UQCRFS1,VAPA,Vdac* | 39 | 27 | Developmental Disorder, Hereditary Disorder, Metabolic Disease |
| Network 9 | *BOP1,CHD4,CLNS1A,DDX21,DDX47,DMAP1,FSCN1,GAR1,Hdac1/2,HISTONE,HMGN1,Mi2,MYBBP1A,PCNA,PES1,Pkc(s),RFC4,Rnr,RPA,RPL35,RPS5,RPS9,RPS18,RPS4X,RRP1B,RSL1D1,RUVBL2,SMARCA5,TIP60,Top2,TOP2A,TOP2B,UBTF,UHRF1,WDR12* | 39 | 27 | RNA Post-Transcriptional Modification, Cancer, Cardiovascular Disease |
| Network 10 | *BMS1,CBX3,CDH1,CSNK2A1,CYB5R3,ENO1,ERBIN,Fcer1,FMR1,HMGA1,HNRNPAB,HNRNPD,LRRC59,MAK16,MKI67,MTFR1,PI3K (family),PKM,PTBP1,Rac,RACK1,RBBP4,RNA polymerase II,RPS7,RRP9, Secretase gamma,Shc,SNAP23,SND1,Sos,SUN1,TCF,UTP18,WDR36,XRCC6* | 39 | 27 | Cellular Movement, Cell Cycle, DNA Replication, Recombination, and Repair |
