# Supplementary material for: Proteins that interact with calgranulin B in the human colon cancer cell line HCT-116
Source: Oncotarget. 2016 Dec 27;8(4):6819–32. doi: 10.18632/oncotarget.14301 (PMC5351672; doi:10.18632/oncotarget.14301)
Supplement: Supplementary file 6 [file oncotarget-08-6819-s006.docx]

**Supplementary Table 5.** Upstream regulators and target molecules identified as calgranulin B-interacting proteins.

| Upstream Regulator | Molecule Type | p-value of  overlap | Target molecules in dataset |
| --- | --- | --- | --- |
| MYCN | transcription regulator | 6.63E-24 | *CKAP4, EEF1D, EEF1G, EEF2, EIF3C, GAPDH, HMGA1, ITGA3, ITGB1, LGALS1, PHB, RBBP4, RPL10, RPL11, RPL13, RPL17, RPL18, RPL18A, RPL21, RPL23A, RPL24, RPL26, RPL3, RPL30, RPL31, RPL35, RPL35A, RPL5, RPL6, RPS16, RPS19, RPS23, RPS27, RPS3A, RPS4X, RPS5, RPS7, RPS8, RPS9, S100A10* |
| RICTOR | other | 4.09E-23 | *ATP5B, ATP5O, ATP6V0A1, ATP6V1A, BSG, COX4I1, COX5A, CYC1, NDUFA10, NDUFA9, NDUFS1, NDUFS2, NDUFS3, NDUFV1, NDUFV3, PSMA8, PSMD14, PSMD2, RPL10, RPL10A, RPL11, RPL14, RPL17, RPL18, RPL21, RPL26, RPL30, RPL35A, RPL6, RPS11, RPS18, RPS19, RPS23, RPS5, RPS8, RPS9, UQCRC1, UQCRC2, UQCRFS1* |
| MYC | transcription regulator | 1.53E-21 | *ADD1, BOP1, C1QBP, CCT3, CDH1, COX5B, CSTB, DDX18, DDX21, EEF2, EFTUD2, EIF2S1, EIF3D, ELAVL1, ENO1, EPHA2, GAPDH, GLG1, GSR, HLA-B, HMGA1, HNRNPAB, HNRNPD, HSPB1, ITGA3, ITGA6, ITGB1, KRAS, LGALS1, MCM5, MGST3, MKI67, MYBBP1A, NOP58, PCNA, PDLIM7, PHB, PKM, POLDIP3, PRMT1, PTBP1, PYCR1, RANBP2, RBBP4, RCC1, RPL10, RPL13, RPL21, RPL26, RPL3, RPL30, RPL35, RPL5, RPL6, RPS16, RPS18, RPS19, RPS23, RPS27, RPS7, RPS9, RRP1B, RUVBL2, S100A10, SLC7A5, SPRR3, SRSF1, TES, TFAM, TRAP1, YBX1, YME1L1* |
| CST5 | other | 1.87E-13 | *BRIX1, CDH1, CEBPZ, DDX18, DDX21, EBNA1BP2, EEF1D, EEF1G, GAR1, GNL3, KRR1, LOC102724159/PWP2, LYAR, MAK16, NOP2, NUMA1, NUP98, PCBP1, PRDX1, PRPF8, RSL1D1, S100A11, SRSF1, SRSF9, TARDBP, TBL3, TOMM22, UTP18, WDR3, WDR36* |
| POLG | enzyme | 1.28E-08 | *MT-CO2, RACK1, RPL13, RPL14, RPL5, RPS16, RPS19, RPS5* |
| HSF2 | transcription regulator | 1.34E-07 | *CCT3, CCT4, CCT5, CCT6A, CCT8, HSPA4, HSPB1, TCP1* |
| SYVN1 | transporter | 1.57E-07 | *ATP1A1, CALM1 (includes others), EPHA2, HSPB1, ITGA3, ITGA6, ITGB1, LGALS3BP, PCBP1, PLD2, PTBP1, RPL10, RPL18, SLC7A5, SNAP23* |
| mir-1 | microrna | 4.17E-07 | *ADAR, ADD1, ATP6V0A1, CAPRIN1, DHX15, HSP90B1, KRAS, MTX1, PICALM, RRBP1, SEC61A1, SRSF9, TWF1* |
| HNF4A | transcription regulator | 6.83E-07 | *ACIN1, ARMC1, ARPC5, BCLAF1, CACNA2D2, CBX3, CCT8, CDH1, CLTA, CSNK2A1, DBT, DDOST, DDX10, DDX18, DDX27, DDX41, DDX47, DHDDS, DSC2, EFTUD2, EPHA2, ERLIN1, FLOT1, GAPDH, GDF15, GNL3, GTF2I, HLA-B, HSP90B1, ITGA6, KPNB1, KRR1, LGALS3, LIN7C, MAP7, MGST3, NAT10, NDUFS1, NDUFS3, NDUFV1, OCLN, PATJ, PCNA, PHB, PKM, PLD2, PLG, POLRMT, PPFIBP2, PRMT1, RAB11A, RBBP4, RPL10, RPL18, RPL18A, RPL31, RPS18, RUVBL2, S100A9, SLC25A13, SMARCA5, SNAP23, SNRPA, SNRPD3, SNW1, SPRR3, SRSF1, SSBP1, SYNPO, USP46, UTP18, VTN, WDR12, YBX1* |
| E2F1 | transcription regulator | 1.68E-06 | *CALD1, CALM1 (includes others), CCT4, COX4I1, COX5A, HMGA1, HMGN1, HNRNPD, HSP90B1, MCM5, PCNA, PHB, PSMD2, RBBP4, RFC4, RPS16, RSL1D1, SAFB, SMARCA5, SRSF1, TFAM, TOP2A, TOP2B, TRAP1, UHRF1, VCP* |
| NRF1 | transcription regulator | 2.17E-06 | *ATP1A1, COX4I1, COX5A, COX5B, FMR1, HBB, TFAM, TOMM20* |
| APP | other | 2.64E-06 | *AIFM1, ATP5B, ATP6V1A, BSG, C1QBP, CD59, CDH1, CDH3, CFL1, CLTA, CSNK2A1, EEF1G, ENO1, GAPDH, GDF15, HBA1/HBA2, HK1, HMGA1, HSPB1, IGF2BP2, ITGA3, ITGA6, KPNB1, LGALS3, MRPL10, MT-CO2, OCLN, PKM, PRMT1, PRNP, RANBP2, RFC4, S100A8, SET, SMARCA2, THSD4, TOP2A, TOP2B* |
| miR-1-3p  (and other  miRNAs  w/seed  GGAAUGU) | mature microrna | 2.65E-06 | *ADAR, ATP6V0A1, CDCP1, COIL, CORO1C, CPOX, DHX15, DNAJB1, LIN7C, MTX1, PDLIM7, PICALM, PTBP1, SRSF9, TWF1, UHRF1* |
| RRP1B | other | 6.47E-06 | *MCM5, PCNA, RPL11, RPL13, RPL14, RPL6, RPS19, RPS5, RPS8, RPS9, TOP2A* |
| MMP3 | peptidase | 7.88E-06 | *ADAR, CAPRIN1, EFTUD2, PDCD11, PNN, SAFB, SF3A1, SRSF1, SRSF3, TARDBP, YBX1* |
| LONP1 | peptidase | 8.55E-06 | *C1QBP, COX5A, HSPB1, MT-CO2, S100A11, TFAM, TRAP1, UQCRFS1* |
| INSR | kinase | 9.62E-06 | *ACADVL, ATP5B, ATP5O, CEBPZ, CFL1, COX4I1, CSTB, CYC1, GAPDH, LGALS1, MARCKSL1, MCM5, NDUFA9, RPL5, RPS16, RUVBL2, TFAM, UHRF1, UQCRC1, UQCRC2, YBX1* |
| IL3 | cytokine | 1.25E-05 | *CCT5, CEBPZ, HBA1/HBA2, HK1, HSP90B1, IMPDH2, KPNB1, MCM5, MLF2, RPL10, RPL11, RPL13, RPL3, RPL5, RPL6, RPS16, RPS19, RPS7, SSBP1, YBX1* |
| FLT1 | kinase | 1.87E-05 | *ADD1, CSNK2A1, HK1, HLA-B, LIN7C, PTBP1, RBBP4, SNAP23, TOMM22* |
| KDM5A | transcription regulator | 6.95E-05 | *ATP1A1, COX4I1, DNAJC11, ITGB1, NDUFA10, NDUFA9, NDUFS2, NDUFV1, TOMM22, UQCRC1, UQCRFS1* |
| IL15 | cytokine | 7.30E-05 | *CALM1 (includes others), CD59, CDH1, COX6C, DDX18, GAPDH, H2AFY, ITGB1, MCM5, MKI67, MSLN, PCBP2, PCNA, RPS3A, S100A11, SET, SRSF7, SYNPO, TJP2, TOP2A* |
| CD3 | complex | 7.39E-05 | *CALM1 (includes others), COX5A, COX6C, CYC1, DDX18, GAPDH, H2AFY, HMGN1, HSP90B1, MCM5, MSLN, PCBP2, PCNA, PHB, PUM3, RANBP2, RPL30, RPL35A, RPL6, RPS3A, S100A11, SMPDL3B, SRSF1, SRSF7, SYNPO, TARDBP, TMPO, TOP2A, XRCC6* |
| RB1 | transcription regulator | 8.50E-05 | *ATP1A1, CDH1, COX4I1, DNAJC11, EPS8, HRAS, MCM5, NDUFA10, NDUFA9, NDUFS2, NDUFV1, PCNA, RFC4, RSL1D1, SAFB, SMARCA5, TMPO, TOMM22, UQCRC1, UQCRFS1* |
| NUBPL | other | 8.81E-05 | *NDUFS1, NDUFS3, NDUFV1* |
| WT1 | transcription regulator | 8.87E-05 | *CAPRIN1, CDC73, CDH1, EPS8, GSR, HSP90B1, KRAS, LGALS3, MSLN, NUP98, SEC13, SRSF6, TARDBP, TRAP1, YBX1* |
| MTOR | kinase | 9.65E-05 | *AIFM1, CDH1, COX4I1, EIF3A, ENO1, GLG1, HBA1/HBA2, HSP90B1, HSPB1, PKM, PTBP1, RPS11, RPS18, SND1, UGGT1, UQCRC2* |
| SRSF2 | transcription regulator | 1.20E-04 | *CDH1, PTBP1, SRSF1, SRSF6, SRSF7* |
| Mek | group | 1.27E-04 | *CDCP1, CDH1, CEBPZ, DDX21, EPHA2, GNL3, HSPB1, ITGA6, ITGB1, LYAR, NOP58, TOP2A* |
| MAPT | other | 1.44E-04 | *ATP5B, ATP6V1A, BSG, C1QBP, CFL1, CLTA, EEF1G, ENO1, GAPDH, HBA1/HBA2, HK1, NDUFS3, PKM, PRNP, TOP2B* |
| PLN | transporter | 1.60E-04 | *ATP1A1, ENO1, HRAS, HSP90B1, ITGB1, RACK1, VCP* |
| FOS | transcription regulator | 2.06E-04 | *ARPC5, CDH1, DLG1, ENO1, EPS8, GSR, HBA1/HBA2, HLA-B, HSP90B1, ITGA6, ITGB1, LGALS3, LGALS3BP, MCM5, MSLN, PRDX1, RBBP4, RPS18, RPS7, RPS9, S100A10, S100A8, S100A9, SNAP23* |
| OMA1 | peptidase | 2.15E-04 | *ACADVL, COX4I1, NDUFA9, UQCRC2* |
| PNN | other | 2.15E-04 | *BSG, CDH1, GDF15, SRSF1* |
| SRSF1 | other | 2.16E-04 | *CDH1, PTBP1, SRSF1, SRSF6, SRSF7* |
| CLDN7 | other | 2.65E-04 | *CKAP4, DNAJB6, HLA-B, LGALS3, MCU, PRNP, RPL34, RPS7, RSL1D1, SPRR3* |
| TP53 | transcription regulator | 2.75E-04 | *ACADVL, ADD3, ATP1A1, CD59, CDH1, CDH3, CPOX, CSNK1D, CSTB, DAPK3, DLG1, EPHA2, EPS8, FAT1, GAPDH, GDF15, GNL3, GSR, H2AFY, HLA-B, HRAS, HSPB1, KPNB1, LGALS3, MCM5, MKI67, MLF2, MT-CO2, MTDH, PCNA, PPP1R13L, PRNP, PSMD2, RALY, RBBP4, RFC4, RPN1, RUVBL2, SEC61A1, SON, SRSF3, SSH1, TFAM, TOP2A, TOP2B, TRAP1, UHRF1, VAPA* |
| NUP107 | transporter | 2.99E-04 | *NUP133, NUP98, RANBP2* |
| let-7a-5p  (and other  miRNAs  w/seed  GAGGUAG) | mature microrna | 3.08E-04 | *ATP6V0A1, BSG, COIL, CSNK1D, HMGA1, HRAS, IGF2BP2, KRAS, SLC25A13, SNAP23, UHRF1* |
| FN1 | enzyme | 3.65E-04 | *ADD1, CCT4, CDH1, CSNK2A1, HK1, HLA-B, ITGB1, LIN7C, PCNA, PTBP1, RBBP4, SNAP23, TOMM22* |
| NFE2L2 | transcription regulator | 3.80E-04 | *ATP1A1, CCT3, DYNLL1, EIF2S1, EIF3C, EIF3E, EPB41, GSR, HSP90B1, MGST3, PCBP1, PRDX1, PSMD14, RACK1, RPL18, RPS16, SEC61A1, TFAM, VCP* |
| CDKN1B | kinase | 4.15E-04 | *CCT6A, CDH1, EPB41, EPHA2, LGALS3, MCM5, PCNA, PES1, S100A9* |
| HRAS | enzyme | 4.60E-04 | *AKAP2, ATP5B, CALD1, CDH1, CDH3, EIF3C, FAT1, HLA-B, HRAS, HSPB1, ITGA6, ITGB1, KRAS, MFGE8, NOP58, PCNA, PLOD1, PRNP, RBBP4, RPL30, RPS27, SMARCA2, SPIN1, TOP2A* |
| IL4 | cytokine | 5.11E-04 | *ACOT9, BOP1, CBX3, CCT3, CLNS1A, CPOX, DSG1, EBNA1BP2, EIF2S1, ITGB1, KRR1, LGALS1, LGALS3, LGALS3BP, LIG3, MCM5, MTDH, NOC2L, PHB, PRMT1, PRNP, RCC1, RNPS1, S100A10, S100A8, S100A9, SERPINB4, TPD52L2, UQCRC1, XRCC6* |
| ESRRA | ligand-dependent nuclear receptor | 5.53E-04 | *ACADVL, ATP5B, ENO1, GAPDH, HK1, MTCH2, PKM, TFAM* |
| HGF | growth factor | 5.83E-04 | *AIFM1, ARPC5, CALM1 (includes others), CDH1, DDX21, DSG1, DYNLL1, HK1, ITGA6, ITGB1, KRR1, MCM5, MKI67, MPHOSPH10, NOP2, NUMA1, OCLN, PCNA, PHB, PLOD1, RCC1, S100A9* |
| IGF1R | transmembrane receptor | 6.02E-04 | *ATP5B, ATP5O, CDH1, COX4I1, NDUFA9, PRKCDBP, RPL10A, RPL24, RPL3, RPL35A, SMU1, UQCRC1, UQCRC2* |
| PGR | ligand-dependent nuclear receptor | 6.26E-04 | *CALD1, CD59, CKAP4, DDX21, DYNLL1, HBA1/HBA2, ITGA6, ITGB1, MPHOSPH10, PCNA, PRKG2, S100A8, SLC7A5, SMPDL3B, SRSF7* |
| miR-199a-5p (and other  miRNAs w/seed CCAGUGU) | mature microrna | 6.44E-04 | *CALD1, CDH1, CSTB, ITGA3, LIN7C, MPP5, SET* |
| FLI1 | transcription regulator | 7.30E-04 | *DDX21, HBB, IMP4, RACK1, RPL18, TCP1* |
| OGT | enzyme | 7.57E-04 | *CDH1, COX6C, MTX1, NDUFA9, NDUFS1* |
| BRCA1 | transcription regulator | 8.06E-04 | *CDH3, ELAVL1, GDF15, HBB, HNRNPD, HSPB1, PCBP2, PCNA, RUVBL2, SAFB, XRCC6* |
| E2F4 | transcription regulator | 8.13E-04 | *CALM1 (includes others), HSP90B1, MCM5, MKI67, PCNA, RBBP4, RFC4, SMARCA5, SRRM2, TMPO, TOP2A, TOP2B, UHRF1* |
| VEGFA | growth factor | 1.06E-03 | *ATP5O, COX5B, CSTB, DBT, HK1, ITGB1, OCLN, PCNA, SET, STARD8, TJP2, TOMM20, TOMM22, TOMM70* |
| HSF1 | transcription regulator | 1.08E-03 | *BAG3, CBX3, CCT3, CCT4, CCT5, CCT6A, CCT8, DNAJB1, HSPA4, HSPB1, TCP1* |
| ELAVL1 | other | 1.23E-03 | *CALM1 (includes others), ELAVL1, HNRNPD, RACK1, RPL13, SLC25A11, SRSF7* |
| SAMM50 | other | 1.29E-03 | *MTX1, MTX2* |
| NEO1 | transcription regulator | 1.29E-03 | *CDH1, PCNA* |
| AIFM1 | enzyme | 1.32E-03 | *AIFM1, MT-CO2, NDUFA9* |
| ESR1 | ligand-dependent nuclear receptor | 1.34E-03 | *ADD3, ARHGEF11, ARPC5L, ATP6V1A, BCLAF1, BYSL, CALD1, CD59, CDH1, DDX21, DLG1, DLG5, ENO1, FMR1, HLA-B, HNRNPD, HSP90B1, IMPDH2, ITGA6, KPNB1, KRAS, LGALS1, LGALS3BP, MAP7, MKI67, NUP210, PCM1, PCNA, PES1, PHB, PYCR1, RANBP2, RBBP4, RBM25, RRBP1, S100A9, SLC12A4, SLC7A5, SNAP23, SON, SYNPO, TJP2, TMPO* |
| PKM | kinase | 1.44E-03 | *CDH1, ENO1, ITGB1, PKM* |
| NFYB | transcription regulator | 1.63E-03 | *ATP5B, CDH1, DNAJB6, EIF3C, ELAVL1, GAPDH, LIG3, LYAR, PCNA, RNPS1, SERBP1, SON, TOP2A, UHRF1* |
| PPARGC1A | transcription regulator | 1.70E-03 | *ACADVL, ATP5B, ATP5O, CALM1 (includes others), COX4I1, COX5A, COX5B, COX6C, MT-CO2, NDUFS1, TFAM, UQCRFS1* |
| NME1 | kinase | 1.96E-03 | *ACIN1, BOP1, COIL, NOP58, SF3A1* |
| FMR1 | translation regulator | 2.17E-03 | *CFL1, EEF2, ENO1, GAPDH, UQCRFS1* |
| AK1 | kinase | 2.22E-03 | *ENO1, GAPDH, PKM* |
| FAAH | enzyme | 2.26E-03 | *CORO2A, RPL17, RPL5, RPS18* |
| NF1 | other | 2.26E-03 | *KRAS, RPL10A, RPL21, RPS19* |
| MCU | ion channel | 2.55E-03 | *NDUFA9, UQCRC2* |
| ADD2 | other | 2.55E-03 | *ADD1, ADD3* |
| CTBP1 | enzyme | 2.60E-03 | *CDH1, FAT1, FSCN1, OCLN* |
| SURF1 | enzyme | 2.79E-03 | *COX4I1, MT-CO2, TFAM* |
| STOX1 | other | 2.79E-03 | *CALM1 (includes others), RPL17, SRSF7* |
| PPARGC1B | transcription regulator | 3.20E-03 | *ACADVL, ATP5B, COX4I1, COX5A, ENO1* |
| HTT | transcription regulator | 3.44E-03 | *ATP1A1, ATP5B, ATP5O, COX4I1, CYC1, DNAJB1, EEF2, GAPDH, GSR, HBA1/HBA2, HMGA1, HRAS, ITGB1, MLF2, NDUFA12, NDUFS3, PCNA, PLOD3, PRKCDBP, PTBP1, SLMAP, TFAM, TRAP1, VAPA, YBX1* |
| ROCK2 | kinase | 3.80E-03 | *DSC2, DSG1, S100A8, SPRR3* |
| DYSF | other | 3.95E-03 | *ACOT9, CORO1C, DNAJB1, HSPB1, ITGB1, LGALS3* |
| CTBP2 | transcription regulator | 4.16E-03 | *CDH1, HBB, OCLN* |
| MXI1 | transcription regulator | 4.16E-03 | *IARS, IMPDH2, MKI67* |
| PTGES | enzyme | 4.17E-03 | *CDH1, ITGA6, NOP2, RPS19, RPS3A* |
| CDH4 | other | 4.19E-03 | *CDH1, CDH3* |
| ADAM9 | peptidase | 4.19E-03 | *ITGA3, ITGB1* |
| CD38 | enzyme | 4.58E-03 | *CKAP4, EPS8, LGALS3, MTDH, NDUFV3, PKM, PYCR1, RPN1, SLC7A5* |
| PDGF BB | complex | 4.91E-03 | *ADD3, CALD1, CALM1 (includes others), ELAVL1, GDF15, HRAS, ITGB1, KRAS, LGALS3, PCNA, RACK1, RPL13, SLC25A11, SRSF7* |
| MED30 | transcription regulator | 4.97E-03 | *NDUFA10, NDUFS2, NDUFV1* |
| KRAS | enzyme | 5.44E-03 | *BSG, CDH1, CDH3, CLNS1A, GTF2I, HSPB1, IGF2BP2, KRAS, MSLN, OCLN, TCP1, TOP2A, UQCRC2, VAPA, VCP* |
| IL5 | cytokine | 5.64E-03 | *CKAP4, DDX21, ENO1, EPS8, HSP90B1, MTDH, NDUFV3, PKM, PYCR1, RPN1, SCRIB, SLC7A5, SNAP23* |
| MALSU1 | other | 6.19E-03 | *COX4I1, MT-CO2* |
| CACNA1C | ion channel | 6.19E-03 | *ENO1, S100A10* |
| DLX4 | transcription regulator | 6.19E-03 | *HBB, TOP2A* |
| ENO1 | enzyme | 6.19E-03 | *CDH1, ENO1* |
| PLAUR | transmembrane receptor | 6.55E-03 | *CDH1, ITGA6, ITGB1, PLG* |
| SLC29A1 | transporter | 6.87E-03 | *ITGB1, RPL10A, RPL3* |
| TGFB1 | growth factor | 7.06E-03 | *BSG, CALD1, CALM1 (includes others), CAPRIN1, CCT5, CCT6A, CD59, CDH1, CFL1, CHD4, CORO1C, COX5A, CYC1, DDX21, DNAJB6, DSC2, DYNLL1, EPHA2, FSCN1, GDF15, HMGA1, HSPB1, IARS, ITGA3, ITGA6, ITGB1, KRAS, LGALS3, MELTF, MKI67, NOP58, PCBP1, PCNA, PDLIM7, PLOD1, PPFIBP2, RACK1, RFC4, S100A10, S100A11, SLC12A4, SLC7A5, SRSF3, SRSF6, TARDBP, TJP2, TOP2A, YBX1* |
| miR-296-5p (miRNAs w/seed GGGCCCC) | mature microrna | 7.95E-03 | *HMGA1, PCNA, SCRIB* |
| BDNF | growth factor | 8.17E-03 | *BAG3, EEF1D, EPB41L2, FSCN1, LGALS1, PCNA, PLG, PTBP1, RPL13, RPL35A, RPS23, S100A10, S100A9* |
| KLF3 | transcription regulator | 8.23E-03 | *ACOT9, ARPC4, CCT3, CSNK1G3, HBB, MAK16, MTCH2, NDUFV3, PATJ, PLPP2, RAVER1, SUN1, TES, TMPO* |
| C1orf61 | other | 8.55E-03 | *CDH1, OCLN* |
| MRPL14 | other | 8.55E-03 | *COX4I1, MT-CO2* |
| PCM1 | other | 8.55E-03 | *MKI67, PCNA* |
| Collagen type III | complex | 8.55E-03 | *CDH1, ITGB1* |
| miR-542-3p (miRNAs w/seed GUGACAG) | mature microrna | 8.55E-03 | *RPL11, RPS23* |
| DLC1 | other | 8.55E-03 | *CDH1, S100A10* |
| E2F6 | transcription regulator | 8.92E-03 | *MCM5, RBBP4, RFC4, SRSF1, WDR36* |
| AKT3 | kinase | 9.14E-03 | *COX4I1, MT-CO2, TOMM70* |
| PCGEM1 | other | 9.53E-03 | *ENO1, GAPDH, GSR, PKM* |
| estrogen receptor | group | 9.67E-03 | *CALD1, CD59, CDH1, CDH3, DSC2, HRAS, ITGA3, ITGB1, MT-CO2, OCLN, SYNPO* |
| ARNT | transcription regulator | 9.68E-03 | *ATP5O, CDCP1, EIF6, ENO1, GAPDH, NDUFS2, NDUFS3* |
| SNAI2 | transcription regulator | 1.04E-02 | *BSG, CDH1, ITGA3, ITGB1* |
| AFF4 | transcription regulator | 1.12E-02 | *PTBP1, SRSF6* |
| S100A10 | other | 1.12E-02 | *PLG, S100A10* |
| GAL3ST1 | enzyme | 1.12E-02 | *ITGA6, ITGB1* |
| ALCAM | other | 1.12E-02 | *AMOT, CDH1* |
| EPO | cytokine | 1.21E-02 | *H2AFY, HBA1/HBA2, HBB, HMGN1, ITGB1, KPNB1, PCNA, RPS23, RPS7, SNAP23, SON, YBX1* |
| CYP1A1 | enzyme | 1.21E-02 | *AP2M1, CBX3, LYAR, NDUFV1, RBBP4, TMPO* |
| ETV5 | transcription regulator | 1.22E-02 | *CDH1, ITGB1, PKP4, TJP2* |
| YY1 | transcription regulator | 1.26E-02 | *CBX3, CDH1, MATR3, MCM5, MKI67, PCNA, RBBP4, RPL30, SRSF1, TOP2A, UHRF1* |
| GATA3 | transcription regulator | 1.33E-02 | *AGPAT5, CCDC7, CDH1, FSCN1, PCNA, S100A8, S100A9, SPRR3, TJP2* |
| KDM8 | other | 1.43E-02 | *ENO1, PKM* |
| PTBP1 | enzyme | 1.43E-02 | *KRAS, PKM* |
| RYR1 | ion channel | 1.43E-02 | *GAPDH, MT-CO2* |
| AIMP2 | other | 1.43E-02 | *KRAS, MKI67* |
| MGAT3 | enzyme | 1.43E-02 | *CDH1, ITGA3* |
| ERP29 | transporter | 1.43E-02 | *OCLN, SCRIB* |
| PIK3CA | kinase | 1.43E-02 | *ATP1A1, CALD1, ITGB1, PROCR* |
| MKL1 | transcription regulator | 1.43E-02 | *ARHGEF11, ARPC4, DSG1, ITGA6, ITGB1, S100A8, S100A9* |
| Rb | group | 1.47E-02 | *CDH1, MCM5, PCNA, RFC4, TOP2A* |
| TCOF1 | transporter | 1.47E-02 | *BCLAF1, CAPRIN1, CEBPZ, DNAJC11, S100A16* |
| PRNP | other | 1.47E-02 | *EIF2S1, EPS8, HSP90B1, HSPB1, PRNP* |
| ADAMTS12 | peptidase | 1.49E-02 | *LGALS1, S100A8, S100A9* |
| PTGS2 | enzyme | 1.54E-02 | *CDH1, DYNLL1, ITGA6, MKI67, NOP2, PCNA, RPS19, RPS3A* |
| ADORA2A | g-protein coupled receptor | 1.55E-02 | *CCT5, DAPK3, EEF2, GAPDH, HBA1/HBA2, OCLN, STK38* |
| PML | transcription regulator | 1.55E-02 | *CFL1, DNAJB6, HBB, HSPB1, PCBP2, PRDX1, TPD52L2* |
| VHL | transcription regulator | 1.62E-02 | *ARPC1A, CDCP1, CDH1, CFL1, NOC2L, PCNA, POLRMT* |
| mir-193 | microrna | 1.65E-02 | *FAT1, INF2, SYNPO* |
| CD9 | other | 1.65E-02 | *ITGA3, ITGA6, ITGB1* |
| DNMT3B | enzyme | 1.70E-02 | *CDH1, DNAJB1, RCC1, RPL10A, RPL13, SAFB* |
| NEIL2 | enzyme | 1.76E-02 | *GAPDH, MT-CO2* |
| WWC1 | transcription regulator | 1.76E-02 | *CDH1, OCLN* |
| JMJD6 | transmembrane receptor | 1.76E-02 | *DMAP1, MLF2* |
| CTNND1 | other | 1.76E-02 | *CDH1, CDH3* |
| RASGRF1 | other | 1.76E-02 | *HRAS, KRAS* |
| LGALS1 | other | 1.78E-02 | *HRAS, KRAS, LGALS1, LGALS3* |
| TP63 | transcription regulator | 1.92E-02 | *CDH1, EPHA2, GAPDH, ITGA3, ITGB1, MFGE8, PCNA, PELP1, PPP1R13L, PRNP, PRPF8, RCC1, S100A8, SPRR3* |
| Sos | group | 1.95E-02 | *DLG1, EPS8, GSR, HBA1/HBA2, ITGA6, ITGB1, KRAS, LGALS3BP, RBBP4* |
| IL17C | cytokine | 2.02E-02 | *OCLN, S100A8, S100A9* |
| TERT | enzyme | 2.04E-02 | *AKAP2, CALD1, HLA-B, MFGE8, NOP58, RBBP4, SPIN1* |
| ATF4 | transcription regulator | 2.04E-02 | *CPOX, GDF15, HSP90B1, IARS, LGALS3, PYCR1, SLC7A5* |
| miR-133a-3p (and other  miRNAs w/seed UUGGUCC) | mature microrna | 2.04E-02 | *CORO1C, ELAVL1, FSCN1, PKM* |
| Ggt | group | 2.09E-02 | *SLC7A5* |
| Hdac1/2 | group | 2.09E-02 | *MKI67* |
| CMTM8 | cytokine | 2.09E-02 | *CDH1* |
| ZNF496 | transcription regulator | 2.09E-02 | *HBB* |
| FOXN2 | other | 2.09E-02 | *HBB* |
| REEP5 | transporter | 2.09E-02 | *TOP2B* |
| DNAJC24 | other | 2.09E-02 | *EEF2* |
| SNAI3 | transcription regulator | 2.09E-02 | *CDH1* |
| ZNF503 | other | 2.09E-02 | *CDH1* |
| IMMT | other | 2.09E-02 | *CHCHD6* |
| LOXL3 | enzyme | 2.09E-02 | *CDH1* |
| RGMB | other | 2.09E-02 | *CDH1* |
| FAM73B | other | 2.09E-02 | *H2AFY* |
| DPH2 | other | 2.09E-02 | *EEF2* |
| MTX2 | transporter | 2.09E-02 | *MTX1* |
| MKI67 | other | 2.09E-02 | *CDH1* |
| SETBP1 | other | 2.09E-02 | *SET* |
| EHD1 | other | 2.09E-02 | *ITGB1* |
| SPZ1 | transcription regulator | 2.09E-02 | *PCNA* |
| PPP2R4 | phosphatase | 2.09E-02 | *VCP* |
| F11R | other | 2.09E-02 | *CDH1* |
| CLIP1 | other | 2.09E-02 | *PCNA* |
| AFAP1 | other | 2.09E-02 | *ITGB1* |
| miR-18a-3p (and other  miRNAs w/seed CUGCCCU) | mature microrna | 2.09E-02 | *KRAS* |
| mir-216 | microrna | 2.09E-02 | *YBX1* |
| IQSEC1 | other | 2.09E-02 | *CDH1* |
| SCO2 | other | 2.09E-02 | *COX4I1* |
| ATP5A1 | transporter | 2.09E-02 | *H2AFY* |
| TGFBI | other | 2.09E-02 | *CDH1* |
| BANF1 | other | 2.09E-02 | *S100A9* |
| DISC1 | other | 2.09E-02 | *ITGB1* |
| RRAS2 | enzyme | 2.09E-02 | *CDH1* |
| HOXA4 | transcription regulator | 2.09E-02 | *ITGB1* |
| ZNF184 | other | 2.09E-02 | *S100A9* |
| VPS18 | transporter | 2.09E-02 | *ITGB1* |
| FDCSP | other | 2.09E-02 | *CDH1* |
| SLC5A5 | transporter | 2.09E-02 | *HBB* |
| CYFIP2 | other | 2.09E-02 | *KRAS* |
| LIN7B | other | 2.09E-02 | *LIN7C* |
| PIGA | enzyme | 2.09E-02 | *CD59* |
| LIN7A | other | 2.09E-02 | *LIN7C* |
| ESX1 | transcription regulator | 2.09E-02 | *KRAS* |
| KAT6B | enzyme | 2.09E-02 | *SMARCA2* |
| BAIAP3 | other | 2.09E-02 | *HRAS* |
| RBP2 | transporter | 2.09E-02 | *CDH1* |
| CLCN5 | ion channel | 2.12E-02 | *MKI67, PCNA* |
| VIM | other | 2.12E-02 | *CDH1, ITGB1* |
| GFAP | other | 2.12E-02 | *ITGA6, ITGB1* |
| HNRNPAB | enzyme | 2.12E-02 | *CDH1, HBB* |
| SLC13A1 | transporter | 2.15E-02 | *BYSL, EEF1G, EFTUD2, HSPB1, RPN1* |
| GAST | other | 2.15E-02 | *COX5B, IMPDH2, PHB, RPL35A, RPS19* |
| CD24 | other | 2.21E-02 | *ADD3, BCLAF1, DLG1, GDF15, RANBP2, TOP2A* |
| ERBB2 | kinase | 2.21E-02 | *ATP6V1A, CD59, CDH1, CDH3, DDX10, DNAJB6, EIF6, FSCN1, HSPB1, ITGA6, ITGB1, LGALS1, LGALS3, MKI67, NDUFV1, PCNA, PRKCDBP, RFC4, RPL17, SEC61A1, TOP2A* |
| S100A6 | transporter | 2.22E-02 | *CDH1, PRNP, TMPO* |
| CD28 | transmembrane receptor | 2.38E-02 | *COX6C, CYC1, GAPDH, H2AFY, HMGN1, MSLN, PHB, PUM3, RANBP2, RPL30, RPL6, SLC7A5, XRCC6* |
| CUX1 | transcription regulator | 2.47E-02 | *CDH1, HLA-B, OCLN, PNN* |
| LOXL2 | enzyme | 2.51E-02 | *CDH1, OCLN* |
| TFF3 | other | 2.51E-02 | *CDH1, OCLN* |
| PI3K (family) | group | 2.62E-02 | *CDH1, HK1, ITGA6, ITGB1, PKM* |
| KLF1 | transcription regulator | 2.65E-02 | *BSG, EPB41, HBB* |
| SPP1 | cytokine | 2.86E-02 | *CDH1, ITGA3, NDUFA9, NDUFV1, OCLN, PLOD1, PNN, UQCRC2* |
| Sod | group | 2.92E-02 | *DSC2, DSG1* |
| miR-143-3p (and other  miRNAs w/seed GAGAUGA) | mature microrna | 2.92E-02 | *KRAS, TOP2A* |
| STAT4 | transcription regulator | 2.98E-02 | *AKAP8L, AP2A2, ASPH, BSG, DNAJB6, FSCN1, GLG1, PPP1R13L, SUN1* |
| HIST1H1T | other | 3.15E-02 | *ARPC5, CPOX, HSPA4, SF3A1, TRPM4* |
| LGALS3 | other | 3.15E-02 | *DDOST, FSCN1, ITGA6, ITGB1, KRAS* |
| MAP4K4 | kinase | 3.15E-02 | *ACADVL, CYC1, NDUFS1, NDUFV1, UQCRC1, UQCRFS1* |
| IL2 | cytokine | 3.29E-02 | *CD59, CDH1, DDX21, GAPDH, GDF15, GNL3, HSP90B1, ITGA6, ITGB1, NDUFA12, NOP2, PCNA, PDCD11, PHB, RPL10, RPL21, SNAP23, WDR3* |
| INHBA | growth factor | 3.31E-02 | *CDH1, CHMP4B, COIL, ILF3, KRAS, PCNA, S100A8, S100A9* |
| BCR | kinase | 3.36E-02 | *DSG1, HBB* |
| SERTAD2 | transcription regulator | 3.36E-02 | *CYC1, TFAM* |
| RNA polymerase II | complex | 3.57E-02 | *ATP6V0A1, CDH1, ENO1, GAPDH, GDF15, HBB, PCNA, RBBP4, RPL10A, WDR36* |
| CDKN1A | kinase | 3.58E-02 | *LGALS3, LGALS3BP, MKI67, PCNA, RFC4, TOP2A, UHRF1, XRCC6, YBX1* |
| ITGAV | ion channel | 3.63E-02 | *CDH1, PCNA, VTN* |
| MYOC | other | 3.74E-02 | *DLG1, EPHA2, FSCN1, ITGB1, VAPA* |
| mir-143 | microrna | 3.83E-02 | *CDH1, KRAS* |
| CHEK2 | kinase | 3.83E-02 | *KRAS, OCLN* |
| HNRNPU | transporter | 3.83E-02 | *GAPDH, HBB* |
| KLK5 | peptidase | 3.83E-02 | *S100A8, S100A9* |
| PSEN1 | peptidase | 3.84E-02 | *ATP5B, ATP6V1A, BSG, C1QBP, CFL1, CLTA, EEF1G, ENO1, GAPDH, HBA1/HBA2, HK1, PKM, TOP2B* |
| KLF6 | transcription regulator | 3.87E-02 | *CDH1, MKI67, MSLN, PCNA* |
| MMP2 | peptidase | 3.90E-02 | *ITGA3, OCLN, PLG* |
| NR2F1 | ligand-dependent nuclear receptor | 3.90E-02 | *CDH1, GDF15, VTN* |
| SUZ12 | enzyme | 3.91E-02 | *CDH1, DNAJB6, EIF3A, ILF3, LGALS3, SLC7A5* |
| Collagen type I | complex | 4.08E-02 | *CDH1, ITGA3, ITGB1, S100A10* |
| PLAU | peptidase | 4.08E-02 | *GDF15, PLG, S100A8, S100A9* |
| Rab11 | group | 4.14E-02 | *ITGA6* |
| CASK | kinase | 4.14E-02 | *DLG1* |
| SLC39A6 | transporter | 4.14E-02 | *CDH1* |
| ZNF76 | other | 4.14E-02 | *TCP1* |
| VRK2 | kinase | 4.14E-02 | *TCP1* |
| ALX1 | transcription regulator | 4.14E-02 | *CDH1* |
| SSH2 | phosphatase | 4.14E-02 | *SSH1* |
| TRDN | other | 4.14E-02 | *ASPH* |
| HLTF | transcription regulator | 4.14E-02 | *HBB* |
| PRPH | other | 4.14E-02 | *TARDBP* |
| STXBP4 | other | 4.14E-02 | *ITGB1* |
| MLIP | other | 4.14E-02 | *HK1* |
| FLOT2 | other | 4.14E-02 | *CDH1* |
| TLN1 | other | 4.14E-02 | *ITGB1* |
| miR-3189-3p (miRNAs w/seed CCUUGGG) | mature microrna | 4.14E-02 | *SF3B2* |
| CNTN1 | enzyme | 4.14E-02 | *CDH1* |
| STK38L | kinase | 4.14E-02 | *ITGB1* |
| HPS1 | other | 4.14E-02 | *LGALS3* |
| PNKP | kinase | 4.14E-02 | *MT-CO2* |
| ELP2 | other | 4.14E-02 | *HSPA4* |
| ADGRV1 | g-protein coupled receptor | 4.14E-02 | *HBA1/HBA2* |
| COMP | other | 4.14E-02 | *LGALS3* |
| CBR1 | enzyme | 4.14E-02 | *CDH1* |
| ITGA7 | other | 4.14E-02 | *ITGB1* |
| CXCL14 | cytokine | 4.14E-02 | *CDH1* |
| GPBP1 | transcription regulator | 4.14E-02 | *TOP2A* |
| DPH1 | other | 4.14E-02 | *EEF2* |
| PDIA6 | enzyme | 4.14E-02 | *HSP90B1* |
| CGGBP1 | transcription regulator | 4.14E-02 | *FMR1* |
| FXYD5 | ion channel | 4.14E-02 | *CDH1* |
| CLDN3 | transmembrane receptor | 4.14E-02 | *CDH1* |
| HBG1 | other | 4.14E-02 | *HBA1/HBA2* |
| ILF2 | transcription regulator | 4.14E-02 | *ILF3* |
| RFX4 | transcription regulator | 4.14E-02 | *RPL30* |
| BNIP3 | other | 4.14E-02 | *AIFM1* |
| NDUFAB1 | enzyme | 4.14E-02 | *DBT* |
| NRG1 | other | 4.15E-02 | *ARPC4, CDH1, EPHA2, GLG1, HMGA1, NDUFS1, PLG, PRKG2, XRCC6* |
| ESRRG | ligand-dependent nuclear receptor | 4.18E-02 | *ENO1, GAPDH, PKM* |
| C1QBP | transcription regulator | 4.32E-02 | *C1QBP, MT-CO2* |
| miR-205-5p (and other miRNAs w/seed CCUUCAU) | mature microrna | 4.32E-02 | *ATP1A1, HRAS* |
| TEK | kinase | 4.32E-02 | *S100A8, S100A9* |
| MUC4 | other | 4.32E-02 | *CDH1, VTN* |
| KRT14 | other | 4.47E-02 | *DSC2, DSG1, DSG4* |
| VCAN | other | 4.47E-02 | *CD59, ITGA3, ITGB1, LGALS3, MSLN, SMPDL3B* |
| TFAP2A | transcription regulator | 4.56E-02 | *CDH1, MCM5, PRDX1, RPL6, RPS5* |
| EFNA1 | other | 4.71E-02 | *DSG1, EPHA2, HRAS, KRAS* |
| TFAP4 | transcription regulator | 4.77E-02 | *CDH1, GDF15, OCLN* |
| SIRT1 | transcription regulator | 4.78E-02 | *BCLAF1, CDH1, DDB1, RPL10A, RPL13, SEC61A1, SYNPO, UQCRC2, UQCRFS1* |
| LIN28A | other | 4.83E-02 | *KRAS, TARDBP* |
| PTP4A3 | phosphatase | 4.83E-02 | *CDH1, PCBP1* |
| RFX1 | transcription regulator | 4.83E-02 | *PCNA, RPL30* |
